# Supplementary material for: Emergence of putative energy parasites within Clostridia revealed by genome analysis of a novel endosymbiotic clade
Source: ISME J. 2023 Aug 31;17(11):1895–906. doi: 10.1038/s41396-023-01502-0 (PMC10579323; doi:10.1038/s41396-023-01502-0)
Supplement: Supplementary file 1 — Supplemental Materials [file 41396_2023_1502_MOESM1_ESM.pdf]

Supplementary Text

Supplementary Methods

Whole genome amplification

The collected bacterial cells were transferred to 0.2-mL PCR tube containing 0.4-µL sterile double-distilled water and were subjected to cell lysis and denaturing double strand DNA using 0.5 µL of lysis buffer (0.4 M KOH, 100 mM dithiothreitol, 10 mM EDTA). After neutralization with 0.5 µL of buffer (400 mM HCl, 600 mM Tris-HCl, pH7.5), 10 µL reaction mixture containing 0.2 µL of EquiPhi29 DNA Polymerase, 1 µL of Reaction Buffer in the kit, 0.1 µL of 100 mM DTT, 0.5 µL of 500 µM exo-resistant random primer (Thermo Scientific) and 0.4 µL of 25 mM dNTP (Thermo Scientific) were added, and isothermal whole genome amplification (WGA) was performed at 45°C for 3 h. All plasticware and reagents except for the enzyme and random hexamer were UV-irradiated to decontaminate DNA.

Sequence library preparation for the MinION platform

Before preparing libraries for MinION, we conducted debranching and single-strand DNA digestion of WGA products using EquiPhi29 DNA polymerase and S1 nuclease (Takara Bio, Shiga, Japan), respectively [1]. After the treatment, DNA was purified by ethanol precipitation and fragmented using the Covaris g-TUBE for the RsTa-C01 and CfP3-15 samples. DNA fragments were separated by agarose gel electrophoresis, and those longer than 2 kbp for NkDv07 and 2–12 kbp for RsTa-C01 and CfP3-15 were excised and purified using the Zymoclean Large Fragment DNA Recovery Kit (Zymo Research). Sequencing libraries were then prepared and loaded onto flow cells as described in the main text.

Genome assembly

- RsTa-C01

MiSeq reads were trimmed and quality-filtered using the cutadapt [2] and prinseq [3] programs, respectively. Unpaired reads were removed using cmpfastq_pe (http://compbio.brc.iop.kcl.ac.uk/software/download/cmpfastq_pe). Quality-trimmed reads were then assembled using SPAdes v3.14.1 [4] with '--meta' option. The obtained

contigs were designated “Contig-set 1”. Contig-set 1 was binned into respective bacterial assemblages using MyCC_2017.ova [5] with the “56mer” option. A bin consisted of 28 contigs and containing the 16S rRNA gene of RsTa-C01 was obtained. Among the contigs not included in the RsTa-C01 bin, we detected a high-coverage circular contig (36 kb) with high sequence similarity to certain contigs in the RsTa-C01 bin. Based on the gene repertories of this circular contig, we inferred that it was the complete genome sequence of a phage infecting RsTa-C01. We considered that the high sequence similarity between this phage genome and certain RsTa-C01 genome regions caused the low contiguity of the RsTa-C01 genome assembly. We therefore attempted re-assembling reads into contigs as above after excluding reads showing high sequence similarity to the phage genome. Obtained contigs were again binned as above, and the results were refined using Graphbin v1.2 [6]. We further detected and masked regions sharing identity with the phage genome by BLASTn searches with an e-value threshold of 10^{-4}, using a script (https://github.com/tkhsgani/blast_masking.py). The resulting RsTa-C01 bin without the regions showing sequence similarities to the phage genome was designated “Contig-set 2”.

MinION reads after adapter trimming were filtered using Nanofilt v2.7.1 [7] with the “-q 13 -l 1000 --maxlength 20000 --headcrop 75” option, and chimeric reads were removed using yacrd v0.6.2 [8] with the “-c 4 -n 0.4 scrubb” option. Obtained reads ≥ 1 kb were mapped onto Contig-set 2 using Minimap2 v2.17 [9], and the mapped long reads were assembled into contigs using Flye v2.8 [10]. The resulting RsTa-C01 contig was circular, but certain regions were not covered with the long reads. To identify misassembled regions, we used Raven v1.6.0 [11] to create another assembly from the above mapped reads, and the results were compared using Nucmer v3.1 [12]. Regions showing discrepancy were replaced by contigs of the Raven assembly using a script (https://github.com/dkato2021/get-substituted-sequences), and we confirmed that the resulting circular contig was fully covered by the long reads. Furthermore, regions where discrepancy with Contig-set 1 was found were replaced using Nucmer v3.1 and the above script (https://github.com/dkato2021/get-substituted-sequences). Finally, the contig sequence was polished with the quality-trimmed MiSeq reads using Pilon v1.23 [13] with one iteration.

- CfP3-15

Assembly of MiSeq reads and binning were performed as in the genome of RsTa-C01. The MinION reads after adapter trimming were filtered using Nanofilt v2.7.1 with the "-q 13 -l 3000 --maxlength 20000 --headcrop 75" options, and palindromes introduced by whole-genome amplification were corrected with Pacasus (downloaded on 2020/09) [14] with the "--filter_factor=0.01 --query_coverage=0.01 --query_identity=0.01 --relative_score=0.01 --base_score=1.0" options. From the obtained reads ≥ 5 kb, chimeric reads were removed using yacrd v0.6.2 with the "-c 4 -n 0.4 scrubb" option. Obtained reads were assembled into contigs using Flye v2.8. From the contigs ≥ 10 kb, the CfP3-15 contigs were recovered by BLASTn searches using the above short read-derived CfP3-15 bin as queries. The resulting contigs were manually checked by BLASTn searches of the KEGG GENOME database and also by CAT v5.1.2 [15] annotation, and then sequences identified as non-*Clostridia* were excluded. The long reads were mapped to these CfP3-15 contigs using Minimap2 v2.17 and the mapped reads were recovered. This mapping revealed that this sample has a high degree of CfP3-15 genome-wide variation in read coverage, and therefore it was subsampled to read coverage of 200 using samNormalize.pl [16]. Subsampled reads were reassembled using Flye v2.8, and a circular CfP3-15 contig was obtained.

The quality-filtered MiSeq reads were mapped to this circular CfP3-15 contig, and mapped reads were recovered using Bowtie2 v2.4.1 [17]. The mapped reads were assembled using SPAdes v3.14.1 with the "--sc" and "--trusted-contigs" options, with the CfP3-15 circular contig being specified as the trusted contig. Contigs derived from this SPAdes assembly were used to replace regions of the circular CfP3-15 contig by using Nucmer v3.1 and the above script for substitution, where discrepancy was found. Finally, the contig was polished using Pilon v1.23 with one iteration.

- NkDv07

Assembly of MiSeq reads and binning were performed as in the genome of RsTa-C01, and the resulting NkDv07 bin was similarly designated "Contig-set 1". MinION reads were processed using Nanofilt v2.7.1 with "-q 7 -l 500 --maxlength 20000 --headcrop 75". The long reads were assembled into contigs using Flye v2.8, and NkDv07 contigs were

recovered by BLASTn searches using Contig-set 1 as queries. Recovered contigs were manually checked by BLASTn searches of the KEGG GENOME database, and non-*Clostridia* sequences were excluded. The resulting contigs were cut at misassembled points using Tigmint v1.2.5 [18] with the "tigmint-make tigmint-long span=auto G=1.2e6 dist=auto" options and then scaffolded using LINKS v2.0.0 [19] with the "-d 1000, 2000, 3000, 4000" option, SLR [20] and LongStitch v1.0.1 [21]. The scaffolds were merged using CAMSA v1.3 [22], and gaps in the scaffolds were filled using TGS-GapCloser v1.1.1 [23]. The sequences were polished using Racon v1.4.16 [24] with two iterations and medaka v1.0.3 (https://github.com/nanoporetech/medaka). The resulting contigs were manually cut based on the results of mapping the long and short reads. Regions of the contigs were replaced by comparing them with Contig-set 1 using Nucmer v3.1 and the above script for substitution. The resulting contigs were then polished using Pilon v1.23 with three iterations with default settings and one additional iteration with the "--unpaired" option.

The resulting contig set contained a contig (~11 kb) that appeared to be chimera composed of several regions on other contigs, and indeed few MiSeq reads were mapped onto the contig. Therefore, the contig was discarded. Finally, regions covered with only a few reads were verified by PCR amplification and Sanger sequencing.

<u>Circular map of CfP3-15 and RsTa-C01</u>

Circular maps of the two complete genomes of the endosymbiotic *Clostridia* were depicted using Proksee (https://proksee.ca/). GC content and GC skew were calculated using a window size of 100,000 and a step size of 100. OriC and Ter sites were predicted using Ori-Finder 2022 [25].

<u>Detection of 16S rRNA gene in the MAG of GCA_90054335.1</u>

We were able to find 16S rRNA genes ≥ 500 bp only from two MAGs of the small genome clade, i.e., MGYG000003453 and GCA_900554335.1. Whereas the 16S rRNA gene of MGYG000003453 formed a monophyletic cluster with those of RsTa-C01, CfP3-15 and NkDv07 in consistence with the phylogenomic tree shown in Fig. 5A, the gene of GCA_900554335.1 did not. We suspected that the 16S rRNA gene of GCA_900554335.1 had possibly been misassembled in a previous study [26], and therefore, we reassembled

the reads using SPAdes v3.14.1 with the "--meta" option, and resulting contigs were binned using MetaBat2 v2.12.1 [27]. BinSPreader [28] was used to refine the binning result, and the "--read" option was used to retrieve the read set of the bin corresponding to GCA_900554335.1. The read set was reassembled using SPAdes v3.14.1 with the "--only-assembler" option, and the 16S rRNA gene was detected in the obtained contigs using Barrnap v0.9 (https://github.com/tseemann/barrnap). The newly assembled and binned 16S rRNA gene formed a monophyletic cluster with the other four genes as shown in Fig. 2.

Re-binning of GCA_009785985.1

The MAG of GCA_009785985.1 was placed in the small genome clade by phylogenomic analysis, but its genome size was unusually large (ca. 2.7 Mbp) compared to the other MAGs in this clade. We suspected that the MAG probably contained contaminating contigs, and indeed, our CAT v.5.1.2-based contig classification showed that 239 of the 648 contigs were not assigned to the phylum *Bacillota*. We therefore re-binned the contigs using MyCC with the "56mer" option, and as a result, a 1.2-Mbp MAG consisting mostly of *Clostridia*-related contigs was obtained and used for the subsequent analyses.

Eukaryotic-like gene detection and signal peptide prediction

The proteins encoded by the small genome clade were used as queries of BLASTp searches of the NCBI non-redundant (nr) database from eukaryotes. The query proteins that showed significant sequence similarity with e-value of 10^{-4} were then used as queries of DIAMOND BLASTp v2.0.13 [29] searches of the NCBI nr database with the "--masking seg --ultra-sensitive" options. The protein sequences from members of the small genome clade present in the NCBI database (i.e., GCA_000435275.1, GCA_009785985.1, GCA_017406805.1, GCA_017414725.1, GCA_017431755.1, GCA_017432165.1, GCA_017448285.1, GCA_017480265.1, GCA_017515685.1, GCA_017634155.1) were ignored. When protein sequences showed the highest similarity to those from eukaryotes or when 90% of the detected database sequences were from eukaryotes, they were selected as candidates of eukaryotic-like proteins. These candidates were then used as queries for NCBI Conserved Domain Search (CD-search) in the web server (2022/09/19), and detected domains were selected as candidates of eukaryotic-like

domains. Based on the NCBI and InterPro databases, the eukaryotic-like domain candidates were manually checked, and domains that are common also in bacteria and/or archaea were removed. The remaining domains were regarded as eukaryotic-like domains. Finally, local CD-search was performed using RPS-BLAST of Conserved Domain Database v3.20 for all proteins encoded by the small genome clade, and the proteins showing the highest similarity to eukaryotic-like domains were designated as eukaryotic-like proteins. Local CD-search was also performed for other genomes of "Acutalibacteraceae", and eukaryotic-like proteins were selected in the same way based on the eukaryotic-like domains specified above.

For the identified eukaryotic-like genes of CfP3-15, NkDv07, and RsTa-C01, signal peptides were predicted using SignalP 6.0 [30].

Phylogenetic analysis including short 16S rRNA gene amplicon sequences

The 16S rRNA gene amplicon sequences obtained by Levy and Jami (2018) [31] and Utami et al. (2018) [32] were placed onto the maximum likelihood tree shown in Fig. S2B, using EPA-NG v0.3.8 [33]. EPA-NG performs phylogenetic placement based on maximum likelihood, and each sequence can be assigned to multiple places of the tree with different likelihoods. We obtained a tree, where each amplicon sequence was represented on the position with the highest likelihood on the tree, using GAPPA v.0.8.3 [34] with the "graft --fully-resolve" option.

Tree visualization

Phylogenetic trees were visualized using the ggtree R package [35].

Reconstruction of other MAGs and statistics

The quality-trimmed reads of each sample were assembled using SPAdes v3.14.1 with the "--sc" option and binned using MyCC with the default mode. The completeness of reconstructed bins was estimated using CheckM [36] and also CheckM2 [37]. The MAGs with CheckM or CheckM2 completeness >50% were taxonomically classified using GTDB-tk v2.1.0 [38] with the r207 database. Reads alignment rates for each MAG were calculated using Bowtie2 v2.4.1.

Supplementary Results

16S rRNA gene cloning analysis of bacterial microbiota associated with single protist cells

During the sequencing analysis of 16S rRNA genes amplified by PCR from a suspension of ten *Trichonympha agilis* cells in our previous study [39], six identical sequences among 46 clones were assigned to the class *Clostridia* and were designated as phylotype RsTa-C01. The remaining clones comprised 16 sequences of the dominant obligate endosymbiont "*Candidatus* Endomicrobium trichonymphae" which supplements nitrogenous compounds [40-42], 20 sequences of the dominant hydrogen-oxidizing obligate ectosymbiont "*Candidatus* Desulfovibrio trichonymphae" [39, 43], and four minor, facultative *Mycoplasmatales* symbionts [44].

16S rRNA phylotype CfP3-15 was identified during preliminary sequencing analyses of 16S rRNA genes amplified by PCR from single cells of *Pseudotrichonympha grassii* for the previous genome analysis of "*Candidatus* Azobacteroides trichonymphae" [45]. In a single-cell WGA sample of *P. grassii*, five of 93 clones were identical sequences belonging to the class *Clostridia* and were designated as phylotype CfP3-15. The remaining clones comprised the predominant, dinitrogen-fixing obligate endosymbiont "*Ca.* Azobacteroides trichonymphae" [45-47] and a clone related to "*Candidatus* Ancillula" [48, 49].

16S rRNA phylotype NkDv07 was identified during preliminary sequencing analyses of 16S rRNA genes amplified by PCR from single cells of the largest morphotype among multiple *Devescovina* species in the gut of *Neotermes sugioi* [50] for our unpublished genome analysis of "*Candidatus* Armantifilum devescovinae". Three identical sequences among 95 clones were assigned to the class *Clostridia* and were designated as phylotype NkDv07. The remaining clones comprised the predominant, dinitrogen-fixing ectosymbiont "*Candidatus* Armantifilum devescovinae" [50-53], 14 clones related to "*Candidatus* Nucleococcus" (phylum *Verrucomicrobiota*) [54], and three clones of the order *Holosporales.*

Other MAGs detected in WGA samples

During assembling and binning, several other MAGs in addition to the clostridial ones were recovered and listed in Table S8. Most of those MAGs were assigned to either of "*Ca*. Azobacteroides", *Endomicrobium* or *Spirochaetales* (*Termitinemataceae* and *Treponemataceae*). Among them, "*Ca.* Azobacteroides pseudotrichonymphae" and "*Ca.* Endomicrobium trichonymphae" are previously known mutualistic, permanently-associated endosymbionts of *Pseudotrichonympha grassii* and *Trichonympha agilis*, respectively [42, 45]. Any MAGs were not shared by different WGA samples, indicating these MAGs were not contaminants in reagents. Members of *Spirochaetales* have frequently been detected as ectosymbionts of termite gut protists [55-57].

The functions of hypothetical proteins OG0003398, OG0004343, and OG0005234

We attempted to predict the functions of the hypothetical proteins for which the gene flux analysis showed that the last common ancestor of the small genome clade gained. The functional prediction was conducted based on sequence homology using the NCBI Conserved Domain Search and structural homology using AlphaFold2 [58] and Dali server [59]. However, the NCBI Conserved Domain Search did not show significantly homologous domains, and AlphaFold2 also did not predict the structure with a high confidence score. Therefore, the functions of the hypothetical proteins were currently unknown.

Description of '*Candidatus* Improbicoccus' gen. nov

Improbicoccus [L. masc. adj. *improbus*, disloyal, wicked; M.L. m. n. *coccus*, from Gr. a berry, coccus; N.L. m. n. *Improbicoccus*, a parasitic coccus]. The bacteria specifically colonize the cytoplasm of protist cells in the termite guts. They are cocci of 1–2 μm and have a Gram-positive type cell wall. They are obligate intracellular parasites and have not been cultured. The type species is '*Candidatus* Improbicoccus pseudotrichonymphae', corresponding to CfP3-15, with its genome sequence as the type material.

Description of '*Candidatus* Paraimprobicoccus' gen. nov

Paraimprobicoccus [Gr. prep. para, alike, alongside of; N.L. m. n. *Improbicoccus*, taxonomic name of a bacterial genus; N.L. m. n. *Improbicoccus*, taxonomic name of a bacterial *Improbicoccus*-alike genus]. The bacteria have cell structure and metabolic capacity similar to *Candidatus* Improbicoccus. ANI and AAI between this genus and *Candidatus* Improbicoccus are below 70% and 50%, respectively. The type species is '*Candidatus* Paraimprobicoccus trichonymphae', corresponding to RsTa-C01, with its genome sequence as the type material.

Description of '*Candidatus* Improbicoccus pseudotrichonymphae' sp. nov

Improbicoccus pseudotrichonymphae (pseudo. tri.cho.nym'phae. N.L. n. *Pseudotrichonympha*, a genus of flagellated protists; N.L. gen. pseudotrichonymphae, of *Pseudotrichonympha*, referring to the host genus). The fundamental attributes of this species are the same as those for the genus. The bacterium colonizes the cytoplasm of the protist *P. grassii* in the gut of the termite *Coptotermes formosanus*. The cell dimensions are 0.5–1.0 μm by 0.5–1.0 μm. The assignment is based on the 16S rRNA gene, the complete genome (AP027924), and hybridization with the 16S rRNA-targeted probe CfP3-15-656 (5′-CCGTTCGCCTCTACTTTAC-3′). This species corresponds to CfP3-15.

Description of '*Candidatus* Improbicoccus devescovinae' sp. nov

Improbicoccus devescovinae (de.ves.co.vi'nae. N.L. fem. n. *Devescovina*, a genus of flagellated protists; N.L. gen. fem. n. devescovinae, of *Devescovina*, referring to the host genus). The fundamental attributes of this species are the same as those for the genus. The bacterium colonizes the cytoplasm of the protist *Devescovina* sp. in the gut of the termite *Neotermes sugioi*. The cell dimensions are 1.0–2.0 μm by 1.0–2.0 μm and the cells usually form an aggregate. The assignment is based on the 16S rRNA gene, the draft genome (BSWA01000001-BSWA01000004), and hybridization with the 16S rRNA-targeted probe NkDv07-142 (5′-TCCATCAGCTATCTCCCAC-3′). This species corresponds to NkDv07.

Description of '*Candidatus* Paraimprobicoccus trichonymphae' sp. nov

Paraimprobicoccus trichonymphae (tri.cho.nym'phae. N.L. fem. n. *Trichonympha*, a genus of flagellated protists; N.L. gen. fem. n. Trichonymphae, of *Trichonympha*, referring to the host genus). The fundamental attributes of this species are the same as those for the genus. The bacterium colonizes the cytoplasm of the protist *Trichonympha agilis* in the gut of termite *Reticulitermes speratus*. The cell dimensions are 1.0–2.0 µm by 1.0–2.0 µm and form diplococci. The assignment is based on the 16S rRNA gene, the complete genome (AP027925), and hybridization with the 16S rRNA-targeted probe RsTa-C01-76 (5′-GTGCCTTGCAAAACTCCG-3′). This species corresponds to RsTa-C01.

Legends to Supplementary Figures

Figure S1. Fluorescence in situ hybridization analysis of 16S rRNA phylotype NkDv07 (**A**, **B**) and transmission electron micrographs of intracellular bacterial symbionts of *Pseudotrichonympha grassii* (**C**–**E**). Phase contrast image of *Devescovina* sp. from the gut of *Neotermes sugioi* (**A**) and NkDv07 cells detected using 6FAM-labelled probe NkDv07-142 (green) as single cocci (**B**). Amorphous yellowish autofluorescence in **B** was emitted from wood particles ingested by *Devescovina* sp. Putative clostridial cell (C), and "*Candidatus* Azobacteroides pseudotrichonymphae" cells (D). In panel **E**, a putative clostridial cell apparently during fission was in the center and a "*Ca.* A. pseudotrichonymphae" cell in the lower right. Bars: **A** and **B**, 10 µm; **C**, 100 nm; **D**, 200 nm; **E**, 500 nm.

Figure S2. Phylogenetic positions of endosymbiotic *Clostridia* based on 16S rRNA gene sequences. (**A**) Maximum-likelihood tree of endosymbiotic *Clostridia* and other members of the family *Oscillospiraceae*. Described species of *Oscillospiraceae* were included in the tree. The tree was constructed with the GTR+I+G4 nucleotide substitution model and rooted with *Syntrophomonas wolfei* (CP000448) and *Thermosyntropha lipolytica* (FQWY01000001) as the outgroup. Clusters of *Clostridium* [60] and the clade corresponding to "Acutalibacteraceae" in the GTDB taxonomy are highlighted in gray and red, respectively. (**B**) Maximum-likelihood tree of endosymbiotic *Clostridia* and other members of the "Acutalibacteraceae". Uncultured clones closely related to described species and endosymbiotic *Clostridia* were included. The tree was constructed with the GTR+I+G4 nucleotide substitution model and rooted with *Sporobacter termitidis* (Z49863) and *Oscillibacter ruminantium* (JF750939) as the outgroup. Highly supported nodes (SH-aLRT value ≥ 80% and ultrafast bootstrap support value ≥ 95%) are highlighted with a closed circle.

Figure S3. Relationships between the size and GC content of the complete genomes in the class *Clostridia*.

Figure S4. Circular representation of the CfP3-15 chromosome (**A**) and the RsTa-C01 chromosome (**B**). The rings denote the following features (from inside): (i) Scale in mega base pairs of the chromosome (black); (ii) GC content (black); (iii) GC skew (green and purple); (iv) predicted genes present on the forward strand (+); (v) genes present on the reverse strand (-). OriC and Ter sites are indicated by arrows in **A**. For the RsTa-C01 genome, we could not detect these sites, using Ori-Finder 2022.

Figure S5. Comparison of the number (**A**) and ratio (**B**) of genes classified into clusters of orthologous genes (COG) functional categories. The genomes of CfP3-15, NkDv07, RsTa-C01, and their free-living relatives *Clostridium leptum* and *Ruminococcus bromii* were compared. The categories denote the following functions: (A) RNA processing and modification; (B) chromatin structure and dynamics; (C) energy production and conversion; (D) cell cycle control, cell division, chromosome partitioning; (E) amino acid transport and metabolism; (F) nucleotide transport and metabolism; (G) carbohydrate transport and metabolism; (H) coenzyme transport and metabolism; (I) lipid transport and metabolism; (J) translation, ribosomal structure and biogenesis; (K) transcription; (L) replication, recombination and repair; (M) cell wall/membrane/envelope biogenesis; (N) cell motility; (O) posttranslational modification, protein turnover, chaperones; (P) inorganic ion transport and metabolism; (Q) secondary metabolites biosynthesis, transport and catabolism; (R) general function prediction only; (S) function unknown; (T) signal transduction mechanisms; (U) intracellular trafficking, secretion, and vesicular transport; (V) defense mechanisms; (W) extracellular structures; (X) mobilome: prophages, transposons; (Y) nuclear structure; (Z) cytoskeleton.

Figure S6. Comparison of the number (**A**) and ratio (**B**) of pseudogenes classified into clusters of orthologous genes (COG) functional categories. See also the legend to Fig. S5.

Figure S7. Amino acid sequence alignment of ATP/ADP translocase and other nucleotide transport proteins from the endosymbiotic *Clostridia*, other parasitic bacteria, and plastids. Conserved residues important for substrate specificity, transport efficiency, and counter exchange of ATP/ADP translocase (CAA89201.2) of the plastid of *Arabidopsis thaliana* are indicated with arrows.

Figure S8. RT-PCR analysis of the ATP/ADP translocase gene. Agarose gel electrophoresis of RT-PCR products from total RNA of *R. speratus* guts (**A**) and *C. formosanus* guts (**B**). N, negative control (RNA samples without reverse transcription); P, products of RT-PCR using primer sets specific to the respective ATP/ADP translocase genes (see Table S2). These bands were excised from gels, and their sequences were verified by cloning and Sanger sequencing analysis.

Figure S9. Phylogenetic positions of eukaryotic-like genes contained in the genomes of CfP3-15, NkDv07, and RsTa-C01. The following genes were used as queries to identify homologues as reference sequences. (**A**) CfP3-15_0049. (**B**) CfP3-15_0165. (**C**) CfP3-15_0235. (**D**) CfP3-15_0282. (**E**) CfP3-15_0445. (**F**) CfP3-15_0456. (**G**) CfP3-15_0548. (**H**) CfP3-15_0552. (**I**) CfP3-15_0565. (**J**) CfP3-15_0581. (**K**) CfP3-15_0591. (**L**) CfP3-15_0627. (**M**) CfP3-15_0653. (**N**) CfP3-15_0780. (**O**) CfP3-15_0790. (**P**) CfP3-15_0852. (**Q**) CfP3-15_0856. (**R**) CfP3-15_0934. (**S**) NkDv07_0114. (**T**) NkDv07_0121. (**U**) NkDv07_0512. (**V**) NkDv07_0536. (**W**) NkDv07_0615. (**X**) RsTa-C01_0144. (**Y**) RsTa-C01_0199. (**Z**) RsTa-C01_0208. (**AA**) RsTa-C01_0431. (**AB**) RsTa-C01_0774. (**AC**) RsTa-C01_0775. (**AD**) RsTa-C01_0992. Highly supported nodes (SH-aLRT value ≥ 80% and ultrafast bootstrap support value ≥ 95%) are highlighted with closed circles. The sequence IDs highlighted by green, blue, and light blue correspond to the proteins of the endosymbiotic *Clostridia* used to find homologs, proteins of the protist hosts, and proteins from parabasalid protists, respectively.

Figure S10. RT-PCR analysis of eukaryotic-like protein-coding genes. Agarose gel electrophoresis of RT-PCR products from total RNA of *R. speratus* guts (**A**) and *C. formosanus* guts (**B**). N, negative control (RNA samples without reverse transcription); P, products of RT-PCR using primer sets specific to the respective eukaryotic-like protein-coding genes (see Table S2). The amplification products of eukaryotic-like protein-coding genes are indicated by arrows, which were confirmed by Sanger sequencing.

Figure S11. Comparison of a eukaryotic-like gene, CfP3-15_0552, in the CfP3-15 genome and that obtained by RT-PCR from *C. formosanus* guts. (**A**) Nucleotide

sequences. (**B**) Amino acid sequences of **A**.

Figure S12. Pairwise ANI (**A**) and AAI (**B**) heatmap among the genomes in the small genome clade. The phylogenetic tree of the small genome clade is shown. In **B**, the genus-level clade based on GTDB r207 are indicated.

Figure S13. Principal component analysis of genomes of "Acutalibacteraceae" (Table S3) based on the relative abundance of the COG functional categories (**A**) and comparison of the number (**B**) and ratio (**C**) of the COG functional categories. Genomes belonging to the small genome clade are highlighted with red. Arrows indicate the loadings of each functional category. Several arrows are omitted to improve visibility. See also the legend to Fig. S5.

Figure S14. Distribution of genes acquired by the last common ancestor of the small genome clade, predicted in the gene flux analysis. The phylogenetic tree corresponds to that shown in Fig. 5A with the small genome clade being shaded in red.

Figure S15. Comparison between the phylogenomic tree of the small genome clade and the gene tree of their ATP/ADP translocase. (**A**) Comparison of the *Clostridia* ATP/ADP translocase I (left) with the phylogenomic tree (right). (**B**) Comparison of the *Clostridia* ATP/ADP translocase II (left) and the phylogenomic tree (right). Highly supported nodes (SH-aLRT value ≥ 80% and ultrafast bootstrap support value ≥ 95%) are indicated with a closed circle.

Figure S16. Phylogenetic positions of 16S rRNA gene amplicon sequences derived from the protistan fraction of a cattle rumen [31] and the guts of termites and cockroaches [32].

References to Supplementary Materials

1. Zhang K, Martiny AC, Reppas NB, Barry KW, Malek J, Chisholm SW, et al. Sequencing genomes from single cells by polymerase cloning. Nat Biotechnol. 2006;24:680-6.
2. Martin M. Cutadapt removes adapter sequences from high-throughput sequencing reads. EMBnet J. 2011;17:10.
3. Schmieder R, Edwards R. Quality control and preprocessing of metagenomic datasets. Bioinformatics. 2011;27:863-4.
4. Bankevich A, Nurk S, Antipov D, Gurevich AA, Dvorkin M, Kulikov AS, et al. SPAdes: a new genome assembly algorithm and its applications to single-cell sequencing. J Comput Biol. 2012;19:455-77.
5. Lin HH, Liao YC. Accurate binning of metagenomic contigs via automated clustering sequences using information of genomic signatures and marker genes. Sci Rep. 2016;6:e24175.
6. Mallawaarachchi V, Wickramarachchi A, Lin Y. GraphBin: refined binning of metagenomic contigs using assembly graphs. Bioinformatics. 2020;36:3307-13.
7. De Coster W, D'Hert S, Schultz DT, Cruts M, Van Broeckhoven C. NanoPack: visualizing and processing long-read sequencing data. Bioinformatics. 2018;34:2666-9.
8. Marijon P, Chikhi R, Varre JS. yacrd and fpa: upstream tools for long-read genome assembly. Bioinformatics. 2020;36:3894-6.
9. Li H. Minimap2: pairwise alignment for nucleotide sequences. Bioinformatics. 2018;34:3094-100.
10. Kolmogorov M, Yuan J, Lin Y, Pevzner PA. Assembly of long, error-prone reads using repeat graphs. Nat Biotechnol. 2019;37:540-6.
11. Vaser R, Šikić M. Time- and memory-efficient genome assembly with Raven. Nat Comput Sci. 2021;1:332-6.
12. Kurtz S, Phillippy A, Delcher AL, Smoot M, Shumway M, Antonescu C, et al. Versatile and open software for comparing large genomes. Genome Biol. 2004;5:R12.
13. Walker BJ, Abeel T, Shea T, Priest M, Abouelliel A, Sakthikumar S, et al. Pilon: an integrated tool for comprehensive microbial variant detection and genome assembly improvement. PLoS One. 2014;9:e112963.
14. Warris S, Schijlen E, van de Geest H, Vegesna R, Hesselink T, Te Lintel Hekkert

B, et al. Correcting palindromes in long reads after whole-genome amplification. BMC Genom. 2018;19:e798.
15. Von Meijenfeldt FAB, Arkhipova K, Cambuy DD, Coutinho FH, Dutilh BE. Robust taxonomic classification of uncharted microbial sequences and bins with CAT and BAT. Genome Biol. 2019;20:e217.
16. Eccles D. gringer/bioinfscripts: Tree Lab / Global River Release (1.3.0-TRL). 2019.
17. Langmead B, Salzberg SL. Fast gapped-read alignment with Bowtie 2. Nat Methods. 2012;9:357-9.
18. Jackman SD, Coombe L, Chu J, Warren RL, Vandervalk BP, Yeo S, et al. Tigmint: correcting assembly errors using linked reads from large molecules. BMC Bioinform. 2018;19:e393.
19. Warren RL, Yang C, Vandervalk BP, Behsaz B, Lagman A, Jones SJ, et al. LINKS: Scalable, alignment-free scaffolding of draft genomes with long reads. Gigascience. 2015;4:e35.
20. Luo J, Lyu M, Chen R, Zhang X, Luo H, Yan C. SLR: a scaffolding algorithm based on long reads and contig classification. BMC Bioinform. 2019;20:e539.
21. Coombe L, Li JX, Lo T, Wong J, Nikolic V, Warren RL, et al. LongStitch: high-quality genome assembly correction and scaffolding using long reads. BMC Bioinform. 2021;22:e534.
22. Aganezov SS, Alekseyev MA. CAMSA: a tool for comparative analysis and merging of scaffold assemblies. BMC Bioinform. 2017;18:e496.
23. Xu M, Guo L, Gu S, Wang O, Zhang R, Peters BA, et al. TGS-GapCloser: A fast and accurate gap closer for large genomes with low coverage of error-prone long reads. Gigascience. 2020;9:giaa094.
24. Vaser R, Sovic I, Nagarajan N, Sikic M. Fast and accurate de novo genome assembly from long uncorrected reads. Genome Res. 2017;27:737-46.
25. Dong MJ, Luo H, Gao F. Ori-Finder 2022: A comprehensive web server for prediction and analysis of bacterial replication origins. Genomics Proteomics Bioinformatics. 2022;20:1207-13.
26. Almeida A, Mitchell AL, Boland M, Forster SC, Gloor GB, Tarkowska A, et al. A new genomic blueprint of the human gut microbiota. Nature. 2019;568:499-504.
27. Kang DD, Li F, Kirton E, Thomas A, Egan R, An H, et al. MetaBAT 2: an adaptive binning algorithm for robust and efficient genome reconstruction from metagenome assemblies. PeerJ. 2019;7:e7359.
28. Tolstoganov I, Kamenev Y, Kruglikov R, Ochkalova S, Korobeynikov A.

BinSPreader: Refine binning results for fuller MAG reconstruction. iScience. 2022;25:e104770.

29. Buchfink B, Reuter K, Drost HG. Sensitive protein alignments at tree-of-life scale using DIAMOND. Nat Methods. 2021;18:366-8.
30. Teufel F, Almagro Armenteros JJ, Johansen AR, Gislason MH, Pihl SI, Tsirigos KD, et al. SignalP 6.0 predicts all five types of signal peptides using protein language models. Nat Biotechnol. 2022;40:1023-5.
31. Levy B, Jami E. Exploring the prokaryotic community associated with the rumen ciliate protozoa population. Front Microbiol. 2018;9:e2526.
32. Utami YD, Kuwahara H, Murakami T, Morikawa T, Sugaya K, Kihara K, et al. Phylogenetic diversity and single-cell genome analysis of "*Melainabacteria*", a non-photosynthetic cyanobacterial group, in the termite gut. Microbes Environ. 2018;33:50-7.
33. Barbera P, Kozlov AM, Czech L, Morel B, Darriba D, Flouri T, et al. EPA-ng: Massively parallel evolutionary placement of genetic sequences. Syst Biol. 2019;68:365-9.
34. Czech L, Barbera P, Stamatakis A. Genesis and Gappa: processing, analyzing and visualizing phylogenetic (placement) data. Bioinformatics. 2020;36:3263-5.
35. Yu G, Smith DK, Zhu H, Guan Y, Lam TTY, McInerny G. ggtree: an R package for visualization and annotation of phylogenetic trees with their covariates and other associated data. Methods Ecol Evol. 2016;8:28-36.
36. Parks DH, Imelfort M, Skennerton CT, Hugenholtz P, Tyson GW. CheckM: assessing the quality of microbial genomes recovered from isolates, single cells, and metagenomes. Genome Res. 2015;25:1043-55.
37. Chklovski A, Parks DH, Woodcroft BJ, Tyson GW. CheckM2: a rapid, scalable and accurate tool for assessing microbial genome quality using machine learning. bioRxiv. 2022.
38. Chaumeil PA, Mussig AJ, Hugenholtz P, Parks DH. GTDB-Tk: a toolkit to classify genomes with the Genome Taxonomy Database. Bioinformatics. 2019;36:1925-7.
39. Sato T, Hongoh Y, Noda S, Hattori S, Ui S, Ohkuma M. *Candidatus* Desulfovibrio trichonymphae, a novel intracellular symbiont of the flagellate *Trichonympha agilis* in termite gut. Environ Microbiol. 2009;11:1007-15.
40. Stingl U, Radek R, Yang H, Brune A. "*Endomicrobia*": Cytoplasmic symbionts of termite gut protozoa form a separate phylum of prokaryotes. Appl Environ Microbiol. 2005;71:1473-9.

41. Ohkuma M, Sato T, Noda S, Ui S, Kudo T, Hongoh Y. The candidate phylum 'Termite Group 1' of bacteria: phylogenetic diversity, distribution, and endosymbiont members of various gut flagellated protists. FEMS Microbiol Ecol. 2007;60:467-76.
42. Hongoh Y, Sharma VK, Prakash T, Noda S, Taylor TD, Kudo T, et al. Complete genome of the uncultured Termite Group 1 bacteria in a single host protist cell. Proc Natl Acad Sci U S A. 2008;105:5555-60.
43. Kuwahara H, Yuki M, Izawa K, Ohkuma M, Hongoh Y. Genome of '*Ca.* Desulfovibrio trichonymphae', an H_2-oxidizing bacterium in a tripartite symbiotic system within a protist cell in the termite gut. ISME J. 2017;11:766-76.
44. Takeuchi M, Kuwahara H, Murakami T, Takahashi K, Kajitani R, Toyoda A, et al. Parallel reductive genome evolution in *Desulfovibrio* ectosymbionts independently acquired by *Trichonympha* protists in the termite gut. ISME J. 2020;14:2288-301.
45. Hongoh Y, Sharma VK, Prakash T, Noda S, Toh H, Taylor TD, et al. Genome of an endosymbiont coupling N_2 fixation to cellulolysis within protist cells in termite gut. Science. 2008;322:1108-9.
46. Noda S, Iida T, Kitade O, Nakajima H, Kudo T, Ohkuma M. Endosymbiotic *Bacteroidales* bacteria of the flagellated protist *Pseudotrichonympha grassii* in the gut of the termite *Coptotermes formosanus*. Appl Environ Microbiol. 2005;71:8811-7.
47. Noda S, Kitade O, Inoue T, Kawai M, Kanuka M, Hiroshima K, et al. Cospeciation in the triplex symbiosis of termite gut protists (*Pseudotrichonympha* spp.), their hosts, and their bacterial endosymbionts. Mol Ecol. 2007;16:1257-66.
48. Strassert JF, Kohler T, Wienemann TH, Ikeda-Ohtsubo W, Faivre N, Franckenberg S, et al. '*Candidatus* Ancillula trichonymphae', a novel lineage of endosymbiotic *Actinobacteria* in termite gut flagellates of the genus *Trichonympha*. Environ Microbiol. 2012;14:3259-70.
49. Strassert JFH, Mikaelyan A, Woyke T, Brune A. Genome analysis of '*Candidatus* Ancillula trichonymphae', first representative of a deep-branching clade of *Bifidobacteriales*, strengthens evidence for convergent evolution in flagellate endosymbionts. Environ Microbiol Rep. 2016;8:865-73.
50. Noda S, Inoue T, Hongoh Y, Kawai M, Nalepa CA, Vongkaluang C, et al. Identification and characterization of ectosymbionts of distinct lineages in *Bacteroidales* attached to flagellated protists in the gut of termites and a wood-feeding cockroach. Environ Microbiol. 2006;8:11-20.

51. Noda S, Hongoh Y, Sato T, Ohkuma M. Complex coevolutionary history of symbiotic Bacteroidales bacteria of various protists in the gut of termites. BMC Evol Biol. 2009;9:e158.
52. Desai MS, Brune A. *Bacteroidales* ectosymbionts of gut flagellates shape the nitrogen-fixing community in dry-wood termites. ISME J. 2012;6:1302-13.
53. Desai MS, Strassert JF, Meuser K, Hertel H, Ikeda-Ohtsubo W, Radek R, et al. Strict cospeciation of devescovinid flagellates and *Bacteroidales* ectosymbionts in the gut of dry-wood termites (Kalotermitidae). Environ Microbiol. 2010;12:2120-32.
54. Sato T, Kuwahara H, Fujita K, Noda S, Kihara K, Yamada A, et al. Intranuclear verrucomicrobial symbionts and evidence of lateral gene transfer to the host protist in the termite gut. ISME J. 2014;8:1008-19.
55. Strassert JFH, Desai MS, Radek R, Brune A. Identification and localization of the multiple bacterial symbionts of the termite gut flagellate *Joenia annectens*. Microbiology (Reading, Engl.). 2010;156:2068-79.
56. Noda S, Ohkuma M, Yamada A, Hongoh Y, Kudo T. Phylogenetic position and in situ identification of ectosymbiotic spirochetes on protists in the termite gut. Appl Environ Microbiol. 2003;69:625-33.
57. Iida T, Ohkuma M, Ohtoko K, Kudo T. Symbiotic spirochetes in the termite hindgut: phylogenetic identification of ectosymbiotic spirochetes of oxymonad protists. FEMS Microbiol Ecol. 2000;34:17-26.
58. Jumper J, Evans R, Pritzel A, Green T, Figurnov M, Ronneberger O, et al. Highly accurate protein structure prediction with AlphaFold. Nature. 2021;596:583-9.
59. Holm L. Dali server: structural unification of protein families. Nucleic Acids Res. 2022;50:W210-W5.
60. Collins MD, Lawson PA, Willems A, Cordoba JJ, Fernandez-Garayzabal J, Garcia P, et al. The phylogeny of the genus *Clostridium*: proposal of five new genera and eleven new species combinations. Int J Syst Bacteriol. 1994;44:812-26.

Supplementary Tables

Table S1: 16S rRNA genes used in phylogenetic analysis (see Fig. 2 and S2).

accession numbers in SILVA or LTP used in Fig. 2	
AB192174	KP090466
AB299544	KP690938
AB555038	LN612663
AB612693	X66002
AB614861	MH043116
AB614876	MN851263
AB615006	MT875034
AB746684	FJ805840
AB746749	AJ305238
AB821861	KR364749
AF371774	L76600
DQ795962	MN872474
DQ796114	MK761171
DQ796268	LC589980
DQ796766	MT580120
DQ796799	MT905216
EF445283	NR_144727
EU461213	NR_144736
EU463585	LT985454
EU778659	LT897838
EU843933	ON921083
EU845152	LT732642
FJ366876	LT960605
GQ134270	Z49863*
GQ502578	JF750939*
GQ502583	
GQ898015	
GU174104	
HM630202	
HM630229	
HM630241	
JQ184761	
JQ184883	
JQ184923	
JQ185143	
JQ185474	
JQ191019	
JQ617823	
JQ993514	
JQ993520	
JQ993637	
JQ993641	
JQ993642	
KC163071	
KF318227	
KF841696	
KJ399537	
KM251066	

accession numbers in SILVA, LTP, or type strain genome database used in Fig. S2A			
EF529620	EU474489	AB011057	JAOQKF000000000
FR749897	DQ015339	ACXX02000011	JAOQKG000000000
X66002	HM328208	X71856	GCM10018885
MH043116	KM650259	MK935571	LN866995
MN851263	KM650490	AB267266	NR_147398
MT875034	EU459548	FR749966	NR_179526
FJ805840	CCFG01000002	FJ815191	NR_147370
AJ305238	HQ769937	AB793710	NR_144722
KR364749	DQ794293	HE862234	NR_144727
L76600	DQ394630	AEDB02000059	NR_144736
MN872474	DQ796766	X71846	LN881596
MK761171	DQ796799	AY959944	LT631513
LC589980	KT337631	AB186359	LT576393
L09177	KT337629	CP000568	LT598578
AY295777	JQ993514	AB125279	LN870316
AY487928	JQ993641	MK138666	LT558850
ACEC01000059	EU828413	LN868252	LT725660
L76596	EU828406	HG003571	KY777734
AB910742	EU843382	AJ575187	LT985392
AM915269	EU463443	AB910750	LT934440
CP002403	EF096602	GQ900631	Y18180
MH200617	EF097212	AJ310082	LT985454
AJ315980	EF097423	AB221372	LT897838
EU158190	DQ796325	KT630605	GCM10018801
LT576408	EF097263	LC522508	GCM10018815
LT854295	EU469153	LC522510	OL617409
KU999999	HQ780956	LC522513	ON921083
X98011	KT337628	MN055917	LT732642
X97852	EU887962	MN055919	LT960605
KM098109	EU887966	KX146426	CP000448 *
LN846908	EU887963	MT580120	FQWY01000001 *
AJ518869	EU887967	MZ310595	
AJ413954	DQ796114	AJ270469	
KX150462	DQ796268	DQ057476	
ABCA03000037	HQ789326	LC670256	
LC371917	JQ993637	MT905130	
AY136666	GQ132241	MT905132	
AGCK01000014	EU843933	MT905147	
X81125	KX672314	MT905148	
JF750939	KX672322	MT905149	
AP012044	KM251066	MT905206	
MG963288	KM251154	MT905207	
AF167711	DQ015324	MT905208	
Z49863	JQ617823	MT905216	
KP889099	JQ625023	MT905210	
L34618	EU461213	JAOQJZ000000000	
KY978733	HM218859	JAOQKB000000000	
CP001348	DQ394629	JAOQJE000000000	

accession numbers in SILVA or LTP used in Fig. S2B			
AB034072	EF686627	KX672258	JQ993642
AB185796	EU622658	KX672556	KC163071
AB506382	EU622736	LAQZ01000049	KF318227
AB606299	EU828413	LC036223	KF841696

AB622826	FJ880466	LC036224	KJ399537
AB626936	FPLM01005507	LC194924	KM251066
AB627566	FPLO01002175	AB192174	KP090466
AB627625	GQ402108	AB299544	KP690938
AB627689	GQ451304	AB555038	LN612663
AB627717	GQ999966	AB612693	X66002
AB700411	GU907824	AB614861	MH043116
AB746644	GU907825	AB614876	MN851263
AB746645	HM124146	AB615006	MT875034
AB746650	HM124266	AB746684	FJ805840
AB746713	HQ400121	AB746749	AJ305238
AB746717	HQ400259	AB821861	KR364749
AB746718	HQ746235	AF371774	L76600
AB746727	HQ780956	DQ795962	MN872474
AB746748	HQ789326	DQ796114	MK761171
AB746764	HQ805976	DQ796268	LC589980
AB818589	HQ806126	DQ796766	MT580120
AB818596	HQ810098	DQ796799	MT905216
AB821695	HQ814750	EF445283	NR_144727
AB821707	JF766393	EU461213	NR_144736
AB824445	JN653041	EU463585	LT985454
AB930590	JQ084431	EU778659	LT897838
AB969538	JQ085232	EU843933	ON921083
AF332709	JQ248107	EU845152	LT732642
AF371771	JQ607884	FJ366876	LT960605
AJ295664	JQ799169	GQ134270	Z49863 *
AM278898	JQ996700	GQ502578	JF750939 *
AM697405	JX575812	GQ502583	
AY949858	JX575926	GQ898015	
AY949859	KC163015	GU174104	
BCAA01000057	KC163094	HM630202	
CCFG01000002	KC555197	HM630229	
DQ394691	KJ734862	HM630241	
DQ777904	KM244841	JQ184761	
DQ905330	KM244901	JQ184883	
DQ905407	KM250930	JQ184923	
DQ905581	KM454168	JQ185143	
DQ905824	KM978279	JQ185474	
EF071387	KT337628	JQ191019	
EF400741	KT337630	JQ617823	
EF404262	KT337631	JQ993514	
EF405002	KT935669	JQ993520	
EF405280	KT952843	JQ993637	
EF686593	KX672228	JQ993641	

* outgroup sequence

Table S2: PCR primers designed in this study.

name	sequence (5' – 3')	target
RsTa-C01_Fwd	tagccggactgagaggttg	16S rRNA gene of RsTa-C01
RsTa-C01_Rev	catctcacgacacgagctg	16S rRNA gene of RsTa-C01
CfP3-15_Fwd	tgagtaaagtagaggcgaacg	16S rRNA gene of CfP3-15
CfP3-15_Rev	aggccatctttgagggattag	16S rRNA gene of CfP3-15
NkDv07_Fwd	aacacatgcaagtcgaacg	16S rRNA gene of NkDv07
NkDv07_Rev	ccttgcggttaggctatcg	16S rRNA gene of NkDv07
RsTa-C01_tlcA_189F	aatacgctgtgatgggagcc	*tlcA* of RsTa-C01
RsTa-C01_tlcA_493R	gatgagactgcaatcgtacc	*tlcA* of RsTa-C01
CfP3-15_tlcA-1_862F	atgcttccggtgtgttgtgg	*tlcA* (*Clostridia* ATP/ADP translocase 1) of CfP3-15
CfP3-15_tlcA-1_1227R	acaatacgttgccccttgc	*tlcA* (*Clostridia* ATP/ADP translocase 1) of CfP3-15
CfP3-15_tlcA-2_558F	tccgttggtagttgccttgg	*tlcA* (*Clostridia* ATP/ADP translocase 2) of CfP3-15
CfP3-15_tlcA-2_933R	ttcaacgcccgaatacctcg	*tlcA* (*Clostridia* ATP/ADP translocase 2) of CfP3-15
CfP3-15_0049_210F	agtcagtgaaggcgaagacg	CfP3-15_0049
CfP3-15_0049_569R	tctgcaccaacagtcggaac	CfP3-15_0049
CfP3-15_0165_156F	tgtgggtgacgttcatggag	CfP3-15_0165
CfP3-15_0165_474R	atgaatttccgcagcagcac	CfP3-15_0165
CfP3-15_0552_289F	gagggtttctgccgatatgc	CfP3-15_0552
CfP3-15_0552_541R	ttgcgtaatggccgtcatc	CfP3-15_0552
CfP3-15_0627_105F	tgcattgttggcagtttcgg	CfP3-15_0627
CfP3-15_0627_411R	gggtatagaaagcaacgccg	CfP3-15_0627
CfP3-15_0790_107F	tcggtgatgaaggatcggg	CfP3-15_0790
CfP3-15_0790_479R	acttcgtcccttagagtcgc	CfP3-15_0790
CfP3-15_0852_325F	gttggtggtttgggtttggc	CfP3-15_0852
CfP3-15_0852_648R	ttcttgtcccgtggtactcc	CfP3-15_0852
RsTa-C01_0144_48F	gtttgcacaagtttcggctc	RsTa-C01_0144
RsTa-C01_0144_392R	tcaggtgcaatttcacaagaagc	RsTa-C01_0144
RsTa-C01_0431_85F	atgagcggtaacaagccaaatg	RsTa-C01_0431

RsTa-C01_0431_453R	ttccaagaaccaggctaaac	RsTa-C01_0431
RsTa-C01_0774_124F	attgagacacatggcactgg	RsTa-C01_0774
RsTa-C01_0774_468R	ttggtattcgtagcaaaggg	RsTa-C01_0774
RsTa-C01_0844_930F	acaagtgttgccgcaaagtc	RsTa-C01_0844
RsTa-C01_0844_1249R	agcggcatgctactatcatc	RsTa-C01_0844

Table S3: MAGs used in phylogenetic and other comparative analyses (see Figs. 5, S13 and S14).

accession number or UHGG ID	accession number or UHGG ID	accession number or UHGG ID	accession number or UHGG ID
GCA_000432435.1	GCA_017428625.1	GCA_017631705.1	MGYG000003581
GCA_000432995.1	GCA_017429345.1	GCA_017631795.1	MGYG000003591
GCA_000433975.1	GCA_017431505.1	GCA_017631955.1	MGYG000003628
GCA_000434915.1	GCA_017431755.1	GCA_017634155.1	MGYG000003629
GCA_000435275.1	GCA_017432165.1	GCA_017648315.1	MGYG000003641
GCA_000435335.1	GCA_017432485.1	GCA_017650655.1	MGYG000003752
GCA_000577335.1	GCA_017432865.1	GCA_017935265.1	MGYG000003768
GCA_000752215.1	GCA_017433225.1	GCA_017935325.1	MGYG000003809
GCA_001261775.1	GCA_017433905.1	GCA_017936365.1	MGYG000003841
GCA_001512765.1	GCA_017434895.1	GCA_017936615.1	MGYG000003842
GCA_001513045.1	GCA_017434985.1	GCA_017938345.1	MGYG000003863
GCA_001513365.1	GCA_017435305.1	GCA_017938985.1	MGYG000003872
GCA_002069015.1	GCA_017435385.1	GCA_017939855.1	MGYG000003889
GCA_002069695.1	GCA_017435665.1	GCA_017939885.1	MGYG000003947
GCA_002119605.1	GCA_017435765.1	GCA_017942105.1	MGYG000003955
GCA_002160025.1	GCA_017435865.1	GCA_017942115.1	MGYG000004027
GCA_002201475.1	GCA_017436085.1	GCA_017959985.1	MGYG000004032
GCA_002293315.1	GCA_017436225.1	GCA_017963405.1	MGYG000004049
GCA_002297415.1	GCA_017436255.1	GCA_900048895.1	MGYG000004075
GCA_002305575.1	GCA_017436285.1	GCA_900100595.1	MGYG000004076
GCA_002308635.1	GCA_017436375.1	GCA_900101355.1	MGYG000004106
GCA_002309055.1	GCA_017436725.1	GCA_900176635.1	MGYG000004115
GCA_002309275.1	GCA_017436795.1	GCA_900184925.1	MGYG000004116
GCA_002314305.1	GCA_017436915.1	GCA_900197595.1	MGYG000004129
GCA_002314645.1	GCA_017437005.1	GCA_900240385.1	MGYG000004131
GCA_002315505.1	GCA_017437165.1	GCA_900291955.1	MGYG000004146
GCA_002315685.1	GCA_017438225.1	GCA_900314355.1	MGYG000004159
GCA_002315765.1	GCA_017438505.1	GCA_900314795.1	MGYG000004229
GCA_002315935.1	GCA_017438585.1	GCA_900315875.1	MGYG000004265
GCA_002316805.1	GCA_017438845.1	GCA_900315985.1	MGYG000004279
GCA_002320555.1	GCA_017439545.1	GCA_900316025.1	MGYG000004292
GCA_002329225.1	GCA_017439585.1	GCA_900316345.1	MGYG000004322
GCA_002329285.1	GCA_017439765.1	GCA_900316385.1	MGYG000004334
GCA_002329365.1	GCA_017439845.1	GCA_900316925.1	MGYG000004413
GCA_002338255.1	GCA_017439875.1	GCA_900317765.1	MGYG000004437
GCA_002350765.1	GCA_017440755.1	GCA_900317795.1	MGYG000004470
GCA_002359465.1	GCA_017440765.1	GCA_900318905.1	MGYG000004496
GCA_002361875.1	GCA_017440825.1	GCA_900320995.1	MGYG000004543
GCA_002361935.1	GCA_017440965.1	GCA_900321195.1	MGYG000004555
GCA_002363095.1	GCA_017441385.1	GCA_900321595.1	MGYG000004560
GCA_002371985.1	GCA_017442225.1	GCA_900539165.1	MGYG000004616
GCA_002372375.1	GCA_017442265.1	GCA_900539325.1	MGYG000004643
GCA_002378845.1	GCA_017442345.1	GCA_900539425.1	MGYG000004723
GCA_002391625.1	GCA_017442445.1	GCA_900540085.1	MGYG000004727
GCA_002397355.1	GCA_017443805.1	GCA_900540275.1	MGYG000004807
GCA_002399225.1	GCA_017445905.1	GCA_900540295.1	MGYG000004815
GCA_002399445.1	GCA_017446095.1	GCA_900541565.1	MGYG000004850
GCA_002407645.1	GCA_017448285.1	GCA_900542375.1	MGYG000004892
GCA_002407675.1	GCA_017449935.1	GCA_900542575.1	MGYG000290008
GCA_002409375.1	GCA_017450005.1	GCA_900542875.1	MGYG000290033
GCA_002409585.1	GCA_017450135.1	GCA_900543095.1	MGYG000290055
GCA_002409675.1	GCA_017450185.1	GCA_900543215.1	MGYG000290071
GCA_002410755.1	GCA_017450645.1	GCA_900544375.1	MGYG000290075
GCA_002411365.1	GCA_017451425.1	GCA_900545655.1	MGYG000290084
GCA_002424295.1	GCA_017452785.1	GCA_900546265.1	MGYG000290120

GCA_002437245.1	GCA_017453685.1	GCA_900546785.1	MGYG000290125
GCA_002437905.1	GCA_017454485.1	GCA_900547095.1	MGYG000290153
GCA_002438505.1	GCA_017454815.1	GCA_900547105.1	MGYG000290169
GCA_002438685.1	GCA_017455295.1	GCA_900547305.1	MGYG000290178
GCA_002449395.1	GCA_017456015.1	GCA_900547445.1	MGYG000290186
GCA_002451715.1	GCA_017456445.1	GCA_900547915.1	MGYG000290211
GCA_002451755.1	GCA_017456845.1	GCA_900548305.1	MGYG000290227
GCA_002451855.1	GCA_017457155.1	GCA_900549675.1	MGYG000290295
GCA_002490425.1	GCA_017457955.1	GCA_900549755.1	MGYG000290335
GCA_002491825.1	GCA_017458105.1	GCA_900549825.1	MGYG000290439
GCA_002493005.1	GCA_017458845.1	GCA_900551425.1	MGYG000290441
GCA_002493325.1	GCA_017458945.1	GCA_900552265.1	MGYG000290485
GCA_002493595.1	GCA_017458975.1	GCA_900553525.1	MGYG000290552
GCA_002493755.1	GCA_017460565.1	GCA_900554165.1	MGYG000290559
GCA_002494125.1	GCA_017461005.1	GCA_900554335.1	MGYG000290567
GCA_002834225.1	GCA_017461235.1	GCA_900554775.1	MGYG000290582
GCA_002834235.1	GCA_017461625.1	GCA_900555015.1	MGYG000290690
GCA_003268275.1	GCA_017462405.1	GCA_900555805.1	MGYG000290832
GCA_003438075.1	GCA_017463345.1	GCA_900754145.1	MGYG000290859
GCA_003499325.1	GCA_017463985.1	GCA_900761545.1	MGYG000290913
GCA_003505905.1	GCA_017464405.1	GCA_900764665.1	MGYG000290943
GCA_003514385.1	GCA_017465085.1	GCA_900766895.1	MGYG000291000
GCA_003517915.1	GCA_017465285.1	GCA_900767015.1	MGYG000291022
GCA_003520555.1	GCA_017465465.1	GCA_900768035.1	MGYG000291055
GCA_003521625.1	GCA_017465505.1	GCA_900769375.1	MGYG000291062
GCA_003538135.1	GCA_017465825.1	GCA_900769535.1	MGYG000291101
GCA_003612555.1	GCA_017466205.1	GCA_900772705.1	MGYG000291106
GCA_004103755.1	GCA_017466365.1	GCA_902761345.1	MGYG000291180
GCA_004555265.1	GCA_017466905.1	GCA_902761375.1	MGYG000291183
GCA_004555625.1	GCA_017468425.1	GCA_902762615.1	MGYG000291203
GCA_004560045.1	GCA_017470025.1	GCA_902763855.1	MGYG000291207
GCA_004560275.1	GCA_017471345.1	GCA_902765855.1	MGYG000291209
GCA_004768785.1	GCA_017471605.1	GCA_902766775.1	MGYG000291284
GCA_005601135.1	GCA_017472575.1	GCA_902766885.1	MGYG000291342
GCA_009746625.1	GCA_017473165.1	GCA_902767155.1	MGYG000291369
GCA_009774715.1	GCA_017473435.1	GCA_902767285.1	MGYG000291396
GCA_009774735.1	GCA_017473555.1	GCA_902767325.1	MGYG000291400
GCA_009777055.1	GCA_017473775.1	GCA_902768825.1	MGYG000291443
GCA_009777895.1	GCA_017473925.1	GCA_902771755.1	MGYG000291527
GCA_009779455.1	GCA_017474045.1	GCA_902773045.1	MGYG000291606
GCA_009779495.1	GCA_017474125.1	GCA_902773265.1	MGYG000291625
GCA_009783605.1	GCA_017474205.1	GCA_902773635.1	MGYG000291638
GCA_009784075.1	GCA_017475765.1	GCA_902774325.1	MGYG000291646
GCA_009785335.1	GCA_017476375.1	GCA_902777275.1	MGYG000291751
GCA_009785985.1	GCA_017477885.1	GCA_902785365.1	MGYG000291753
GCA_009786275.1	GCA_017478005.1	GCA_902785425.1	MGYG000291756
GCA_009786735.1	GCA_017480115.1	GCA_902785555.1	MGYG000291761
GCA_009787315.1	GCA_017480265.1	GCA_902786345.1	MGYG000291777
GCA_009917525.1	GCA_017481085.1	GCA_902787145.1	MGYG000291785
GCA_009930155.1	GCA_017481115.1	GCA_902790475.1	MGYG000291791
GCA_009936035.1	GCA_017481395.1	GCA_902790985.1	MGYG000291793
GCA_012511005.1	GCA_017481575.1	GCA_902796915.1	MGYG000291887
GCA_012511485.1	GCA_017481865.1	GCA_902798365.1	MGYG000291916
GCA_012516295.1	GCA_017481885.1	GCA_902798605.1	MGYG000291946
GCA_012517675.1	GCA_017482035.1	GCA_902798755.1	MGYG000291949
GCA_012519975.1	GCA_017483265.1	GCA_902800005.1	MGYG000291967
GCA_012520115.1	GCA_017500965.1	GCA_902800065.1	MGYG000292005

GCA_012520505.1	GCA_017501865.1	GCA_902801245.1	MGYG000292015
GCA_012522615.1	GCA_017502005.1	GCA_902802795.1	MGYG000292022
GCA_012523185.1	GCA_017502085.1	GCA_902802985.1	MGYG000292086
GCA_012524025.1	GCA_017502405.1	GCA_902803805.1	MGYG000292095
GCA_012728445.1	GCA_017502425.1	GCA_902809935.1	MGYG000292159
GCA_012839185.1	GCA_017503135.1	GCA_902810075.1	MGYG000292186
GCA_012839345.1	GCA_017503625.1	MGYG000000069	MGYG000292239
GCA_012839705.1	GCA_017503705.1	MGYG000000129	MGYG000292281
GCA_012840445.1	GCA_017503785.1	MGYG000000295	MGYG000292320
GCA_014384705.1	GCA_017504645.1	MGYG000000388	MGYG000292352
GCA_014465955.1	GCA_017504765.1	MGYG000000390	MGYG000292489
GCA_017379935.1	GCA_017507805.1	MGYG000000395	MGYG000292574
GCA_017380115.1	GCA_017511525.1	MGYG000000443	MGYG000292617
GCA_017380355.1	GCA_017512465.1	MGYG000000453	MGYG000292623
GCA_017380575.1	GCA_017513165.1	MGYG000000456	MGYG000292633
GCA_017380615.1	GCA_017513525.1	MGYG000000505	MGYG000292754
GCA_017381035.1	GCA_017514345.1	MGYG000000607	MGYG000292779
GCA_017381515.1	GCA_017515685.1	MGYG000000646	MGYG000292782
GCA_017382015.1	GCA_017516315.1	MGYG000000669	MGYG000292814
GCA_017382915.1	GCA_017516445.1	MGYG000000704	MGYG000292850
GCA_017383605.1	GCA_017516485.1	MGYG000000726	MGYG000292854
GCA_017383985.1	GCA_017516965.1	MGYG000000752	MGYG000292917
GCA_017384945.1	GCA_017517205.1	MGYG000000924	MGYG000292997
GCA_017385305.1	GCA_017517525.1	MGYG000000932	MGYG000293109
GCA_017386515.1	GCA_017517665.1	MGYG000000937	MGYG000293123
GCA_017387385.1	GCA_017517865.1	MGYG000001029	MGYG000293161
GCA_017387475.1	GCA_017518415.1	MGYG000001069	MGYG000293167
GCA_017389925.1	GCA_017519685.1	MGYG000001081	MGYG000293196
GCA_017390725.1	GCA_017520635.1	MGYG000001082	MGYG000293206
GCA_017390825.1	GCA_017521125.1	MGYG000001116	MGYG000293234
GCA_017391425.1	GCA_017521925.1	MGYG000001125	MGYG000293289
GCA_017391765.1	GCA_017522205.1	MGYG000001169	MGYG000293318
GCA_017391805.1	GCA_017522225.1	MGYG000001212	MGYG000293344
GCA_017391865.1	GCA_017522455.1	MGYG000001244	MGYG000293370
GCA_017393565.1	GCA_017523865.1	MGYG000001569	MGYG000293383
GCA_017396175.1	GCA_017524165.1	MGYG000001572	MGYG000293642
GCA_017396345.1	GCA_017528095.1	MGYG000001654	MGYG000293699
GCA_017397065.1	GCA_017531385.1	MGYG000001685	MGYG000293767
GCA_017397105.1	GCA_017536525.1	MGYG000001737	MGYG000293769
GCA_017397655.1	GCA_017537245.1	MGYG000001749	MGYG000293853
GCA_017397845.1	GCA_017537365.1	MGYG000001760	MGYG000293887
GCA_017398425.1	GCA_017539305.1	MGYG000001801	MGYG000293910
GCA_017398485.1	GCA_017543285.1	MGYG000001837	MGYG000294093
GCA_017399065.1	GCA_017546535.1	MGYG000001860	MGYG000294096
GCA_017399405.1	GCA_017546685.1	MGYG000001956	MGYG000294115
GCA_017400255.1	GCA_017547465.1	MGYG000002001	MGYG000294117
GCA_017401365.1	GCA_017547725.1	MGYG000002019	MGYG000294154
GCA_017402845.1	GCA_017547865.1	MGYG000002031	MGYG000294286
GCA_017403065.1	GCA_017548025.1	MGYG000002038	MGYG000294288
GCA_017403265.1	GCA_017548045.1	MGYG000002093	MGYG000294362
GCA_017403985.1	GCA_017548305.1	MGYG000002135	MGYG000294373
GCA_017404145.1	GCA_017549105.1	MGYG000002159	MGYG000294398
GCA_017404665.1	GCA_017549285.1	MGYG000002192	MGYG000294482
GCA_017405065.1	GCA_017549565.1	MGYG000002196	MGYG000294524
GCA_017406805.1	GCA_017550105.1	MGYG000002228	MGYG000294526
GCA_017407645.1	GCA_017550625.1	MGYG000002232	MGYG000294547
GCA_017407665.1	GCA_017551295.1	MGYG000002233	MGYG000294561

GCA_017407705.1	GCA_017552225.1	MGYG000002258	MGYG000294654
GCA_017407755.1	GCA_017552785.1	MGYG000002260	MGYG000294693
GCA_017407995.1	GCA_017552845.1	MGYG000002262	MGYG000294701
GCA_017408455.1	GCA_017554125.1	MGYG000002553	MGYG000294705
GCA_017408945.1	GCA_017554405.1	MGYG000002570	MGYG000294754
GCA_017409265.1	GCA_017555765.1	MGYG000002694	MGYG000294801
GCA_017409475.1	GCA_017556785.1	MGYG000002702	MGYG000294827
GCA_017410195.1	GCA_017557825.1	MGYG000002705	MGYG000294848
GCA_017410265.1	GCA_017559305.1	MGYG000002805	MGYG000294892
GCA_017410545.1	GCA_017560255.1	MGYG000002857	MGYG000294994
GCA_017410605.1	GCA_017560725.1	MGYG000002934	MGYG000294995
GCA_017411385.1	GCA_017613735.1	MGYG000002984	MGYG000295003
GCA_017411425.1	GCA_017614835.1	MGYG000003004	MGYG000295013
GCA_017411645.1	GCA_017615405.1	MGYG000003059	MGYG000295030
GCA_017411835.1	GCA_017618135.1	MGYG000003156	MGYG000295033
GCA_017414705.1	GCA_017618535.1	MGYG000003160	MGYG000295123
GCA_017414725.1	GCA_017621885.1	MGYG000003161	MGYG000295151
GCA_017414965.1	GCA_017622415.1	MGYG000003176	MGYG000295160
GCA_017415025.1	GCA_017623315.1	MGYG000003211	MGYG000295212
GCA_017415515.1	GCA_017624355.1	MGYG000003236	MGYG000295220
GCA_017416065.1	GCA_017624675.1	MGYG000003243	MGYG000295236
GCA_017416345.1	GCA_017624735.1	MGYG000003278	MGYG000295277
GCA_017416765.1	GCA_017625095.1	MGYG000003296	MGYG000295278
GCA_017418545.1	GCA_017625435.1	MGYG000003412	MGYG000295283
GCA_017419505.1	GCA_017625835.1	MGYG000003428	MGYG000295359
GCA_017422485.1	GCA_017626295.1	MGYG000003437	MGYG000295423
GCA_017423825.1	GCA_017626835.1	MGYG000003453	MGYG000295437
GCA_017425455.1	GCA_017629015.1	MGYG000003473	MGYG000295480
GCA_017425745.1	GCA_017629275.1	MGYG000003509	MGYG000295483
GCA_017427315.1	GCA_017630985.1	MGYG000003524	MGYG000295535
GCA_017428085.1	GCA_017631355.1	MGYG000003557	MGYG000295546
GCA_017428525.1	GCA_017631515.1	MGYG000003576	GCA_000014725.1*
			GCA_900129805.1*

* outgroup sequence

Table S4. ATP/ADP translocase genes used in phylogenetic analysis (see Fig. 6).

UniProtKB ID	UniProtKB ID	UniProtKB ID	UniProtKB ID	UniProtKB ID	ID*
R6GHN2	A0A2A4YG67	A0A3B7D973	A0A431IJ07	A0A0G0BB48	AJ582018
B0RZB7	F8L6N5	H8K3Y2	A0A2N2F6F9	A0A7V1HE39	AJ582019
Q1MSG2	A0A2H0VPK6	A0A6H3RXP3	A0A523TUZ0	A0A431IIR0	AY120885
A0A0K8MFA8	A0A6M2A234	A0A126E8I3	A0A0F9ZA13	A0A0D2K661	AP002546
A0A4Q7DJM4	A0A6M1YH83	A0A0F3RDJ1	A0A0G0BWV0	A0A1F4QN25	NC_002491
A0A1F8JKC1	A0A2H9SU81	A0A0F3R7J0	A0A1F4PIB3	A0A345ZCK4	Ntt2_Cab
A0A7W0FTB3	A0A0C1C570	H8K922	A0A1F4QN61	A0A1F4PQA9	AJ010587
A0A1M3BBW7	A0A7W1RFY4	A8GLZ6	A0A523RJZ1	A0A345ZCK5	AE002346
A0A0U5JD23	D6YSH7	A0A0C2QYA1	A0A3D4XWN9	A0A0G0V8C0	AJ582023
A0A2H0VTK1	A0A2E3LYY9	A0A7U0FXV9	A0A1V5QV55	A0A0G0V828	AJ582020
A0A3D2GUK3	A0A7W1JM80	A0A510G6D1	A0A0G1B5V2	V6DIH2	AJ582022
A0A077AUX9	A0A090CYR0	A8EXC0	A0A355KQK0	A0A7W1KD37	AJ582021
A0A7W1M910	A0A1G3Z804	A0A0F3QE15	A0A0G1B3B1	A0A0G0B9Z5	NM_106679
A0A1M3GVC4	A0A3D2HI91	A0A1C3EWJ7	A0A355KRG2	A0A0G0JXR7	AC013453
A0A358T439	A0A1J4XF64	A0A0F3QKS4	A0A355KQN5	A0A0G0I272	Y10821
A0A1W6N3J5	A0A1Q3P7P4	A0A261DBG2	A0A349U2F8	A0A0G0I2Y5	AY098893
A0A355VTN1	A0A3S8T1B0	A0A4Q3DK00	A0A0G0IDH3	A0A7T9BFC6	AP003234
A0A355VUV8	A0A5B8XEK2	A0A2A5B6J7	A0A0G0J7G9	A0A0F9Y0G0	AJ251356
A0A7W2B8S5	A0A6J5JXQ0	A0A3S0J7L7	A0A7S1N3C9		AE008630
A0A1M3IUX2	A0A7V9ZM35	Q842J1	A0A0G0JZ48		EAA26488
A0A1M3IQK7	A0A258XDK4	A0A0F4VNY1	A0A2E3UXJ6		AJ507306
A0A0W0YL02	A0A1M3IT18	A0A1V2N7Z3	A0A7T8YHP0		AJ507305
A0A7W1REL4	A0A2M7T3X8	A0A424FMM2	A0A7T8YHT3		AJ235271_1
A0A4S1MAI1	A0A2D9KQJ9	A0A2T4VXV6	A0A7T9BEV6		AJ507307
A0A1F8JIF2	A0A2N6AEX9	C6XHQ3	A0A0F9XX69		AE008663
A0A6M1ZTJ2	A0A5C0UI02	D0V1N0	A0A0G0AQJ7		EAA26151
A0A6M1YG67	A0A2R8F168	F7XV04	A0A0G0AA79		AJ507312
A0A212KR42	A0A2U3RFE7	A0A2A5BCR7	A0A0D2I1H5		AJ235273
A0A7H5P4T7	A0A0F3NT69	A0A0H3AUS9	V6DI64		AJ507311
A0A0H2X0K7	A0A2U3QZ94	A0A7W1MBQ0	A0A1F4QNL7		AJ507313
O84068	A0A2U3REE8	A0A258XCV7	A0A2E6XWN8		AJ507309
A0A0H3MGN2	A0A2U3RPD8	A0A258X8X1	A0A2E6XVP0		AJ235272
A0A069ZTL9	A5CCY4	A0A258XDT1	A0A345ZCC7		AJ507310
S7KMH9	A0A5P2GM32	A0A2H0VVI2	A0A2A4WV73		AJ507308
A0A3B0PRX3	A0A2U3R2Y2	A0A3B8U8W6	A0A2A4WU23		AE008626
Q823H5	A0A0F3RK25	A0A6M1ZTP4	A0A3D4XV95		EAA25317
A0A656IUS2	A0A0F3MIA5	K1Y0X6	A0A0F9XZ47		AE008614
S7IUE0	D5AVX6	A0A6M2AEZ9	A0A0G0IC44		EAA25449
A0A0E2IZV9	Q68XS7	A0A0H5DUE5	A0A0G0J7L7		AJ507303
Q5L657	Q83W30	A0A090D030	A0A0G0DCP8		AJ507302
A0A5C1MW90	C4YY86	A0A1F8JLG4	A0A7S4MBI5		AJ235271
A0A173DZV0	A0A161QL04	A0A0G0Y769	A0A7S0IL69		AJ507304
W8JLD3	A0A0H3AVQ4	A0A0G0V565	A0A7S4B8E2		AJ582017
Q254E3	C4K173	A0A0G0Y710	A0A7S4EWG1		NC_003235
V8TP30	H6PVW1	A0A523U2E7	A0A7S4B8I1		NC_003236_2
A0A0F7WSG7	Q7PAI8	V6DFH5	A0A7S3TPR1		NC_003236_3
A0A0F7X0C7	A0A0F3QLL0	A0A7W1KD24	A0A7S3XCL4		NC_003236_1
A0A2R8FAW5	Q92JI6	A0A7W1KD99	A0A7S0LVZ6		
A0A1A9HYH4	H8LP27	A0A0D2JKG0	A0A7T8YHP5		
A0A2A4WVV1	C3PM91	A0A0F9YPF6	A0A0G0KIK2		
A0A3B8U7H6	H8KBF8	A0A7V1MPS5	A0A0G0GNG3		
A0A6G4ZQ23	Q83W40	A0A7V1HEP3	A0A0F9XZR0		
A0A6M1YYA5	A8F0G0	A0A0F9YC01	A0A0G0KKH5		
A0A317JCP9	H6QKS7	K1XSV0	A0A7T9BE65		
A0A486XJX4	A0A0S2CAX6	A0A7T9H7B6	A0A0F9YAB8		

* Sequence IDs of the Schmitz-Esser et al. (2004) dataset

Table S5. Eukaryotic-/Eukaryotic-like genes used in phylogenetic analyses (see Figs. 4 and S9).

UniProtKB ID used in Fig. 4A	UniProtKB ID used in Fig. 4B *	UniProtKB ID used in Fig. S7A *	UniProtKB ID used in Fig. S7B *	UniProtKB ID used in Fig. S7C *
A2EJW2	A0A0F9MGS0	A0A061H4X5	A0A0F7FJ36	A0A1D2NJL0
A0A6U1Z744	X0S206	A0A8H7JWK5	A0A429GSS9	A0A813DRP3
A0A6U2ELB9	A0A7K4GPA7	A0A8H7IUB6	A0A3R9RI95	A0A812IE99
C1E7N3	A0A524EX74	A0A163GEM8	A1RX13	A0A812JE06
A0A6U0NFR7	A0A5C9E0T7	A0A6A5RGB5	A0A1J4JF35	A0A812TX60
A0A6U3GEW5	A0A5C9ERY9	A0A6A6ZV80	A0A1J4KDX1	A0A812K2K7
A0A699YPG0	A0A8H4D775	A0A8K0R389	A0A1J4K7N4	A0A813AUC2
A0A6U1H2R9	A0A2H0ZHW0	A0A6A5QU68	TRINITY_DN8548_c0_g1_i1.p1	A0A1Q9CR86
A0A6V1YRM0	A0A0L0NZ33	A0A178AMZ5	A0A1J4KD15	A0A7S1LKL5
A0A6U2EHJ1	A0A510P9E2	A0A4Q4S4B3	A0A1J4KRZ2	A0A7S2IRI7
A0A0D2LTI7	A0A890D766	A0A6A5JYC4	A2DXF5	A0A7J6L878
A8J4U2	A0A8H7GXT7	M2U9S4	A0A1J4K1A2	A0A7J6LHD6
D8UDK2	Q6BZD6	M2SNR4	A0A1J4KUG9	C5L3R7
A0A0K9RC48	C4YEB1	A0A8H7BFT7	A0A1Y1S6A6	Q3SDK0
A0A446LWG6	A0A2N1J7A6	A0A6A5UZE4	L2GPN7	Q3SDM6
A0A446LWE5	G0R2Q5	A0A2V1DYD2	I3EPT9	Q3SDJ6
A0A446KLF2	J9EG22	A0A6A5U2U5	A0A177EMV5	I7MHF3
A0A446KLA1	A0A1I9G785	A0A6A6UYI8	Q22Z33	J9FS83
A0A5J9TXC9	A0A1I8FUI2	A0A6A6W8B4	M7X1F0	A0A1J5WYR8
A0A2G5E2A4	A0A1I8I979	A0A0D2AI17	B0EHJ8	A0A7S2XY36
A0A2G5E294	A0A336KHI3	A0A3N4LBX1	K2GBZ6	A0A7M5WXS5
A0A2G5E279	A0A7K5SL79	A0A3N4JA11	A0A0N4ZVF7	A0A0G4FIG9
A0A5D3DBB3	A0A7K7MQT5	A0A5J5FBU2	A0A0N5BX46	A0A8J2F5M1
A0A5A7UIP3	A0A0L0FS10	A0A3N4IQI6	A0A0K0F9K4	A0A8J1ZLN5
A0A0K9P1Q3	A0A7S3PVA0	A0A8H3ENN8	A0A090LGS6	Q3SDK3
A0A067GRF5	E1B4V3	A0A8H3I3Z6	A0A8D8RFF8	Q3SDN0
A0A6N2LJI4	A0A0R3WE28	A0A8H6FY27	A0A8D9BZJ7	Q3SD33
A0A6N2LJG2	A0A7S2FKN1	W9CLP2	A0A8D8RJQ9	Q3SCZ8
A0A6N2LLJ9	A0A5B8MUA6	A0A8H2ZN92	A0A8D9DSE8	G0QZL5
A0A371GFU8	A0A177EFK8	A0A061HJ54	A0A8D9C1K6	Q231B4
A0A0L9TV98	I3EH04	N1JDN0	A0A1B6JRI2	A0A8B6XT86
A0A7J9CP80	K2GS41	A0A8H7X6D0	A0A1B6LQE9	A0A7S4IGM2
A0A5J5AT40	Q4QT07	A0A2V1CHG3	A0A0A9XGR4	A0A4D9C553
A5B7L8	B0EQW5	A0A370TYT6	A0A146M8W9	A0A819J260
A0A7N0VJ53	Q5EGP4	A0A559M7G0	A0A023FBL8	A0A816VTI4
G3QAS5	Q3SDK3	A0A8H8S098	A0A812B110	A0A819MPL4
A0A4Z2AY33	Q3SDN0	A0A4Q4SX16	A0A812AZZ3	A0A816PWM8
A0A7J8JP11	A0A8J2F5M1	A0A4Q4VKJ9	A0A1W0WS83	A0A819BVD7
A0A7J8JP19	A0A1J4JVC5	A0A4V1XFC0	A0A8B7YH20	A0A818N9U5
A0A151P2S4	TRINITY_DN7382_c0_g1_i1.p1	A0A4V1XEQ1	A0A8B8CKK3	A0A821P7P9
C3YDA6	A0A852KM46	A0A4Q4ZRQ1	A0A8B8CGL8	A0A815S408
A0A1S3HH50	A0A7K7LJJ0	A0A4Q4WVV0	A0A6J8AYA3	A0A818TY56
A0A6J8EBK4	A0A7K9USC6	A0A4Q4ZPE7	A0A8B6F6T3	A0A821C8F7
A0A6J8E7S2	A0A8C4Q5Z8	A0A1Y2WCP9	A0A076FH86	A0A815TV65
R7V8P8	A0A1J4J649	W3X4Y5	K0KXS0	A0A814XKZ4
A0A6F9DUG7	A2ESK3	A0A1Y2XGL1	A0A1E3P3J0	A0A814QLZ4
A4S845	A0A7S3CVP5	A0A1Y2TZ92	A0A061AVJ6	A0A815JDL7
L1IXS2	T1JME3	A0A1Y2EE74	A0A830I1H1	A0A820TZQ3
A0A6U4DLJ2	E9G2W8	A0A1W2TMU9	Q5D6W2	A0A7S3YE35
D2VZY0	A0A8J2WG29	A0A4Z0Z3W2	A2DJK6	A0A7S3YE77
A0A2V2V2B7	J9I1W7	E3Q2P6	A0A1J4J4M1	A0A3S1BD86
V5BIE1	S7W8I7	A0A066XFF5	A2EN26	L7K0I4
A0A7J6Y812	A2FIJ0	A0A1S1VTF1	A2DHX2	L2GVC0
A0A2V2VCT5	A0A820ZVC4	A0A8H6JPY0	A0A1S3D0W5	A0A671LEH6
A0A2V2X2H9	A0A820Y630	A0A4U6XBJ2	A0A7S0HB51	A0A672RYS7
A0A3R7PIC2	A0A818ZAY2	A0A5Q4BX13	Q98RX2	A0A6P3WCN0
A0A061IZA1	A0A814III5	N4VJD2	A0A654GLI6	A0A3B4DW63
A0A422NP63	A0A814KCR9	A0A2T2ZT58	A0A7M3QA85	A0A5N5KZE5
A0A1X0NNW0	A0A251U4W4	A0A5N5MTR3	A0A196SLS2	A0A4W4EQZ8

Q385X0
A0A1G4ID09
A0A3L6KRT6
D0A6U2
G0V079
G0UAY8
S9TWK5
S9VC96
A0A7G2CKC4
L0AV63
A0A131XVM0
A0A6P6XR97
I7MKZ2
A0A1R2C869
Q22TX5
A0D4B0
A0D283
A0A1J4KTU1
A0A7E4ZT81
A0CGY4
A2FJN1
A2DVU2
A2FCN9
A0A146K3G2
B3RJY9
A0A6T2AG77
A0A151ZH60
A0A6T6W9C0
A0A6V5V221
A0A6U8BAU6
A0A6T1BLD3
A0A6V1QD79
A0A6V3D4S8
A0A6U4IVP2
T0PWX7
A0A6T6JJ43
A0A6P6RQX7
A0A1J4KIC4
A2DY61

A0A2H4SKE0
X6MGW6
A0A7K4GX73
A0A850M276
A0A1J4K2N7
A0A5B9DE64
A0A5B9DFX9
A0A497QHU6
A0A842QXE0
A0A524F9Y6
A0A7K4GLD5
A0A524D9Y9
A0A5C9DTX2
A0A5C9ERY4
A0A842T0B9
A0A524C8J4
A0A524FK55
X0ZEA4
A0A5C9DWI3
A0A7K4H0C6
A0A532TI34
A0A523W0B6

A0A5N5NSZ1
A0A086T1H5
A0A0F7ZQD3
A0A167A5G7
A0A7U3Q103
A0A8K0J7J9
M1W7M1
A0A063CBP1
A0A179HZ11
A0A367L240
A0A2C5Z555
A0A544ZV83
A0A0D2DR91
A0A0D1YEH3
A0A177FHB8
N1Q0R6
A0A6A6C2K9
G8C275
A0A0C7N6G5
A0A1G4JTJ0
Q6CTC6
A0A5P2U6P3
A0A0A8LB19
W0THH7
A0A8F2RLY9
D2V9Q0
TRINITY_DN11080_c4_g1_i1.p1
I4Y7M5
A0A316UT68
V5F1D1
I2G6S2
A0A1K0FYU1

D8LW26
A2FFL0
J9JAC8
A2EAD2
A2EUE8
A0A1J4KZL1
A0A1J4KD55
A0A7S3DNW8
A0A1Z5K7K3
A0A8J7XL72
A0A843BTD3
A0A7C1CCU2
A0A522XYA9
A0A557SVK8
A0A838D347
A0A654MAD2
A0A550GFH8
A0A550GJA5
A0A7C5RKA0
A0A832HX82
A0A7J3Z5U8
A0A6B2C8A9
A0A7C5TWK9
E1QT99
F0QSH4
A0A497GTR0
A0A7J4AYN1
A0A2K2V4B8
A0A2H5UVI2
A0A662PPR2
A0A7C4CNQ4
A0A7C4WGB0
A0A7J2HY37
A0A256Y5X4
A0A7J3NL68
A0A832BXS7

A0A673IPV9
A0A7K8ER36
A0A8C3D6V0
A0A532TY91
A0A524ELR9
A0A850MQ89
V4C1F0
A2EJI6
A0A816MK02
A0A820CEI0
A0A820L5M8
A0A8C5LU88
A0A8C5LUB8
A0A834ITL8
A0A8H7GXT7
A0A2V1B0Y8
A0A2V1AFA3
A0A8J5QGJ0
A5E1E8
C4YM78
A0A1D8PT80
A0A8H6F3V0
A0A1L0BQ30
M7WFW0
TRINITY_DN9732_c0_g2_i1.p1
A2G2G1
A2E2M2
A0A437AKV0
A0A7S3CVP5
A0A8J4V5F7

**UniProtKB ID used in Fig. S7D** *	**UniProtKB ID used in Fig. S7E** *	**UniProtKB ID used in Fig. S7F** *	**UniProtKB ID used in Fig. S7G** *	**UniProtKB ID used in Fig. S7H** *
A0A0C2I723	A0A812IE99	G0QQ18	A0A812IE99	A0A821TKA4
A0A1D2MB91	A9CRJ3	A0A0A8LBP3	A9CRJ3	A0A818Z5J4
A0A1D2MC16	A0A1Y1S664	Q6CJ90	A0A1Y1S664	A0A821JVW3
A0A835CSA2	A0A0R0M7T3	W0T9B2	A0A0R0M7T3	A0A1B7SPZ4
A0A835CQY0	S7W8I7	G8ZY21	S7W8I7	W1Q751
A2DCC5	A0A7S3CVP5	A0A7H9HWT1	A0A7S3CVP5	A0A2G5B9W1
A0A4Q9KY41	A0A8J4V5F7	A0A7G3ZNG9	A0A8J4V5F7	A0A0K8S640
R0MLC0	Q6BZD6	H2ANL1	Q6BZD6	A0A6H5GSU7
A0A6A5BNC4	A0A2V1B0Y8	A0A8H2ZGY6	A0A2V1B0Y8	A0A0K8S654
D2VNZ8	TRINITY_DN14133_c1_g1_i1.p1	C7GQX9	TRINITY_DN14133_c1_g1_i1.p1	A0A0A9XIZ1
B0EK11	A0A812DZM0	B3RHE4	A0A812DZM0	A0A6A4JR53
K2GDQ4	A0A369SI02	G2WJ86	C4YM78	T1IAQ6
A2EXV3	C4YM78	A0A8B8UW66	A0A1D8PT80	A0A0N7Z8L5
Q3SD70	A0A1D8PT80	J6ENU0	A0A8H6F3V0	A0A224XWC7
A0A8S1NU57	A0A8H6F3V0	A0A0L8RFE5	B9WM58	A0A069DQN9
A0A8J5QY30	B9WM58	J8LKE2	C5M3H7	A0A8I6RWP9
A0A0C9QHS2	C5M3H7	G8BUE5	A0A8J5QGJ0	A0A6J0YCN0
E2ATU0	A0A8J5QGJ0	A0A0W0DYK8	A0A8H8DBP2	A0A6P3TNI1
A0A836EZX9	A0A8H8DBP2	Q6FMR0	A0A421JIW5	A0A8C8JIG8
A0A833RY36	A0A421JIW5	J7S7W3	A5E1E8	A2DMY4
A0A154PCY4	A5E1E8	G0VE40	G3B8G4	N9T9B7
A0A7M7GVQ5	G3B8G4	A0A1Q3ABC0	G3B8G3	A0A2G5UH10
A0A6S7GTF6	G3B8G3	A0A1Q2ZX36	A0A1Y2CT12	B6IHN6
TRINITY_DN6741_c0_g1_i1.p1	A0A1Y2CT12	A0A8J2T4F1	A0A1Y3NQ15	E4YE67
A0A1E3P0Y1	A0A1Y3NQ15	A0A1S7HVI1	A0A1Y1XCZ7	A0A7D9CZM6
A0A1A0H717	A0A1Y1XCZ7	A0A7H9B7A8	A0A1Y2D0F9	A2EQ49
A0A512UI12	A0A1Y2D0F9	A7TQ91	A0A2N1J7A6	A0A1J4K8L6
A0A1L0BSK3	A0A2N1J7A6	G8JR72	A0A1E3PR62	A0A1J4KG41
Q6BYB0	A0A1E3PR62	R9XEI8	A0A850MMV7	A0A1J4KRH2
Q5NT15	A0A850MMV7	A0A1G4MCU1	V4C1F0	A0A1J4K6W7
K2GWH3	V4C1F0	C5DEM2	T1FYQ5	A0A6A7G7T8
K2H1L3	T1FYQ5	A0A1G4IXA3	A0A369SI02	A0A6A7GAK5
B0E8D8	Q3SCY3	A0A1E3NXK2	Q3SCY3	A0A1R2CEW5
A0A510P9E2	Q3SDN0	A0A1D2VNR0	Q3SDN0	A0A7S3G5U1
A0A2H0ZHW0	Q3SDK3	G3B8G4	Q3SDK3	A0A7S3G9Z6
A0A0L0NZ33	Q3SCZ8	G3B8G3	Q3SCZ8	A0A1J4KBR9
G8YEB7	A0A8J2F5M1	M3K269	A0A8J2F5M1	A0A1J4K4P4
G8YBW7	A0A8J1ZLN5	A0A0C2MUI2	A0A8J1ZLN5	A0A1J4L2Z7
A0A1B6D225	A0A1R2B4K9	G0R2Q5	A0A1R2B4K9	TRINITY_DN10941_c1_g1_i3.p1
X6MGW6	D2VB98	A0A5J9VNJ2	F0ZUW8	D3YVQ6
A0A0R0M7T3	A0A6P8J1G3	A0A1Y3NQ15	D2VB98	A0A8C6I502
A0A6P7S593	F0ZUW8	A2FIJ0	A0A6P8J1G3	Q0PD39
A0A8B8E6D2	L7K0I4	A2G974	L7K0I4	A0A8C6I458
S6B8L6	A0A0F9ZEV9	A0A1J4KTB4	A0A0F9ZEV9	A0A6P5PI80
L0B2B3	A0A7M7NPW1	A0A1J4K7M7	A0A7M7NPW1	A0A6P5PP20
J4CDX6	A0A7M7RC83	A0A1J4JG74	A0A7M7RC83	B2RZ46
A0A813B3T2	Q3SDK0	A0A1J4JW69	Q3SDK0	A0A218WJE0
A0A8J8T7F8	Q3SDM6	A0A0V0RA50	Q3SDM6	A0A1J4KXA3
A0A3R7W9T9	Q3SDM2	Q3SDI3	Q3SDM2	A0A524DV28
A0A6U9WAJ3	Q3SD42	A0A804KB13	Q3SD42	A0A7M5UI19
A0A1E7EU26	A0A672L021	A0A077Z5W6	A0A672L021	T2MAC0
A0A6A7FZ22	A0A672L036	A0A131ZVZ2	A0A673JC67	A0A183ICB6
A0A0D9YVS9	A0A671LSV7	W6L7P9	A0A6P6R6Z5	S7XTQ9
A0A7J7CM12	A0A673JC67	A0A7S3CVP5	A0A3Q3VRG5	Q3SDI2
	A0A6P6R6Z5	A0A1J4KG86	A0A3Q3VU11	A0CE74
	A0A7J6B401	A0A7C8VL15	A0A667YXN5	Q3SCZ7
	W5UKR5	A0A7C8PHK9	A0A8C4A543	A9UYR4

A0A3Q3VRG5	A0A6G1M4C8	A0A672L036	A0A8B7DFA2
A0A3Q3VU11	A0A7C8NBB5	A0A671LSV7	L8GIC5
A0A667YXN5	A0A369SI02	A0A7J6B401	L8HAR7
A0A8C4A543	A0A497QHU6	W5UKR5	A0A1C7NPA6
A0A3B4DB85	A0A5B9DE64	A0A3B1JUY8	A0A8H7RBX5
M4ANU7	A0A1V0SEB6	A0A5N5Q526	A0A8H7BVQ6
A0A3B5MRM0	I3ER36	M4ANU7	V4A2G3
A0A3Q4AL16	A2DRV6	A0A3B5MRM0	A0A6J8BEV0
A0A8C2WLV0	A0A0A1U9N5	A0A3Q4AL16	A0A8B6D857
A0A6J2W5V0	A0A7S1XW42	A0A8C2WLV0	A0A2K6F864
A0A5N5Q526	A0A7J6EUN9	A0A6J2W5V0	A0A2K6F866
A0A3B1JUY8	R0MLC0	A0A3B4DB85	A0A833R3N8
A0A8C9SBL6	TRINITY_DN16833_c1_g1_i5.p1	A0A8C9SBL6	A0A328CY65
A0A2U3W9G9	A0A890D766	A0A2U3W9G9	A0A1J5WW56
M3Y931	A0A2H0ZHW0	A0A3Q7SCR8	A0A813S638
A0A2Y9IDN2	A0A510P9E2	F1PTE3	A0A815Q636
A0A2U3W4P2	A0A0L0NZ33	A0A6J2ELM6	A0A819MUY6
A0A6J2ELM6	A3GFW1	A0A2U3W4P2	A0A815LWT6
A0A452SH85	Q6BZD6	A0A452SH85	A0A819MLH3
A0A3Q7SCR8	A0A812DZM0	M3Y931	A0A814PMR1
F1PTE3	A0A146K9N6	A0A2Y9IDN2	A0A819M2X1
A0A8C5WCM5	I4Y7M5	A0A8C5WCM5	A0A813XYH8
A0A1I8GTC3	A0A4V4ML05	A0A1I8GTC3	A0A814M3X0
A0A7S1EZY4	A0A4T0QER5	A0A7S1EZY4	A0A814B7Z5
A0A7S2IRI7	A0A4T0FQE2	A0A7S2IRI7	A0A814LNX5
A0A813DRP3	A0A2G5B6K2	A0A813DRP3	A0A815WYL7
A0A812JE06	A0A1J4L1H1	A0A812JE06	A0A815FY79
A0A812TX60	A0A1J4K4P4	A0A812TX60	A0A819USI6
A0A813AUC2	A0A1J4KKP2	A0A812K2K7	A0A816Z0F8
A0A1Q9CR86	Q23W08	A0A813AUC2	A0A815HVY4
A0A812K2K7		A0A1Q9CR86	A0A818FR11
			A0A818HMD5
			A0A817DM21
			A0A818JL34
			A0A818MM80
			A0A820Q620
			A0A821BPC6
			A0A818SBF8
			A0A813QBB6
			A0A816CFM2

UniProtKB ID used in Fig. S7I *	UniProtKB ID used in Fig. S7J *	UniProtKB ID used in Fig. S7K *	UniProtKB ID used in Fig. S7L *	UniProtKB ID used in Fig. S7M *
A0A1E4RHJ0	A0A3M1UVP2	A3GFW1	I1JKY2	A0A1W0E6K4
A0A8B9J6F6	L7K0I4	B0ESL8	A0A0B2SHG0	A0A8H7GXT7
Q6FGX3	L2GVC0	K2HM75	K7K3L6	A0A8C7HQG5
G5E2Z7	S7XTQ9	M7WHL4	I7MDK8	A0A8C7ML11
A0A8M1FTS7	T0MER4	A0A1J4KV97	G0QU16	A0A4W5RCX9
A0A662YRE0	A0A059F2S3	A2FIJ0	I7LY19	A0A1S3SZL2
A0A3Q2NP45	A0A1R2BEZ6	A0A0V0RA50	A0BU91	A0A1S3SZM7
A0A8C7FF29	A0A7S4P836	A0A7S3K8N0	A0A5J4VWM7	A0A1S3SZL4
A0A0S7H0U4	C5LKH4	A0A078ABN3	TRINITY_DN10457_c0_g1_i1.p1	A0A1S3SZK7
A0A2U9B8I3	A0A812Q5K2	A0A0V0QFZ1	B0E8E1	A0A1S3SZL6
A0A4W5LAI3	A0A8J2B4L0	A0A8J1ZLN5	M7VW78	A0A1S3SZL0
A0A8C7IGL2	A0A5J5F566	A0A8B7YSX1	A0A7J6YYW5	A0A8C7HRP8
A0A8C7IDH7	A0A0J9X8R7	S7W8I7	T1EJ40	A0A8C8D7G3
A0A8C8LLP2	A0A5A9MZE3	A9CRJ3	A0BTF9	A0A8C7Q6K0
A0A384DFD5	H9H629	N9URX2	A0A1J4K080	A0A674C9N3
A0A803J357	A0A7S3D6C9	M7WXJ3	A0A7M5TRI7	A0A674C9A5
A0A803K6A7	A0A4D9DI20	K2GIL3	A0A6A5PI19	A0A8C7Q6D8
A0A8C1H6E7	A0A7S4GI31	B0EGQ1	A0A6A4R8Y0	A0A4W5RJY3
Q6P304	Q39861	G0R2Q5	A0A1J7GDT5	A0A8C8CZY7
A0A669BTR5	W4ITV4	A0A1J4KPE5	A0A3P8YVY0	A0A3P8Z3G0
A0A668VST9	A0A2P9GG46	A2DMZ1	A0A8B9LQG6	A0A8C7K7P4
A0A3Q0S8J9	A0A2P9CKT7	A0A1J4JQ83	A0A433PM26	A0A8C8HWB6
A0A667WH53	A0A2P9BSY1	A0A1J4KKP2	A0DHR0	A0A8C7TU69
H2SGX0	Q94659	A0A1J4KCU8	A0A817MCN2	A0A8C7TPK2
A0A3B5A1Y3	Q9UB85	TRINITY_DN12480_c0_g1_i1.p1	A0A433DE42	A0A060VW08
A0A671WCX9	Q9U466	A0A146K690	A0A4P9YVV2	A0A4W5LTD9
A0A3P8SFA4	A0A024VVH2	B0ER43	A0A4P9XJF4	A0A671TEJ5
A0A3Q1F295	A0A060RR02	A0A1X0QHI2	A0A1Y2F8X0	A0A3P8W3Y6
A0A8C6LZA1	A0A2P9D242	S7XUF5	A0A8C4NAN0	A0A3B3ZJE7
H2MNT7	A0A2P9B9K4	A0A842R0Y3	A0A1E3PFL4	A0A3Q3GML6
A0A3P9KVA3	K6UKL1	A0A842R6F5	A0A2U1L0V4	A0A6J2QWT6
A0A3B3CV27	W7ARZ9	A0A0F9DFN3	V7B6H7	A0A6J2QYY2
A0A671NG68	D2VB98	A2EV06	A0A0S3RTU3	A0A8C2WVL9
A0A673HFT2	A0A6A5ATY7	A0A1Y1ZC38	V7B2F7	A0A3Q3GTA3
A0A673HEV8	A0A0N5B7E3	A0A1Y1Z3T9	A0A4D6NGP5	A0A2I4CHV6
A0A671LVR5	A0A0K0EV93	A0A4P9XMJ8	A0A445J787	A0A672GPV2
A0A671M211	A0A0N4Z015	A0A397SAP9	A0A151T9Y3	A0A672GR95
A0A8C1I4S5	A0A090LNS9	A0A372QVP2	A0A2Z6MZY7	A0A6G1PQP7
A0A8C1FT99	A0A0K0E4U5	A0A2Z6RC45	G7KX35	A0A3Q3JV56
A0A671LXY7	A0A4U8URS0	A0A2P4QMJ6	B7FKS6	A0A3B5AQ46
A0A6P6R3B2	A0A6P4YM79	A0A2N1ND68	I3SXF6	A0A3P8SRD2
A0A8N1YWU9	A0A4Y2AL00	A0A1D1Z9A9	G7KX34	A0A2D0SW35
A0A672STW9	A0A1I8I5X4	A0A1D1Y4X4	A0A2K3N539	A0A8J4X8C3
A0A672SSR4	A0A267GBT0	A0A8H4EP33	A0A6P5NKL3	A0A8C1RCY6
A0A672SU05	A0A1I8HBZ8	A0A397UBB2	A0A6J1DXN1	A0A8C1P9W6
A0A672SU16	E4X453	A0A397J5N9	A0A6J1F3N4	A0A6P3WCN8
A0A8C1ZXG6	E4YFK5	A0A507FP66	A0A6J1J588	A0A6P8G119
A0A8C1FUW3	F1LAQ0	F4RPA3	A0A6J1I1J9	A0A6J2VNP7
A0A6P6JR13	A0A0N4V9P2	A0A180GLG9	A0A6J1GBU9	V4C1F0
A0A7J6DCV7	A0A2A6CCI0	A0A2N5T6G8	A0A2C9VBG7	A0A0V1HW41
A0A8C2DXU4	R7TCP3	A0A4T0FQE2	A0A218W3F2	A0A7N4V6I7
A0A8C1L5F7	A0A7M5WZ22	M5E8W1	A0A6I9RAS3	F7EMD6
A0A671NFZ2	A0A023GIH7	E6ZSW7	A0A6I9RRU4	A0A8C0HHI1
A0A672N163	A0A8C3EUA6	A0A2N8U8K1	A0A5B7AKH8	A0A8C3FV55
A0A673HCY8	A0A8D0G4V8	A0A5C3FH31	D7SR02	A0A8C3XUY1
A0A4W4H640	A0A8D1XRF6	A0A8H7PPI3	A0A2P5BS67	A0A8C4UJ25
A0A3B4BVR2	A0A8B9BYZ8	A0A1X2HBN1	A0A8K0H8R8	A0A8C5MSH7
A0A5N5M6K9	A0A3P8WB11	A0A2T9YQM0	A0A6J5XNJ7	A0A8J1KXT4

A0A8C4BDM6
A0A2D0RVW6
A0A3B1K8N3
V9KRJ0
H3DHF1
A0A8D2JKY5
A0A670K8G6
A0A803SWP1
A0A8C9AWC4
A0A8B7PP98
D3PGL3
A0A482W300
A0A023FTB6
A0A8J9Z090
A0A814DN77
A0A814DVR9
A0A813WYI6
R7TE99
A0A7E6FI07
A0A7I8W2J6
S2JFB7
A0A8H4BS43
A0A0C9LVN5
A0A8H7R068
C4QZ12
Q55ET3
A0A814H4B2
A0A6P7V5T8
TRINITY_DN21322_c0_g1_i1.p1
A0A8K1CRQ3
A0A421JIW5
A0A8J5QGJ0
G3AJT2
H8X559
G8BFM2
A0A1L0BQ30

A0A669PIE6
A0A8C9SBL6
A0A816QNG3
A0A7S3DFN8
A0A7S2J5E9
D2V1N7
Q3SDM2
Q3SDJ6
Q3SD54
Q3SDM6
A0A0C2M9K0
A0A812IE99
A0A812K2K7
G0QZL5
Q3SCY3
A0A814ZZ42
A0A3G4ZV93
A2EXV3
V4C1F0
A0A3Q0KGU5
A0A8F2RLY9
A0A5Q0HU94
C4Y1Y5
TRINITY_DN15383_c0_g1_i6.p1
A0A7S3GPX1
A0A0F9RE60

C5M895
A0A367YIS9
A0A367XW76
A0A0H5CAD7
A0A1E4S882
I2JWX4
A0A7D9CYJ2
A0A448YNW1
A0A8K0J7J9
M1W7M1
F7W162
A0A0B0DJ31
U7PXU8
A0A094GEK6
A0A428NCL8
A0A428P6F5
A0A063CBP1
A0A545UPL6
D3B840
A0A890D766
A0A510P9E2
A0A2H0ZHW0
Q6BZD6
C4YEB1
A0A8H6F1C2

A0A6J5V7B8
A0A5E4EKJ3
A0A7J9E626
A0A7J9BWN7
A0A0D2S189
A0A5D2TN29
A0A5D2BFA5
A0A1U8J4P7
A0A7J8ZF24
A0A7J9N594
A0A7J9IZH6
A0A2U7S651
A0A5D2D7H0
A0A5D3A178
A0A0B0PHB8
A0A7J9BNG4
A0A6P6A8Y4
A0A8B8JSV2
A0A8B8JT80
V7AM67
A0A4D6MQ08
A0A0S3SXJ8
A0A371I7U0

A0A8C8VPJ8
A0A6P8SN84
A0A8M1N0P6
A0A5A9NHF9
A0A4X2JNH9
A0A3Q2PPV4
Q5ZIT5
G3NVY1
A0A7N4NL08
A0A811JV18
A0A1I7S8W3
A7S5Z2
A0A1I8I5X4
A0A267GBT0
G0QZL5
A0A3G4ZJY4
A0A817ZCR9
A0A817JHV4
A0A815JFP7
A0A814C117
A0A814X8Z0
A0A815KTP7
A0A818N9U5
A0A815S408
A0A821P7P9
A0A815BI71
A0A814Y729
TRINITY_DN16011_c1_g1_i13.p1
A0A1J4KQ65
A9CRJ3

UniProtKB ID used in Fig. S7N *	UniProtKB ID used in Fig. S7O *	UniProtKB ID used in Fig. S7P *	UniProtKB ID used in Fig. S7Q *	UniProtKB ID used in Fig. S7R *
A0A8J2F5M1	Q4G292	C5M895	V4C1F0	A0A0L0NEF2
A0A3G4ZT94	A0A146K6A4	A0A367YIS9	A0A7S3GPX1	A0A2K3QHR0
A0A0S8JIQ7	A0A2T6ZM98	A0A367XW76	B9WMJ7	A0A8K0J7J9
A0A0A1U4S6	A0A3N4J5V2	S7W8I7	Q59X89	A0A8K0WQ63
A0A7S4ISA4	A0A292PLE6	A0A437AKV0	C5M895	W3X4Y5
Q4G2B3	Q9HDY0	A9CRJ3	A0A2P7YV86	A0A1V1SRE9
A0A1J4L0X4	Q4G2D0	A0A1W0E6K4	A0A6C1E7V3	A0A7C8MNX7
A0A1J4L2Z7	A0A0V0G7Q0	A0A814XKZ4	G8YBW7	A0A4Z0Z3W2
C5M895	A0A6B2LN26	A0A814QLZ4	A0A0V1PV62	A0A1Y2XGL1
A0A367YIS9	A0A0L0D2G5	A0A815JDL7	Q6BZD6	A0A447CM14
A0A367XW76	A0A7S1YM28	A0A820TZQ3	TRINITY_DN12381_c1_g4_i1.p1	B2AXF0
A0A2P4QMJ6	A2DSR0	A0A816WMC3	A0A842R0Y3	G2QDJ0
A0A2N1ND68	A0A1B7SPZ4	A0A818TY56	A2EGH0	F8MKM8
A0A372QVP2	W1Q751	A0A821C8F7	A0A8K0EKR2	A0A0B0DJ31
A0A1D1Y4X4	A0A6S9AIZ9	A0A815TV65	A0A7D9E2I1	F7W162
TRINITY_DN11656_c0_g1_i2.p1	A0A6U5J0A2	A0A818N9U5	A0A3M7QKZ9	S3DHW5
R4UVI8	B8CFX8	A0A821P7P9	A0A7R8H7D1	A0A2T3B6K3
A0A1E3JPZ7	A0A1J4KE73	A0A815S408	A0A0N5B7E3	A0A8H6FY27
A0A1E3HFS5	A0A1J4JFK0	A0A8B7DFA2	A0A8S1XYM0	A0A2L2U0T5
A0A7S4GJ87	Q4G2A0	A0A5S6QR92	Q3SD54	A0A0B7JT57
A0A1J4KAE0	A0A5N6L5I0	A0A7M5WXS5	A0A8S1S9H3	A0A167A5G7
A0A8B7DFA2	A0A821ZHC2	A0A822FMM7	A0A8S1U6V2	A0A0A1UZN6
A0A2G8LBI1	A0A818DT02	A0A8L2Q1F9	A0A8S1UWG0	A0A0B4HZP5
A0A369RU39	A0A821MGB8	A0A1U7QV97	A0A8S1WE94	A0A7D5Z9U8
A7RL89	A0A817VQV9	A0A3B3QXD3	A0A8S1NHV9	A0A0B4FV86
V4C1F0	A0A813V7X3	A0A6S7GTF6	A0A8S1NCU8	A0A395N7P0
A0A3M7SVY6	A0A818EWQ6	A0A2G8LBI1	Q3SDM2	E0S8K8
A2E2M2	A0A820XAV1	A0A7M5X1D9	A0A8S1Q3Y0	I6UQ87
A2G473	A0A4W4EY14	A0A1I8I446	A0A8S1PM58	I6ZJR5
T1JME3	A0A6P3WB05	A0A1I8I5X4	A0A8S1YEW8	M1JID1
D2W0R0	A0A3B1JCF8	A0A267GBT0	I2H316	A0A0B2UDK7
A0A0F9ZEV9	A0A3B4BP68	A0A0F9ZEV9	A0A0C2I723	A0A8B8C0K3
A0A182P478	A0A7S4E9E6	A0A3G4ZV93	A0A7S3DFN8	A0A3S1H8P1
A0A6E8VSE3	A0A7S3ZYW7	A0A3G4ZT94	A0A2R6XGH9	A0A8C5MQN1
A0A182VEW5	F0YR38	A0A1R2BEZ6	A0A8S1RK63	A7S215
A0A182I2G0	A0A397KZ26	A7RL89	A0A8S1UFZ0	A0A293M7B3
A0A182U3T0	A0A8D9GR65	A0A1J4JW69	A0A8S1LD78	G3AWI7
A0A7S1EZY4	A0A3P5ZWS8	A2ESK3	A0A8S1MMC2	A0A0R3T291
A0A7S3MGG9	A0A6J0N9A9	A0A1J4JBD1	Q3SDN0	TRINITY_DN6159_c0_g1_i1.p1
A0A7L2NHC4	V4MA47	A2G2G1	A0A8S1UJG9	Q3SDI3
A0A7L3M763	A0A087GL06	A2E8M0	Q3SDK3	Q4G295
A0A7L3QA71	A0A178V1R4	A2GBJ0	W7K002	M7W6P6
A0A7K5W7Z0	A0A7G2F456	A0A1J4JSU3	A5K9N1	A0A6V7TNB2
A0A852EWV5	A0A2Z4HJG9	A0A1J4KCA8	A0A1J1H3I3	A0A6V7VDS8
A0A7L0YXX8	R9S9A9	A0A146KAX7	A0A8C9H9P9	A0A1Y1ZC38
A0A852G279	A0A2Z4HJH7	A2G412	A0A6V7T708	V5F1D1
A0A7K5SL79	A0A6D2IHV2	A0A1J4KAE0	A0A6G3MIX9	A0A5C3PZW7
A0A7K7MQT5	G7J3R3	TRINITY_DN13871_c2_g2_i1.p1	L8H896	A0A1Y1YUI7
A0A7K4ULZ9	A0A7J9C4M2	A0A1J4JTG9	A0A7R9XIL7	A0A1Y1Z117
A0A8C4A543	A0A5C7HVA3	A0A1J4JC27	A0A3S0ZT37	A0A8H3QPA1
A0A2I4CSF6	A0A6A3BGW3	A0A1J4L0E6	A0A8B7YSX1	A0A2I1EWS0
A0A3Q3EKQ8	A0A5B6ZN47	A2FI73		A0A8H3XAU5
A0A3B4DB85	L1JL95	Q3SD49		A0A397TZM0
A0A5N5Q526	A0A135SN23	Q3SD15		M2N699
A0A673JC67	A0A1Y1XHS8	B9X1R2		A0A3N4JGL0
A0A7J6B401	A0A167NJP4	A0A2H6K891		A0A1Y1I5H5
W5UKR5	A0A0G4IZJ1	A0A061D9C8		A0A7J7M8Q8

A0A672L021	F0WQ13	J7S7W3	A0A0E9NEX6
A0A8C2WLV0	A0A1D2VNR0	A0A7H9B7A8	I2JWX4
A0A4W4EH74	A0A1E3QZZ2	A0A437AN12	A0A7D9CYJ2
A0A672QSA5	A0A448YGF3	A0A1J4KAP9	A0A875S066
A0A8C9SBL6	A0A875SBY1	A0A1J4KF25	A0A1E4S882
V9LBN5	A0A1E4SZ00	A2EV06	H0H091
A0A452GH23	A0A1E4TMP5	A0A6J8BC13	J5RIJ4
A0A8C4Y8Q3	C4R716	A0A2V1B0Y8	A0A376B2F0
A0A4D9DN93	A7TQ91	A0A2V1AFA3	A0A0P1KTR4
A0A8C8RFF0	A0A0N5AEB6	A0A8F2RLY9	C5DME6
A0A6P7WJQ3	A0A0V0QP51	A0A8H7GXT7	A0A367YIS9
A0A6P7WQX3	G0R2Q5	C4YEB1	A0A367XW76
A0A6P8P719	G0R2Z6	A0A0V1PV62	C5M895
A0A8C5RET6	G0QZW6	A0A812DZM0	A0A8J5BMJ0
A0A098LYT8	Q23FM5	A0A7R9M6F0	A0A8J5BLS6
A0A8C5WCM5	A0EC83	A0A836INF4	A0A3N4LLT7
A0A821Q2A1	Q3SDR7	A0A448YEI3	A0A3N4IQI6
Q561P0	E9I3R6	A0A7L0DYT5	A0A6J3MGU2
Q08AW6	G0QSP4	A0A091NRN7	A0A6A6FC18
I7MHF3	E1CB06	A0A093J334	A0A2I0RXR3
A0A4Z2DEI3	Q24CD0	A0A7K6SFJ9	N1QIF5
A0A7S3LSK1	A0A0V0QP25	A0A850WD84	A0A2G5ICM3
A0A7S0VY16	G0QN99	A0A7L0CYS0	A0A6A7BSU4
D2VB98	B0ESL8	A0A7L1JGW0	A0A2K1QUJ9
A0A7S3D6C9	TRINITY_DN14379_c0_g1_i1.p1	A0A7L1I795	A0A6A6JFC6
A0A7S3D4T3	A0A1J4L2Z7	A0A3B4B770	A0A063CBP1
A0A1J1HA99	Q4FID5	A0A8C6TDV0	A0A151GXK7
A0A0D9QK24	Q4G2B3	A0A2P4QMJ6	A0A0F7ZQD3
A0A1Y3DQI3	A0A1J4JDR8	A0A2N1ND68	A0A2C5Z555
K6UKL1	A0A0V0QMI9	A0A2Z6RC45	A0A8H4PTS8
A0A1B1E0W5	A2EI68	A0A372QVP2	T5A660
A0A7S0GVJ0	A0A146K818	A0A397SAP9	A0A179HZ11
A0A8J1ZLN5	K2HZF0	A0A1D1Y4X4	A0A2S4LAA2
	B0EJW3	A0A1Y1ZC38	
		A0A8H7PPI3	
		A0A0B7N846	

UniProtKB ID used in Fig. S7S
H3ZPY0
A0A7K9WWI1
A0A7L2AY31
A0A7L1SUJ0
A0A672UB01
A0A851Y506
A0A7K4WN53
A0A851F6L6
A0A7K6AAI7
A0A7K5K657
A0A7L1YUW8
A0A7L0J6I4
A0A7K8YZE4
A0A7K7HMY4
A0A093PX51
A0A7R5KUX4
A0A7K6DW71
A0A7K9ZLN4
A0A7L1TJ62
A0A852NNU4
A0A091MQW9
A0A7L0P0G0
A0A091P6R5
A0A7L4A6K9
A0A663E6D2
A0A8B9MXT3
A0A7L0C2Q5
A0A7K6SER2
A0A7K6WP83
A0A1V4JGU5
A0A7K4S2M6
A0A674KCU8
A0A8C3HIH7
M7BLR1
A0A8C3XTW6
A0A7K9M987
A0A7K8PRT3
A0A3L8SQL4
W9XVC4
I1HI21
A0A2R5G1A2
A0A665SW59
A0A3B4XVT7
A0A7C3SYP7
A0A271M1F8
A0A8H7VJ05
A0A3Q4HSR0
A0A8C4BIY7
A0A7K7W9V8
A0A851T1Y0
B0WDG7
G2WCA2
A0A6A6A6U5
Q9QZ84
A0A2R8QAS3
A0A2A3EN12

UniProtKB ID used in Fig. S7T
A0A067CQT3
A0A0A1TYQ4
A0A0A1TZW0
E3N8T3
A0A0B2VJL4
A0A183UYI0
A0A3P7G8C6
F1KY49
A0A183E4B6
A0A238C3C9
A0A0K0F094
A0A0N5B5P7
A0A183UPP3
A0A183VFL5
A0A0F7FJ36
A0A3G1A6P0
A0A6G3LK14
S6A5D1
A0A7C1CET5
A0A7C3RFL1
A0A7C4NJF3
A0A132NZ21
C6LN26
A0A1I5ZVK2
A0A5P9FG66
A0A7Z8LFA9
A0A1Z5JLA5
A0A1Z5K7K3
A0A497KTA2
A0A497N359
A0A497NL17
A0A523VQX2
A0A550GFH8
A0A550GJA5
A0A7C3U715
A0A832GST8
A0A4P2VDB4
A0A7J2VYQ3
A0A7C4BDQ5
A0A7J3Z5U8
A0A7M1UPU6
A0A7J3SKM9
A0A7J2ZGM5
A0A7L4RFL7
A0A1Q9MXI9
A0A4E0PVR7
A0A7D5INX6
A2G4Y4
A0A1J4J6C0
A0A1J4JHP7
A0A1J4K2U0
A0A1J4L0B3
A0A0D8XU78
A0A183BZJ7
A0A4U5MK84
A0A6P8JNP9
B4P8D7

UniProtKB ID used in Fig. S7U
A0A0A1U419
A0A0J7NLU2
A0A151WVR9
A0A195FX94
A0A836G5Y4
A0A836GA01
A0A2A3EPC7
A0A7M7FY08
A0A6J3L2F3
A0A6P3UV69
A0A6P3UDD3
A0A6I9W1B6
E2AVV6
A0A146KMR0
A0A1J4KS07
A0D4Q1
A0A5J5BR95

UniProtKB ID used in Fig. S7V
A0A061IXG7
A0A081B323
W3A4R2
W2RI07
W2XW22
W2JU56
V9G0E8
W2HPW2
W2LZU9
A0A0W8CHQ3
A0A0W8D7D9
A0A329RS92
A0A225WRX5
A0A6A4AKH4
A0A6G0SI28
G4ZAV4
A0A485L1L2
W4HCB8
A0A1E7ERA3
A0A448Z563
A0A1Z5JT78
A0A1Z5KGE1
A0A8J9X7B7
A0A6U6EFP9
A0A7S4IG93
K0TD91
A0A6H5KS84
A0A6V1NP81
A0A196SCC1
A0A196SHI8
A0A0L0DGH7
A0A0L0DSS8
A0A0S4IYE4
A0A7S1KZ37
A0A7S1J1G0
A0A7S4GF14
A0A0B7A4Z5
A0A7I8VYK0
A0A132AE08
A0A834R0H4
A0A6F9DH37
A0A1D1VY25
A0A7M7JD61
A0A7M7JHA0
A0A7M7JDD3
A0A0A1TZW0
A0A644F932
A8B5G7
E1F524
A0A6G3LK14
S6A5D1
A2FGA9
A0A1J4J0K8
A0A226ED05
A0A087HKP6
A0A087HKP7
A0A178WIX8

UniProtKB ID used in Fig. S7W
A0A0C3DNR7
A0A139GX96
A0A139HDY9
A0A139HE38
A0A8H6R9V0
M3B473
N1PPB3
A0A1Y6LCR3
F9X5K1
A0A2H1G483
A0A1C5XZZ2
A0A2D0TBD0
A0A3B4BG89
A0A2S7QNH0
W9CRX6
A0A292QA45
A0A2T7A7X9
A0A8H6CJU6

B4I7X4
B4QGM3
Q7KVM5
O77294
Q8SXA5
A0A7S0AJV3
A0A1J4K7E9
A0A132NU74
E1F400
A8B747
A0A4Z1SM88
A0A132NVA3
C6LUS8
A8B748
V6TNS6
E1F401
A0A0R3WSU4
A0A5D2UNJ7
A0A0V0Q868
A0A1D3CZ57
A0A6P6RYW6
A0A212EII7
A0A2H1X0P5
A0A6J1X8S4
E4X4C7
E4YI80
Q5C227
A0A7S1VIC8
A0A7J7IEY4
A0A7S4CJD1
A0A7S1TPF1
A0A7S2RK65
B8BY29
T0RUL9

A0A5S9SDH6
A0A7G2DQD3
A0A1P8AQ82
A0A1J4MQL9
B6AEM6
A0A0N4UH75
A0A6A5DL12
A0A8C1JXR9
A0A834CN59
A0A409WJC1
A0A5N6DP27
A0A6A6JG40
A0A448WL55
A0A6C0ECQ0
A0A3P7LUJ7
A0A6G0YTM2
A0A1E5R165
A0A2P2I617
A0A6A7G8M1
A0A6Q2XRZ8
A0A4Z2DR72
A0A7S1SFW7
A0A812SLM4
A0A812Y9I1
A0A812SUV1
A0A813D987
A0A813G871
A0A813E6D2
A0A813ES76
A0A813ILK3
A0A813L8A9
A0A813JKW0
A0A813ENB4
A0A813G2P3
A0A7S2QNK6
A0A7S4DZM0
A0A7S1M792
A0A1G4I817
Q583K6
A0A3L6LAG3

UniProtKB ID used in Fig. S7X	UniProtKB ID used in Fig. S7Y	UniProtKB ID used in Fig. S7Z	UniProtKB ID used in Fig. S7AA	UniProtKB ID used in Fig. S7AB
A0A1J4J8K4	A0A1R2B755	A0A7S4MA53	A0A381WSD4	A0A0F9WHB2
I7LTW8	A0A6V7RZD1	A0A6A5PKL5	A2EJI5	A0A0V0R280
G0QLE1	A0A6V7TAY8	A0A5N5MCT4	A0A1J4KK47	A0A1J4J277
A0A1J4KI54	W7AKE4	A0A4D9BTN4	A0A1J4J5X7	A0A1J4JS52
A2G473	A0A6V7SJJ4	A0A445AB95	A0A1J4JM42	A0A1J4JKF4
B0ESL8	A0A449BX12	A0A444ZRU0	A0A1J4J1W8	A2DDL1
A2E2M2	V7PWG1	A0A445AAU9	A2DPH2	A2DR69
A0A3Q4HJF3	A0A078KAX5	A0A444ZRR5	A0A1J4JF19	A2DLG2
A0A668UPV9	A0A1C6YJ52	A0A5N5P234+A500	A0A1J4L0F6	A2DXR8
A0A3Q3KP92	A0A1C6YPX8	A0A2P5XWQ2	A0A1J4K3E3	A2EGJ2
A0A6A5ED41	A0A2C9JIF3	A0A1D6PJC2	A2DYG6	A2FY69
A0A484D204	A0A210R7P5	A0A818JZY3	A0A1J4K1G9	A2DWP7
A0A8C9ZDF8	A0A816GMA8	A0A8J5FVM1	A0A1J4KPC6	A2DL53
A0A8C9Z5M5	A0A814NQP4	A0A445FSN0	A0A1J4K5B7	A2FUI1
A0A3Q4BUT6	A0A814LCP9	A0A445DP98	A0A1J4KEE6	A2E117
A0A8C4Z511	A0A819UDZ7	I7MER0	A2FXH1	A2FID7
A0A8C5ADE2	A0A6F9DMZ3	A0A7J9JW60	A0A1J4JPI8	A2G0M4
A0A8C5AGK9	A0A0V0QUS7	A0A7J9HIX1	A2FK80	A2E9J2
A0A8C4Z911	G8YJ57	A0A7J9M609	A0A1J4KY41	A2FD09
W5NJL9	A0A067NBL4	A0A7J9CF27	A2FZQ5	A2GJC5
W5NJL8	B0I556	A0A7J8VF00	A2DAL7	A0A1J4K281
A0A8C4AG32	V5N917	A0A7J6G4Z6	A0A1J4JQ46	A0A1J4K7T5
A0A3N0XD70	A0A804JWC8	A0A8T2XKP9	A0A1J4JLD7	A2EBM3
E9QB21	A0A2P5C898	A0A2N9GR34	A0A1J4JN56	A0A1J4KDC4
A0A3M7Q9T4	A0A2P5E5Y7	A0A5J4Y1E9	A0A1J4KLH2	A0A1J4KI15
V6LYP9	A0A6D2JGI6	A0A8C8CMB4	A0A1J4J164	A0A1J4K8I1
A0A7R9M589	A0A803ME03	A0A8J2ASF7	M7WIA0	A2D981
A0A5A9NME9	A0A6A4QAE7	B4R2C4	B0E6U0	A2DF81
A0A7J5ZS88	A0A422NME5	S7W5E9	K2H7X2	A2EW46
A0A3Q2ZTK7	A0A163J3W0	A2DEX3	A0A0A1TV77	A2FIY3
A0A3P8T5G6	T1EJ40	A0A1J4JPT4	A0A1R2AUF7	A0A1J4J7M9
A0A3P8T5E3	A0A8S1L2W1	A0A1J4KJ20	G0QKQ4	A0A1J4K7D8
A0A2I4B0H1	A0A8S1W812	B0EGV2	A0A1R2CZW2	A2F8D7
M3ZM01	A0A8S1S0L3	F2TYG9	G0QN96	A2G1U5
A0A6J2QQH5	A0A0A1U0D2	A0A7S2WM62	A0C3W5	A0A1J4JE48
A0A3Q3M723	A0A838DXL1	A0A1F5S4L6	A0BF11	A0A1J4JJL8
A0A3B3ZC47	A0A7C5I534	A0A8J7XL72	A0A1J4JTG1	A0A1J4K5N1
A0A3Q3JP80	T2B550	A0A1J4K4Q3	A0A1J4KXC9	A0A1J4KNE3
A0A3Q2YB76	M1CE33	A0A1J4KAS0	A0A1J4J3Z3	A2DCQ9
A0A669F9J7	A0A4D9C2P2	A0A1J4K230	A0A2T6ZXE1	A2G5L9
A0A3Q0T011	A0A0S4J575	A0A1J4JBY4	A0A5J5ERG7	A0A1J4KXJ9
A0A4W4FBX1	A0A077Z461	A0A1J4KBM0	A0A3N4K202	A0A1J4L356
A0A803XWF9	A0A085MFE0	A0A1J4KMK6	A0A292PVS3	A0A1J4J312
A0A6J2IEX0	A0A5S6QQ87	A0A1J4KGG0	A0A317SEH9	A0A1J4JCF0
A0A218UNS0	A0A443SWH3	A0A448ZIF0	D5G9K3	A0A1J4L119
A0A7K5PIN4	A0A074YCB5	A0A7S3LE49	A0A3N4LVM4	A0A1J4KHE9
A0A1J4KQB1	A0A074W7A7	A0A7S0CDE3	A0A0D6EJH7	A0A1J4JK68
A0A4U5PI34	A0A074X5N7	A0A072VBW5	A0A2S5B088	A2DBC8
A0A6N1NUY3	A0A074XB43	A2Q450	A0A120E829	A2DCX2
A0A6N1NW88	A0A4T0A872	A0A072VCK4	A0A4U0W4G1	A2ED22
R0MLC0	A0A4V6TCA5	A0A2J6K9H7	A0A0V0QPL4	A2G6V3
M1K5Y5	M3IHX8	A0A6S7MHE8	A0A814EWS9	A2FMR7
Q8SW02	G3AUZ8	A0A6J1FXY8	A2DFV7	A0A1J4JRT3
A0A0B2UM37	A0A8J5Q0S6	A0A6J1JF26	A0A6B2KXK6	A0A1J4KLZ2
A0A0V0QP62	A3LSM4	A0A437APR0	A0A6B2L0G7	A0A1J4JBK5
A0A0C3LPW8	A0A0V1PW92	A0A1J4KCS6	A0A3P8XE13	A0A1J4KMA6
A0A0C2XQX9	Q6BTM5	A0A0A1U6Y8	A0A0V0QQU1	A0A1J4KEA6
G4TJQ4	G8JUE0	A0A197K3Z8	J9IDQ8	A0A1J4K5M4

A0A8H3TZN1
V6U403
V6TMU8
Q9GU79
E1F2X5
A0A484E0L2
I7LSW1
Q231B4
A0A8H7S4J3
A0A0C2I723
D3BMH1
A0A7J7JHF2
M7WFW0
A0A1J4JAM7
A0A875SBY1
A0A1R2BA89
A0A1R2B1V3
A0A6A5BCH3
A0A8B8D3Y5
Q23W08
A0A1E4RM66
A0A1Z8JJK0
A0A1V2LQ27
A0A099P792
A0A1J4K3I2
A2DJT9
A0A1J4J8B6

A0A1R2BZJ2

A0A1Y1VGQ9
A0A1Y1VWN3
A0A1Y2ECC1
A0A0B1T0A4
A0A2X0LIH5
A0A2X0M815
A0A1U7LN28
A0A1R1Y2Z4
A0A815BAF5
A0A7L2X781
A0A0R3RIG7
A0A0D8Y5W0
A0A811LNT4
A0A6P9ECR0
A0A6P5NDZ8
A0A8J1ZKR6
A0A0D9QGL2
A0A1D3LGY4
A0A4V0K4K9
A0A1C6XER6
W7AM38
A0A6V7TEC2
A0A6V7RWG2
A0A6V7RXG6
A0A081IBW3
A0A509AI23
Q7RLG9
A0A077Y4Y0
V7PR35
A0A1A8YM92

J9ID37
A0A7W0JEE4
A0A4V4NF50
A0A0D9W5P5
A0A8J5WY44
A0A8J5W3R3
A0A6A6KYJ4
A0A811MS40
A0A061R1F1
A0A7S1T3L6
A0A4T0S531
A0A4T0UDE2
R9ARQ6
A0A4T0HL96
A0A4T0ILR3
A0A5B8MQ60
A0A7S3C6J7
A0A5J5AP43
A0A0K9RIK7
A0A6V7QVD3
A0A830CG88
A0A2G9G885
B6T5R0
A0A7J6CIS0
A0A1J1J6K2
A0A1I7SA30
A0A6V7HSW2
A0A132A5A7
A0A834VEN9
A0A8J2ED74
A0A3G4ZN36
A0A3G4ZZP1
A0A6C0J6C9
A0A6C0HVX1
A0A6C0H7D4
A0A6C0DB04
A0A381RMA6

A0A1J4KJQ5
A2G7D0
A2FHT0
A2FKU7
A0A1J4JRP0
A2ELG8
A0A1J4J998
A0A1J4K0I5
A2DB42
A2E5X3
A0A1J4JAI1
A0A1J4L3X5
A0A6G8MVV9
A0A5K1VLT1
B0ETD3
A2DWP6
A2F3W9
A0A1R2CTL5
A0A397IU75
A0A7S0ZLB8
A0A7S0ZLF1
A0A8S4GIZ7
A0A8S4GPI8
A0A1E5W9P7
A0A2U1L2J1
A0A2I0VIP8
A0A8T3A9R6
A0A371H1L5
A0A438CWV7
A0A438GQK5
E0CUH5
A0A8K0I3U2
A0A6J0JNJ0
A0A2G9HU10
J9HWH8
R0MQ89

UniProtKB ID used in Fig. S7AC	UniProtKB ID used in Fig. S7AD
A0A438FEI1	R7UH49
A0A1J4K5M4	A2DY61
A0A1J4JLD9	F6V890
A0A1J4KPE8	A0A6F9DLG8
A0A1J4JSY7	A0A8J4XSX2
A2DK36	B3RJY9
A2DAT0	T1FQZ0
A2G2A8	A0A7I8W6Z3
A2FID7	A0A6S7GDS7
A2E117	A0A7J7A2M3
A2FBW2	A0CGY4
A2DIR4	A2EIC5
A2G1U7	A7RH03
A2EDQ7	A0A8B8D6Q7
A0A1J4JSA9	A0A158QB13
A2DF81	A0A0N5CYC8
A0A1J4K5N1	E0VVH1
A0A1J4KA14	A0A8J8SVZ6
A0A1J4JF52	A0A834XPU3
A0A1J4KHJ5	A0A813PEX5
A0A1J4JE48	A0A1R2C949
A0A1J4KA25	A0A1R2AP99
A0A1J4JJL8	A0A1R2B613
A0A1J4KRD7	F0GWI9
A2E5X3	A0A133KEI5
A2G2L1	A0A1M5QV77
A0A1J4L3X5	R5IVQ1
A0A1J4J3H2	A0A358M8C2
A0A0V0R280	A0A3B9I2F9
A0A0V0QS39	A0A1M6DI04
A0A2U1L2J1	A0A1U7M316
G0WAQ3	A0A7V6XKZ4
A0A1R2C6E8	A0A285BKP8
A0A1R2C3M4	B7R9Z4
A0A1R2BL46	A0A7Y2L5X5
A0A1R2C1E0	A0A7C6RD96
A0A1R2C1D5	A0A1G7U587
A0A1R2D3G5	A0A1M4UKH6
A0A1R2BD78	I9KVI9
A0A7I8VN78	M8CSL9
A0A8J8NGV7	A0A0B3BPJ8
A0A1R2BH76	A0A5D8QG83
A0A1R2CIQ1	A0A269XM42
A0A1R2BD27	A0A8J7S2V1
F4Q2J2	A0A660Q0G1
A0A151ZFK7	A0A3M1C7C4
A0A7J7J830	A0A660Z1I5
A0A7N4PR21	A0A523NCT1
A0A0V0R1R4	A0A7X8FA76
A0A5N5JUD3	A0A832LCD7
A0A0M0JLC1	A0A3M1N4D4
A0A7S4B0W8	G8BTZ4
J9D5Z6	G0WCG8
R0KXU5	G0VAK7
R0MQ89	A0A8H2ZI03
A0A6G0XI09	A0A1X7R7E2
A0A8E4LYS0	A0A7G3ZJE4
A0A8E4LYR5	A0A7H9HV14

A0A4Y7LEK7	A0A1G4KAP7
A0A4Y7L3K0	F2QLM7
O65366	C4QXS1
K9LWN6	A0A1E4S1A3
A0A6P5YTN1	A0A0H5CIT2
A0A6P5Z1E2	A0A1E3NZD5
A0A6J1CPT5	A0A1E3QYY1
A0A7N2L062	R4XNV4
A0A835F539	A0A1Y2ES38
A0A251UI60	A0A1Y2DDV0
A0A834T595	A0A1Y1VN91
A0A314LBJ9	A0A1Y1X5H4
A0A836DDK2	R7QSN9
A0A3P6A873	A2EAS8
R0GVT9	G0QUM5
A0A1S2XDH5	F0Y2N0
A0A6D2IRL1	A0A8J4V512
A0A1J3H1Z5	V4B415
A0A654G3C9	A0A1J4L2Q9
A0A178UCW3	A0A183KEX8
A0A2Z7AIN2	A0A183NTI0
A0A3N6SDE0	W7WYR6
A0A816QU65	A0A7S2U6B1
A0A0D3D1R9	A0DG76
A0A816ZHN7	A0A7F5R7G2
M4F200	A0A7F5R7F1
A0A067KW19	A0A443QQT7
Q8L5N8	A0A8H3X323
A0A200PW04	A0A835YZ60
A0A2Z0DYH0	A0DJR9
A0A2U1NB55	A0A1R2AUS4
W9R8B8	J9IEM3
A0A4Y7KVZ2	A0BIS4
A0A371ERQ2	A0A8J4PVQ4
A0A1S3TIW5	A0A1J4K477
A0A314KYX7	A0A1R2B8U0
A0A2C9UV00	A0A1R2C538
A0A438BQK6	
A0A438GGA2	

* IDs starting with 'TRINITY' are sequences from Nishimura et al. (2020)

Table S6. Infection rates of clostridial 16S rRNA phylotypes to cells of respective protist hosts.

16S rRNA phylotype	termite colony ID	infection rate (%)
RsTa-C01	A	0
(host protist:	B	0
Trichonymph aagilis)	C	0
	D	9.8
	E	54.7
	F	0.9
	G	71.4
CfP3-15	A *	0
(host protist:	B *	0
Pseudotrichonympha	C *	0
grassii)	D *	0
	E *	0
	F **	74.1
	G **	100.0
	H **	21.0
	I ***	4.0
	J ***	0.9
NkDv07	A	0
(host protist:	B	0
Devescovina sp.)	C	0
	D	0
	E	0
	F	0
	G	0
	H	0
	I	0
	J	0
	K	18.3
	L	0
	M	0
	N	7.2

* From Kagoshima Prefecture.

** From Okinawa Prefecture.

*** From Chiba Prefecture.

Table S7. Single-copy marker genes modified based on the CfP3-15 genome.

domain ID	gene annotation
PF00623	RNA polymerase Rpb1, domain 2
PF00889	Elongation factor TS
PF00886	Ribosomal protein S16
PF11987	Translation-initiation factor 2
TIGR03723	tRNA threonylcarbamoyl adenosine protein
PF01746	tRNA (Guanine-1)-methyltransferase
PF00318	Ribosomal protein S2
PF01765	Ribosome recycling factor
PF00162	Phosphoglycerate kinase
PF00177	Ribosomal protein S7p/S5e
PF03947	Ribosomal Proteins L2, C-terminal domain
PF00673	ribosomal L5P family C-terminus
PF00203	Ribosomal protein S19
TIGR03263	Guanylate kinase
PF01195	Peptidyl-tRNA hydrolase
PF06421	GTP-binding protein LepA C-terminus
PF00380	Ribosomal protein S9/S16
PF04998	RNA polymerase Rpb1, domain 5
PF00252	Ribosomal protein L16p/L10e
PF00453	Ribosomal protein L20
TIGR02432	tRNA(Ile)-lysidine synthetase
PF00333	Ribosomal protein S5, N-terminal domain
PF01245	Ribosomal protein L19
PF00312	Ribosomal protein S15
PF00189	Ribosomal protein S3, C-terminal domain
PF01250	Ribosomal protein S6
PF00562	RNA polymerase Rpb2, domain 6
PF01281	Ribosomal protein L9, N-terminal domain
PF01000	RNA polymerase Rpb3/RpoA insert domain
PF02130	Uncharacterized protein family UPF0054
PF03948	Ribosomal protein L9, C-terminal domain
PF00281	Ribosomal protein L5
PF00828	Ribosomal protein L18e/L15
PF13184	NusA-like KH domain
PF02978	Signal peptide binding domain
PF01795	MraW methylase family
TIGR00755	rRNA small subunit methyltransferase A
PF01016	Ribosomal L27 protein
PF01196	Ribosomal protein L17
TIGR01079	Ribosomal protein L24
TIGR00855	Ribosomal protein L7/L12
PF02033	Ribosome-binding factor A
PF01668	SmpB protein

TIGR03594	Ribosome-associated GTPase EngA
PF00572	Ribosomal protein L13
PF04983	RNA polymerase Rpb1, domain 3
PF00466	Ribosomal protein L10
PF06071	Protein of unknown function (DUF933)
PF00416	Ribosomal protein S13/S18
PF00347	Ribosomal protein L6
PF00831	Ribosomal L29 protein
PF00338	Ribosomal protein S10p/S20e
TIGR00967	Preprotein translocase, SecY subunit
PF04565	RNA polymerase Rpb2, domain 3
PF03719	Ribosomal protein S5, C-terminal domain
TIGR00250	RNAse H domain protein, YqgF family
PF00410	Ribosomal protein S8
PF04997	RNA polymerase Rpb1, domain 1
PF03484	tRNA synthetase B5 domain
PF00297	Ribosomal protein L3
PF05697	Bacterial trigger factor protein (TF)
PF00181	Ribosomal Proteins L2, RNA binding domain
TIGR00344	Alanine--tRNA ligase
PF01632	Ribosomal protein L35
PF05000	RNA polymerase Rpb1, domain 4
PF00573	Ribosomal protein L4/L1 family
TIGR00810	Preprotein translocase, SecG subunit
TIGR02075	UMP kinase
PF00298	Ribosomal protein L11, RNA binding domain
TIGR00459	Aspartate--tRNA ligase
PF00411	Ribosomal protein S11
PF00164	Ribosomal protein S12/S23
PF04561	RNA polymerase Rpb2, domain 2
TIGR00392	Isoleucine--tRNA ligase
PF00238	Ribosomal protein L14p/L23e
TIGR00460	Methionyl-tRNA formyltransferase
PF00861	Ribosomal L18p/L5e family
TIGR00019	Peptide chain release factor 1
PF01193	RNA polymerase Rpb3/Rpb11 domain
PF10385	RNA polymerase beta subunit external 1 domain
PF00276	Ribosomal protein L23
PF08529	NusA N-terminal domain
TIGR00329	Metallohydrolase, glycoprotease/Kae1 family
PF00829	Ribosomal prokaryotic L21 protein
TIGR00922	Transcription termination factor NusG
PF03946	Ribosomal protein L11, N-terminal domain
PF00237	Ribosomal protein L22p/L17e
PF00366	Ribosomal protein S17

PF04563	RNA polymerase beta subunit
PF01409	tRNA synthetases class II core domain (F)
PF04560	RNA polymerase Rpb2, domain 7
PF00687	Ribosomal protein L1p/L10e family

Table S8: MAGs (>50% CheckM or CheckM2 completeness) detected in WGA samples.

WGA sample	bin ID	classification*	total length (bp)	G+C (%)	CheckM** completeness (%)	CheckM2*** completeness (%)	alignment rate (%)	accession number
P. grassii	A	*Termitinemataceae*	2,383,868	45.2	78.5	80.7	9.3	BTHV01000001-BTHV01000265
	B	"*Ca* . Azobacteroides pseudotrichonymphae"	1,290,540	32.8	100	100.0	57.1	BTHW01000001-BTHW01000069
	C	*Treponemataceae*	2,245,192	51.1	48	51.3	5.0	BTHX01000001-BTHX01000339
	CfP3-15#	"Acutalibacteraceae"	1,007,634	31.4	80.2	86.8	29.6	AP027924
T. agilis	A	*Treponemataceae*	3,261,309	48.4	89.9	81.7	1.8	BTHY01000001-BTHY01000805
	B	*Termitinemataceae*	3,602,372	40.5	87.1	95.5	5.3	BTHZ01000001-BTHZ01000496
	C	"*Ca.* Endomicrobium trichonymphae"	1,131,688	35.2	96.6	100.0	51.4	BTIA01000001-BTIA01000030
	D	*Rickettsiales*	1,843,874	28.3	82.3	74.8	1.4	BTIB01000001-BTIB01000375
	E	*Mycoplasmoidaceae*	1,376,671	29.6	76.4	80.3	1.0	BTIC01000001-BTIC01000246
	F	*Termitinemataceae*	2,345,787	39.5	81.2	82.0	2.9	BTID01000001-BTID01000336
	G	*Termitinemataceae*	2,599,803	42.0	83.2	87.6	3.4	BTIE01000001-BTIE01000444
	RsTa-C01#	"Acutalibacteraceae"	1,276,969	27.5	85.3	93.9	14.0	AP027925
Devescovina	A	*Endomicrobium*	885,870	31.7	86.2	90.4	13.7	BTIF01000001-BTIF01000070
	NkDv07#	"Acutalibacteraceae"	1,079,858	33.0	85.3	90.6	74.6	BSWA01000001-BSWA01000004

* Based on GTDB r207.

** Estimated completeness, using the universal set of bacterial marker genes.

*** Estimated completeness, using "General Model".

Bins were after individually processed (see Supplementary Methods); therefore, CfP3-15 and RsTaC01 were complete geonomes.

Table S9. Pseudogenes of endosymbiotic *Clostridia*.

ID [*]	gene annotation based on COG
CfP3-15_0011	Beta-lactamase class A
CfP3-15_0035	Transposase
CfP3-15_0259	Transposase
CfP3-15_0279	Transposase
CfP3-15_0414	Transposase
CfP3-15_0447	Transposase
CfP3-15_0547	Transposase
CfP3-15_0567	Transposase
CfP3-15_0066	Phage-related protein
CfP3-15_0068	Phage-related protein
CfP3-15_0082	Transposase and inactivated derivatives, IS1 family
CfP3-15_0271	Transposase and inactivated derivatives, IS1 family
CfP3-15_0458	Transposase and inactivated derivatives, IS1 family
CfP3-15_0487	Transposase and inactivated derivatives, IS1 family
CfP3-15_0594	Transposase and inactivated derivatives, IS1 family
CfP3-15_0595	Transposase and inactivated derivatives, IS1 family
CfP3-15_0645	Transposase and inactivated derivatives, IS1 family
CfP3-15_0767	Transposase and inactivated derivatives, IS1 family
CfP3-15_0925	Transposase and inactivated derivatives, IS1 family
CfP3-15_0270	ATP-dependent Lon protease, bacterial type
CfP3-15_0286	DNA uptake channel protein ComEC, N-terminal domain
CfP3-15_0296	DNA-binding transcriptional regulator, PucR/PutR family
CfP3-15_0306	Signal recognition particle GTPase
CfP3-15_0312	DNA repair protein RadA/Sms, contains AAA+ ATPase domain
CfP3-15_0658	DNA repair protein RadA/Sms, contains AAA+ ATPase domain
CfP3-15_0388	Transposase InsA
CfP3-15_0417	DNA replication protein DnaC
CfP3-15_0517	DNA replication protein DnaC
CfP3-15_0774	DNA replication protein DnaC
CfP3-15_0623	ATP-dependent DNA ligase
CfP3-15_0773	ATP-dependent DNA ligase
CfP3-15_0626	Holliday junction resolvase RusA (prophage-encoded endonuclease)
CfP3-15_0639	PASTA domain, binds beta-lactams
CfP3-15_0687	GTPase SAR1 family domain
NkDv07_0149	16S rRNA G527 N7-methylase RsmG (former glucose-inhibited division protein B)
NkDv07_0017	Predicted transcriptional regulator, contains HTH domain

NkDv07_0247	Fatty acid-binding protein DegV (function unknown)
NkDv07_0344	DNA repair protein RadA/Sms, contains AAA+ ATPase domain
NkDv07_0369	DNA repair protein RadA/Sms, contains AAA+ ATPase domain
NkDv07_0390	Transposase
NkDv07_0414	Transposase
NkDv07_0415	Transposase
NkDv07_0439	Transposase
NkDv07_0455	Transposase
NkDv07_0480	Transposase
NkDv07_0500	Transposase
NkDv07_0545	Transposase
NkDv07_0584	Transposase
NkDv07_0611	Transposase
NkDv07_0786	Transposase
NkDv07_0795	Transposase
NkDv07_0798	Transposase
NkDv07_0799	Transposase
NkDv07_0824	Transposase
NkDv07_0967	Transposase
NkDv07_0982	Transposase
NkDv07_0418	DNA replication protein DnaC
NkDv07_0451	DNA replication protein DnaC
NkDv07_0423	Uncharacterized protein domain, C-terminal to COG1479 DNAse/DNA nickase
NkDv07_0431	2-polyprenyl-3-methyl-5-hydroxy-6-metoxy-1,4-benzoquinol methylase
NkDv07_0435	Transposase
NkDv07_0457	Holliday junction resolvase RusA (prophage-encoded endonuclease)
NkDv07_0463	5-methylcytosine-specific restriction endonuclease McrA
NkDv07_0789	5-methylcytosine-specific restriction endonuclease McrA
NkDv07_0465	ATP-dependent DNA ligase
NkDv07_0791	ATP-dependent DNA ligase
NkDv07_0467	Lipopolysaccharide biosynthesis protein, LPS:glycosyltransferase
NkDv07_0470	Phage-related protein
NkDv07_0474	Site-specific DNA recombinase SpoIVCA/DNA invertase PinE
NkDv07_0493	Site-specific DNA recombinase SpoIVCA/DNA invertase PinE
NkDv07_0531	Site-specific DNA recombinase SpoIVCA/DNA invertase PinE
NkDv07_0475	DNA-directed RNA polymerase specialized sigma subunit, sigma24 family
NkDv07_0591	DNA-directed RNA polymerase specialized sigma subunit, sigma24 family
NkDv07_0476	DNA modification methylase
NkDv07_0589	DNA modification methylase

NkDv07_0496	Phage antirepressor protein YoqD, KilAC domain
NkDv07_0528	Phage antirepressor protein YoqD, KilAC domain
NkDv07_0498	Lyzozyme M1 (1,4-beta-N-acetylmuramidase), GH25 family
NkDv07_0526	Lyzozyme M1 (1,4-beta-N-acetylmuramidase), GH25 family
NkDv07_0577	Transposase and inactivated derivatives, IS1 family
NkDv07_0612	Transposase (or an inactivated derivative)
NkDv07_0775	Transposase (or an inactivated derivative)
NkDv07_0793	Phage portal protein BeeE
NkDv07_0794	Predicted phage phi-C31 gp36 major capsid-like protein
NkDv07_0952	Signal transduction histidine kinase
NkDv07_0968	DNA-binding transcriptional regulator, MerR family
NkDv07_0979	Prophage antirepressor
NkDv07_0984	Membrane protein TolA involved in colicin uptake
RsTa-C01_0020	ATP-dependent helicase/DNAse subunit B
RsTa-C01_0029	Ribonuclease HII
RsTa-C01_0043	REP element-mobilizing transposase RayT
RsTa-C01_0084	REP element-mobilizing transposase RayT
RsTa-C01_0216	REP element-mobilizing transposase RayT
RsTa-C01_0234	REP element-mobilizing transposase RayT
RsTa-C01_0260	REP element-mobilizing transposase RayT
RsTa-C01_0298	REP element-mobilizing transposase RayT
RsTa-C01_0403	REP element-mobilizing transposase RayT
RsTa-C01_0525	REP element-mobilizing transposase RayT
RsTa-C01_0783	REP element-mobilizing transposase RayT
RsTa-C01_1099	REP element-mobilizing transposase RayT
RsTa-C01_0044	Retron-type reverse transcriptase
RsTa-C01_0052	Retron-type reverse transcriptase
RsTa-C01_0055	Retron-type reverse transcriptase
RsTa-C01_0204	Retron-type reverse transcriptase
RsTa-C01_0274	Retron-type reverse transcriptase
RsTa-C01_0349	Retron-type reverse transcriptase
RsTa-C01_0371	Retron-type reverse transcriptase
RsTa-C01_0388	Retron-type reverse transcriptase
RsTa-C01_0402	Retron-type reverse transcriptase
RsTa-C01_0712	Retron-type reverse transcriptase
RsTa-C01_0975	Retron-type reverse transcriptase
RsTa-C01_0990	Retron-type reverse transcriptase
RsTa-C01_0058	Transposase
RsTa-C01_0076	Transposase

RsTa-C01_0146	Transposase
RsTa-C01_0245	Transposase
RsTa-C01_0278	Transposase
RsTa-C01_0290	Transposase
RsTa-C01_0424	Transposase
RsTa-C01_1090	Transposase
RsTa-C01_1098	Transposase
RsTa-C01_0089	Transposase
RsTa-C01_0593	Transposase
RsTa-C01_0611	Transposase
RsTa-C01_0713	Transposase
RsTa-C01_0765	Transposase
RsTa-C01_0778	Transposase
RsTa-C01_1086	Transposase
RsTa-C01_0122	ABC-type polar amino acid transport system, ATPase component
RsTa-C01_0138	Adenine specific DNA methylase Mod
RsTa-C01_0383	Adenine specific DNA methylase Mod
RsTa-C01_0139	Superfamily II DNA or RNA helicase
RsTa-C01_0140	Transposase
RsTa-C01_0188	Transposase
RsTa-C01_1124	Transposase
RsTa-C01_0141	Magnesium-transporting ATPase (P-type)
RsTa-C01_0150	Superfamily II DNA or RNA helicase, SNF2 family
RsTa-C01_0155	Type I restriction-modification system, DNA methylase subunit
RsTa-C01_0186	Transposase and inactivated derivatives, IS5 family
RsTa-C01_0230	Lantibiotic modifying enzyme
RsTa-C01_0322	Small-conductance mechanosensitive channel
RsTa-C01_0348	Alanyl-tRNA synthetase
RsTa-C01_0377	Primase-polymerase (Primpol) domain protein
RsTa-C01_0391	Phage-related protein YomH
RsTa-C01_0916	Phage-related protein YomH
RsTa-C01_0399	Lysophospholipase, alpha-beta hydrolase superfamily
RsTa-C01_0400	Phage portal protein BeeE
RsTa-C01_0410	5-methylcytosine-specific restriction endonuclease McrA
RsTa-C01_0460	ATP-dependent Clp protease, ATP-binding subunit ClpA
RsTa-C01_0529	Predicted Mg-chelatase, contains ChlI-like and ATPase domains, YifB family
RsTa-C01_0551	DNA uptake channel protein ComEC, N-terminal domain
RsTa-C01_0583	DNA uptake channel protein ComEC C-terminal domain, metallo-beta-lactamase superfamily
RsTa-C01_0643	CRISPR-associated protein Csa3, CARF domain

RsTa-C01_0848	Aspartate kinase
RsTa-C01_0849	Translation elongation factor EF-4, membrane-bound GTPase
RsTa-C01_0863	Serine kinase of the HPr protein, regulates carbohydrate metabolism
RsTa-C01_0870	L,D-peptidoglycan transpeptidase YkuD, ErfK/YbiS/YcfS/YnhG family
RsTa-C01_0871	Periplasmic beta-glucosidase and related glycosidases
RsTa-C01_0883	mRNA-degrading endonuclease MazF, toxin component of the MazEF toxin-antitoxin module
RsTa-C01_0889	Diaminopimelate epimerase
RsTa-C01_0999	Hydroxymethylpyrimidine/phosphomethylpyrimidine kinase
RsTa-C01_1000	Hydroxyethylthiazole kinase, sugar kinase family
RsTa-C01_1125	Uncharacterized conserved protein, DUF917 family
RsTa-C01_1126	N-methylhydantoinase A/oxoprolinase/acetone carboxylase, beta subunit

* Pseudogenes not assigned to any COG are not listed.

Table S10: Eukaryotic-like genes detected in the genomes of endosymbiotic *Clostridia*.

ID	domain*	RT-PCR**	most closely related organism	signal peptide prediction
CfP3-15_0039	Rab	n.t.	*Tritrichomonas foetus*	Sec signal peptide
CfP3-15_0049	Rho	not detected	*Naegleria gruberi*	Sec signal peptide
CfP3-15_0165	PP2Ac	not detected	*Amphiprora paludosa*	not detected
CfP3-15_0235	Rab	n.t.	*Trichomonas vaginalis*	not detected
CfP3-15_0282	Rab	n.t.	*Trichomonas vaginalis*	not detected
CfP3-15_0445	Rab	n.t.	*Trichoplax* sp. H2	Sec signal peptide
CfP3-15_0456	Rab	n.t.	*Pseudotrichonympha grassii*	not detected
CfP3-15_0548	Rab	n.t.	*Candidatus* Helarchaeota archaeon	Sec signal peptide
CfP3-15_0552	Roc	detected	*Tritrichomonas foetus*	Sec signal peptide
CfP3-15_0565	Rab	n.t.	*Brachionus calyciflorus*	not detected
CfP3-15_0581	Rab	n.t.	*Pseudotrichonympha grassii*	not detected
CfP3-15_0591	Rab	n.t.	*Trepomonas* sp. PC1	Sec signal peptide
CfP3-15_0627	S_TKc	not detected	*Protomyces lactucae-debilis*	not detected
CfP3-15_0653	Rab	n.t.	*Metschnikowia persimmonesis*	Sec signal peptide
CfP3-15_0780	Rab	n.t.	*Cryptococcus amylolentus*	Sec signal peptide
CfP3-15_0790	Gem1	not detected	*Trepomonas* sp. PC1	not detected
CfP3-15_0852	Rab	not detected	*Pseudotrichonympha grassii*	Sec signal peptide
CfP3-15_0856	Rab	n.t.	*Trichomonas vaginalis*	not detected
CfP3-15_0929	Rab	n.t.	n.t. (no significant blast hits)	Sec signal peptide
CfP3-15_0934	Rab	n.t.	*Paramecium tetraurelia*	not detected
RsTa-C01_0144	Rab	not detected	*Ichthyophthirius multifiliis*	Sec signal peptide
RsTa-C01_0199	S_TKc	n.t.	candidate division WOR-3 bcterium	not detected
RsTa-C01_0208	PP2Ac	n.t.	*Candidatus* Falkowbacteria bacterium	not detected
RsTa-C01_0269	LRR_5	n.t.	n.t. (repeat protein)	not detected
RsTa-C01_0431	UCH	detected	*Candida inconspicua*	not detected
RsTa-C01_0505	LRR_5	n.t.	n.t. (repeat protein)	not detected
RsTa-C01_0710	LRR_5	n.t.	n.t. (repeat protein)	not detected
RsTa-C01_0762	Myb_DNA-binding	n.t.	n.t. (too short alignment)	not detected

RsTa-C01_0774	Myb_DNA-binding	not detected	*Trichonympha agilis*	not detected
RsTa-C01_0775	Myb_DNA-binding	n.t.	*Trichonympha agilis*	Lipoprotein signal peptide
RsTa-C01_0844	S_TKc	detected	*Trichonympha agilis*	Sec signal peptide
RsTa-C01_0845	LRR_5	n.t.	n.t. (repeat protein)	Sec signal peptide
RsTa-C01_0983	PHA03095	n.t.	n.t. (repeat protein)	not detected
RsTa-C01_0984	PHA03095	n.t.	n.t. (repeat protein)	not detected
RsTa-C01_0992	S_TKc	n.t.	*Stentor coeruleus*	Sec signal peptide
NkDv07_0114	Myosin_tail_1	n.t.	*Apis cerana cerana*	Sec signal peptide
NkDv07_0121	Metallophos	n.t.	Rhodobacteraceae bacterium	Sec signal peptide
NkDv07_0395	Gem1	n.t.	n.t. (no significant blast hits)	Sec signal peptide
NkDv07_0512	Rnd2_Rho7	n.t.	*Nyssa sinensis*	Sec signal peptide
NkDv07_0536	MPP_PPP_family	n.t.	*Trichomonas vaginalis*	not detected
NkDv07_0615	OGFr_N	n.t.	*Letharia lupina*	not detected
NkDv07_0673	PTZ00341	n.t.	n.t. (too short alignment)	Sec signal peptide

* Rab, Ras-related in brain (Rab) family of small guanosine triphosphatases (GTPases); Rho, Ras homology family (Rho) of small guanosine triphosphatases (GTPases); PP2Ac, Protein phosphatase 2A homologues, catalytic domain; Roc, Ras of Complex, Roc, domain of DAPkinase; S_TKc, Serine/Threonine protein kinases, catalytic domain; Gem1, GTPase SAR1 family domain; LRR_5, Leucine rich repeats (6 copies); UCH, Ubiquitin carboxyl-terminal hydrolase; Myb_DNA-binding, Myb-like DNA-binding domain; PHA03095, ankyrin-like protein; Myosin_tail_1, Myosin tail; Metallophos, Calcineurin-like phosphoesterase; Rnd2_Rho7, Rnd2/Rho7 GTPases; MPP_PPP_family, phosphoprotein phosphatases of the metallophosphatase superfamily, metallophosphatase domain; OGFr_N, Opioid growth factor receptor (OGFr) conserved region; PTZ00341, Ring-infected erythrocyte surface antigen.

** n.t., not tested.

Table S11: General features of MAGs in the small genome clade.

accession number	host	geographic range	total length (bp)	number of contigs	G+C (%)	completeness (%)[*]	predicted genome size (Mbp)
GCA_902796915.1	cattle	Europe	1404720	145	31.0	100.0	1.40
GCA_017406805.1	goat	Asia	1157853	53	33.9	97.8	1.18
GCA_017414725.1	goat	Asia	864431	150	32.3	92.4	0.94
GCA_017431755.1	goat	Asia	812295	116	32.3	94.6	0.86
GCA_017432165.1	goat	Asia	1234861	103	33.7	98.9	1.25
GCA_017448285.1	goat	Asia	869701	78	32.4	97.8	0.89
GCA_000435275.1	human	North America, Europe, Asia, Oceania	1114344	132	34.5	96.7	1.15
GCA_900552265.1	human	Europe, Asia, Oceania	1119698	17	32.9	100.0	1.12
GCA_900554335.1	human	South America	1073904	14	37.3	98.9	1.09
GCA_900554775.1	human	North America, Europe, Asia, Oceania	864877	127	38.2	91.3	0.95
MGYG000000505	human	Oceania, Africa	1142930	172	34.9	84.8	1.35
MGYG000000607	human	Africa	1023795	31	35.6	96.7	1.06
MGYG000001125	human	Asia	1115602	240	33.7	83.7	1.33
MGYG000001801	human	Europe	1294689	229	35.1	90.2	1.44
MGYG000003453	human	Oceania	825696	257	33.1	75.0	1.10
MGYG000004322	human	Europe, Asia	1235249	21	34.6	100.0	1.24
MGYG000004437	human	North America, Europe, Asia, Oceania, South America, Africa	1139688	15	35.3	100.0	1.14
MGYG000004496[***]	human	Asia	1393460	182	33.6	73.9	1.89
GCA_017480265.1	roe deer	Asia	883795	85	36.7	98.9	0.89
GCA_002372375.1	sheep	Oceania	1440018	55	34.5	100.0	1.44

GCA_017515685.1	sheep	Asia	853097	306	39.2	72.8	1.17
GCA_009785985.1	soil-feeding termite (*Isognathotermes ugandensis*)	Africa	1238722 (2692270)**	277 (648)**	30.4 (30.5)**	90.2 (92.4)**	1.4 (2.9)**
GCA_017634155.1	water deer	Asia	912766	64	30.8	97.8	0.93

* Caluculated based on modified single-copy marker gene sets (Table S7).

** Before re-binning.

*** Detected single-copy marker gene set was considerably different from other members of the small genome clade;

Table S12: Descriptions of the eukaryotic-like domains detected in the small genome clade.

domain	description
STKc_MAP	Aldo/keto reductase, related to diketogulonate reductase
AKR_AKR1-5-like	AKR1/2/3/4/5 family of aldo-keto reductase (AKR) and similar proteins
Myb_DNA-binding	Myb-like DNA-binding domain
S_TKc	Serine/Threonine protein kinases, catalytic domain
PKc	Catalytic domain of Protein Kinases
STKc_KSR1	Catalytic domain of the Serine/Threonine Kinase, Kinase Suppressor of Ras 1
STKc_Chk1	Catalytic domain of the Serine/Threonine kinase, Checkpoint kinase 1
SPS1	Serine/threonine protein kinase
STKc_MAPKKK	Catalytic domain of the Serine/Threonine Kinase, Mitogen-Activated Protein Kinase Kinase Kinase
Pkinase	Protein kinase domain
MPP_PPP_family	Phosphoprotein phosphatases of the metallophosphatase superfamily, metallophosphatase domain
PP2Ac	Protein phosphatase 2A homologues, catalytic domain
PTPc	Catalytic domain of protein tyrosine phosphatases
LRR_5	Leucine rich repeats (6 copies)
TPR	Tetratricopeptide repeat
Ank_2	Ankyrin repeats (3 copies)
PHA03095	Ankyrin-like protein
PHA02874	Ankyrin repeat protein
PHA03100	Ankyrin repeat protein
Rab	Ras-related in brain (Rab) family of small guanosine triphosphatases (GTPases)
Rab6	Rab GTPase family 6
Roc	Ras of Complex

Rho	Ras homology family (Rho) of small guanosine triphosphatases (GTPases)
Ras_like_GTPase	Rat sarcoma (Ras)-like superfamily of small guanosine triphosphatases (GTPases)
Rnd2_Rho7	Rnd2/Rho7 GTPases
Gem1	GTPase SAR1 family domain
UCH	Ubiquitin carboxyl-terminal hydrolase
Peptidase_C19	Peptidase C19
zf-RING_2	Ring finger domain
Tcp10_C	T-complex protein 10 C-terminus
Lipase_3	Lipase (class 3)
PLN02486	Aminoacyl-tRNA ligase
PTZ00341	Ring-infected erythrocyte surface antigen
Metallophos	Calcineurin-like phosphoesterase
OGFr_N	Opioid growth factor receptor (OGFr) conserved region
Methyltransf_FA	Farnesoic acid 0-methyl transferase
Myosin_tail_1	Myosin tail
PTZ00121	Merozoite apical erythrocyte binding ligand
PLN03091	Hypothetical protein
CCDC158	Coiled-coil domain-containing protein 158.

Table S13: Genes predicted to have been acquired in the last common ancestor of the small genome clade.

COG/orthogroup	description
COG3202	ATP/ADP translocase
COG2094	3-methyladenine DNA glycosylase Mpg
COG0531	serine transporter YbeC
COG3740	phage head maturation protease
OG0000732	NHLM bacteriocin system secretion protein
OG0000787	multidrug and toxic compound extrusion-like protein
OG0001223	leucine rich repeats containing protein
OG0002080	head-tail connector protein gp6
OG0002469	phage tail protein
OG0000920	hypothetical protein
OG0001554	hypothetical protein
OG0003398	hypothetical protein
OG0004343	hypothetical protein
OG0005233	hypothetical protein
OG0005234	hypothetical protein

Supplementary Figures

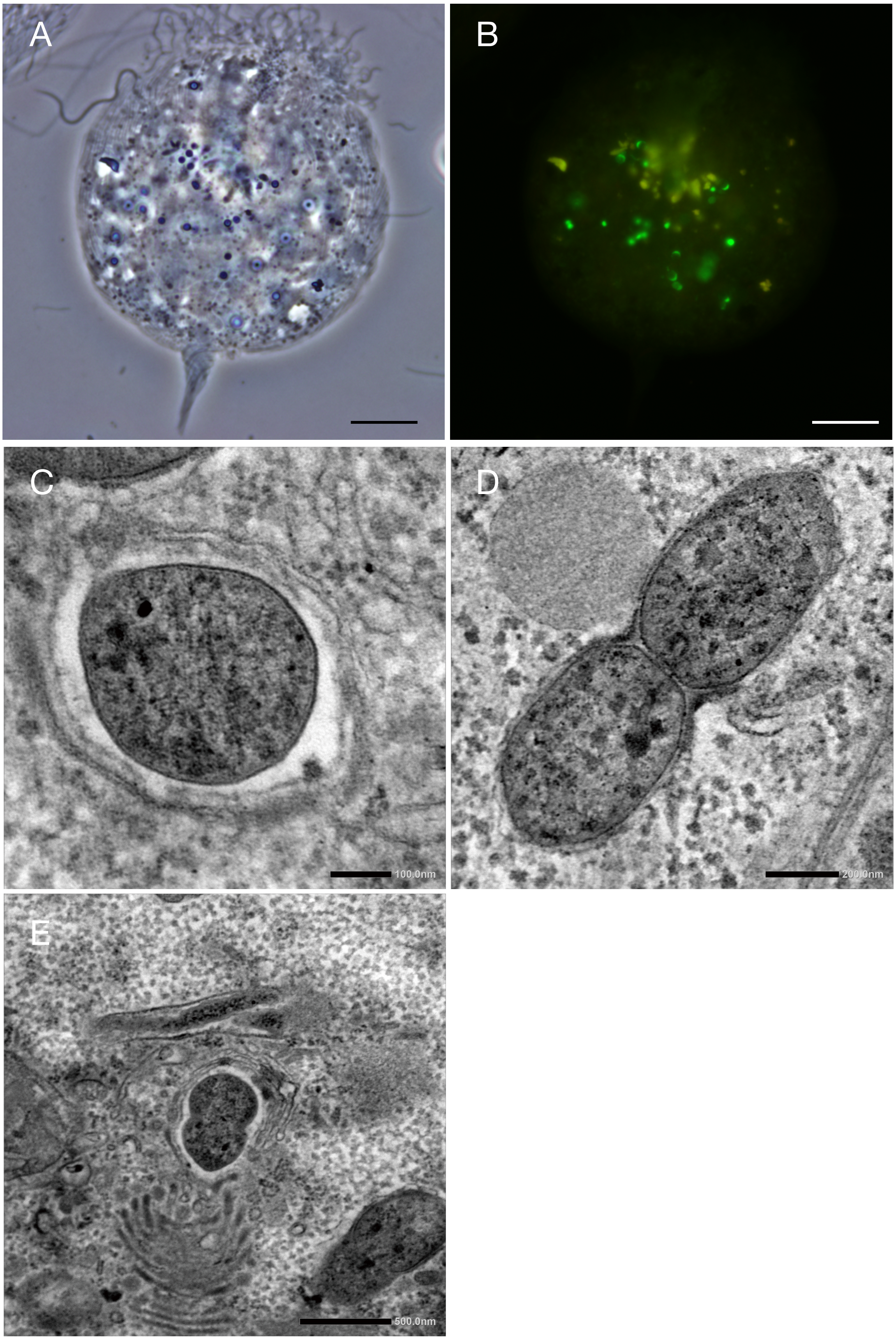

Figure S1. Fluorescence in situ hybridization analysis of 16S rRNA phylotype NkDv07 (**A, B**) and transmission electron micrographs of intracellular bacterial symbionts of *Pseudotrichonympha grassii* (**C–E**). Phase contrast image of *Devescovina* sp. from the gut of *Neotermes sugioi* (A) and NkDv07 cells detected using 6FAM-labelled probe NkDv07-142 (green) as single cocci (**B**). Amorphous yellowish autofluorescence in B was emitted from wood particles ingested by *Devescovina* sp. Putative clostridial cell (**C**), and "*Candidatus* Azobacteroides pseudotrichonymphae" cells (**D**). In panel E, a putative clostridial cell apparently during fission was in the center and a "*Ca.* A. pseudotrichonymphae" cell in the lower right. Bars: **A** and **B**, 10 µm; **C**, 100 nm; **D**, 200 nm; **E**, 500 nm.

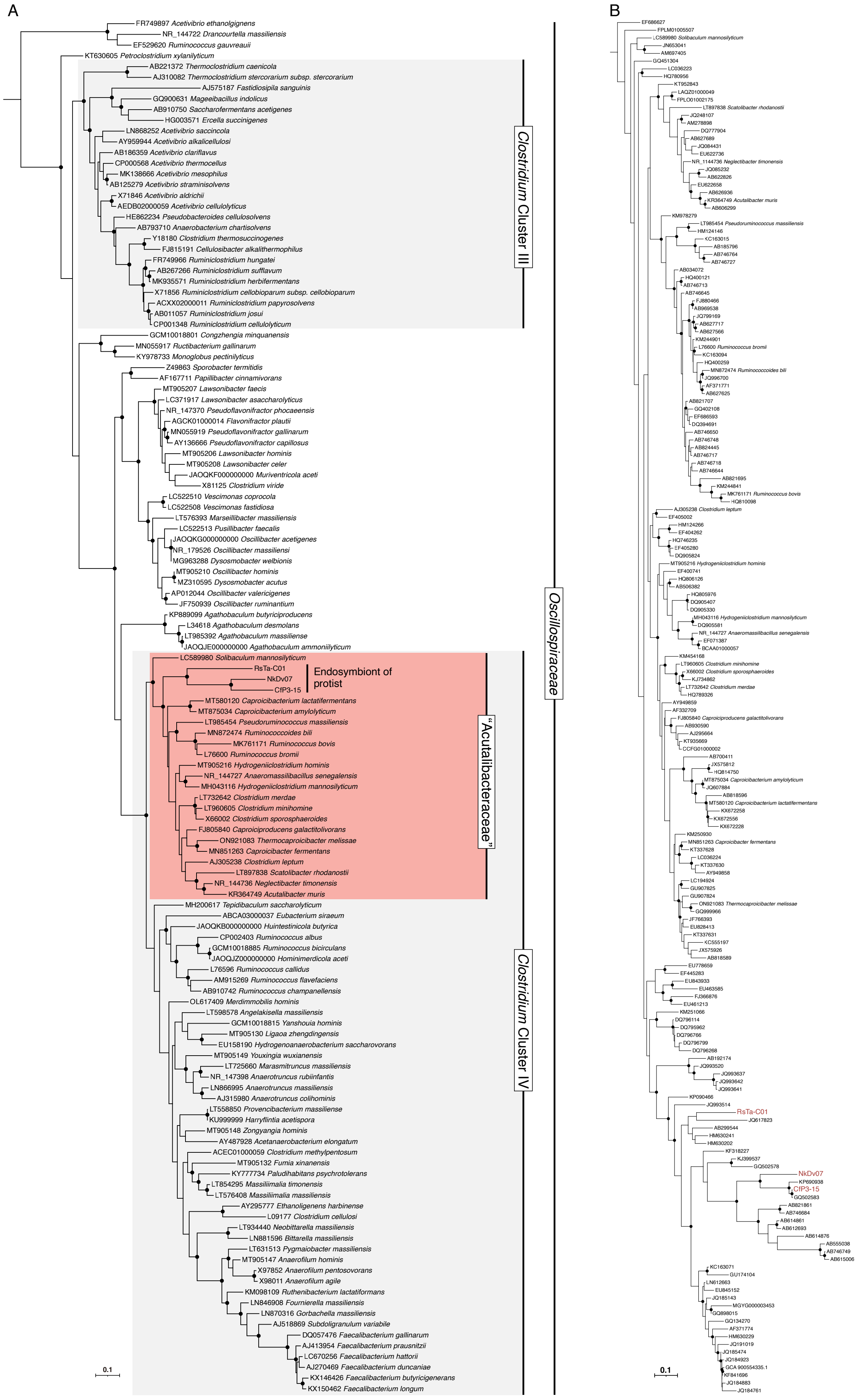

Figure S2. Phylogenetic positions of endosymbiotic *Clostridia* based on 16S rRNA gene sequences. (**A**) Maximum-likelihood tree of endosymbiotic *Clostridia* and other members of the family *Oscillospiraceae*. Described species of *Oscillospiraceae* were included in the tree. The tree was constructed with the GTR+I+G4 nucleotide substitution model and rooted with *Syntrophomonas wolfei* (CP000448) and *Thermosyntropha lipolytica* (FQWY01000001) as the outgroup. Clusters of *Clostridium* [60] and the clade corresponding to "Acutalibacteraceae" in the GTDB taxonomy are highlighted in gray and red, respectively. (**B**) Maximum-likelihood tree of endosymbiotic *Clostridia* and other members of the "Acutalibacteraceae". Uncultured clones closely related to described species and endosymbiotic *Clostridia* were included. The tree was constructed with the GTR+G4 nucleotide substitution model and rooted with *Sporobacter termitidis* (Z49863) and *Oscillibacter ruminantium* (JF750939) as the outgroup. Highly supported nodes (SH-aLRT value ≥ 80% and ultrafast bootstrap support value ≥ 95%) are highlighted with a closed circle.

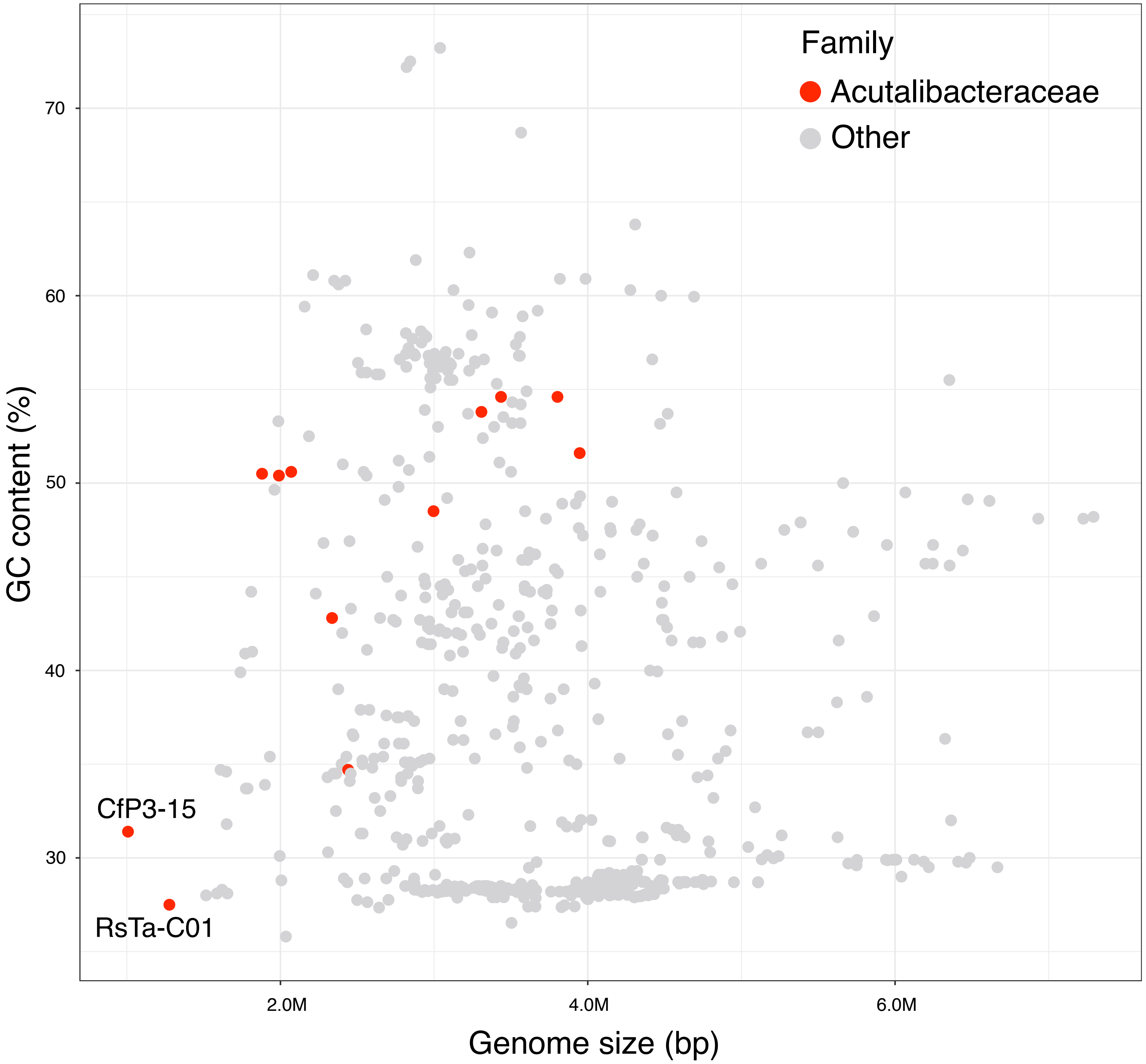

Figure S3. Relationships between the size and GC content of the complete genomes in the class *Clostridia*.

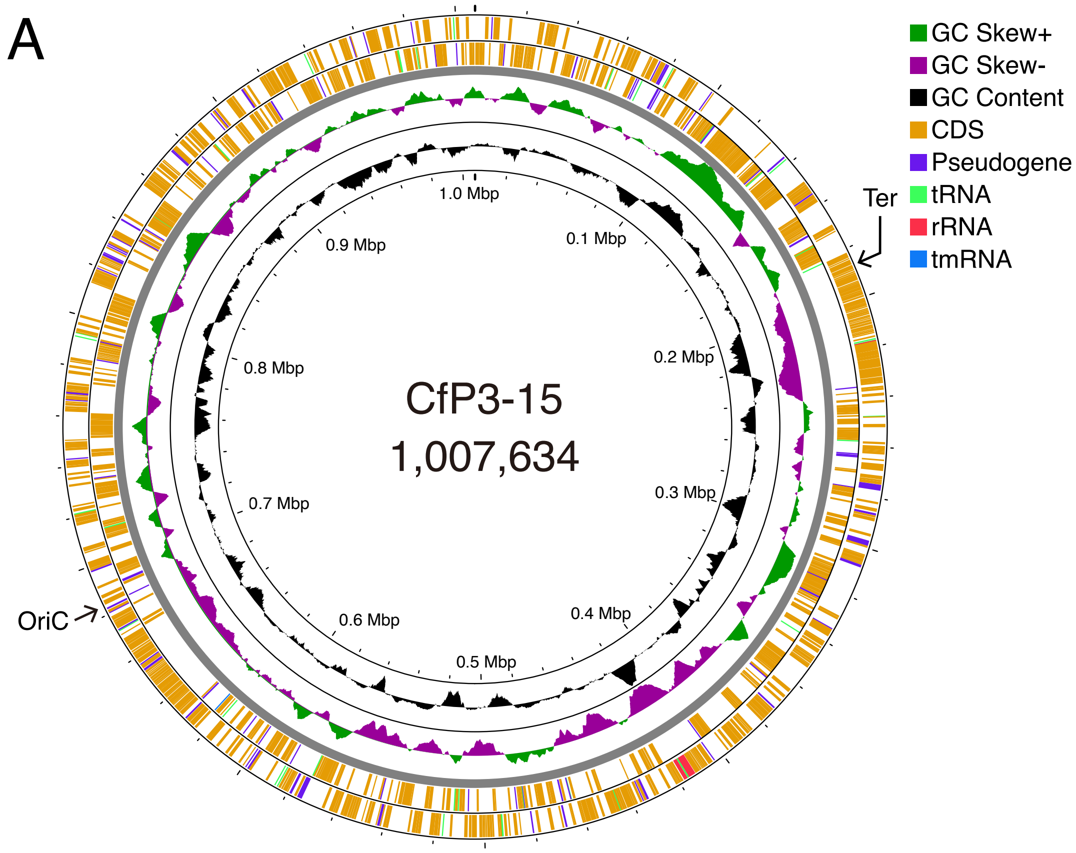

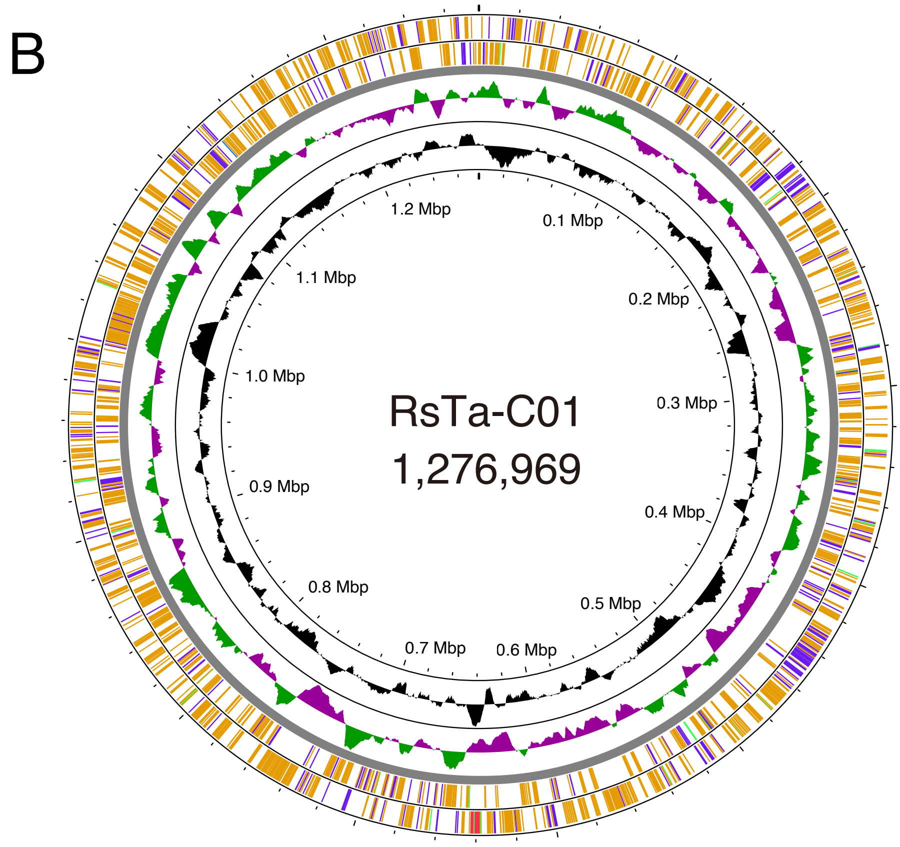

Figure S4. Circular representation of the CfP3-15 chromosome (**A**) and the RsTa-C01 chromosome (**B**). The rings denote the following features (from inside): (i) Scale in mega base pairs of the chromosome (black); (ii) GC content (black); (iii) GC skew (green and purple); (iv) predicted genes present on the forward strand (+); (v) genes present on the reverse strand (-). OriC and Ter sites are indicated by arrows in **A**. For the RsTa-C01 genome, we could not detect these sites, using Ori-Finder 2022.

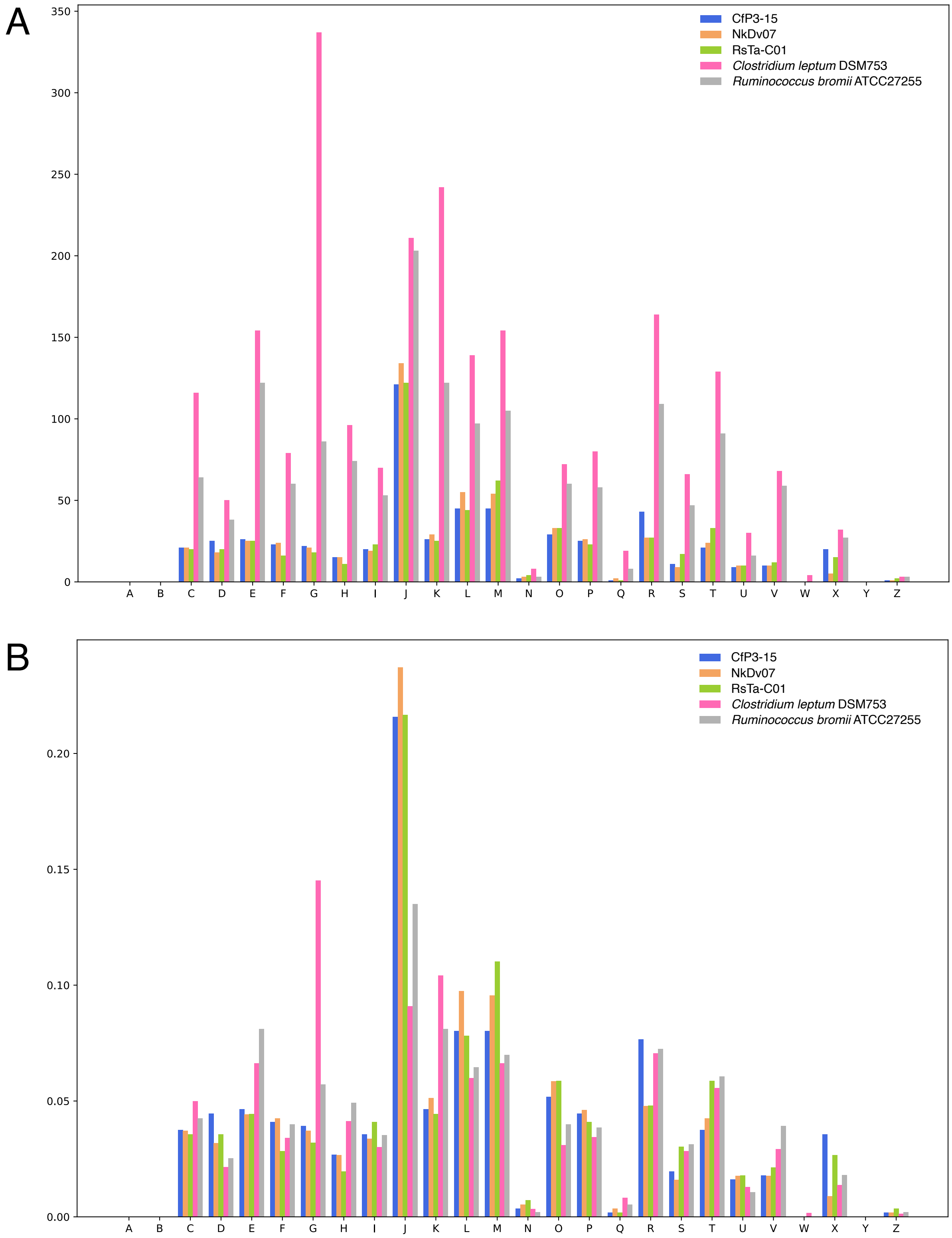

Figure S5. Comparison of the number (**A**) and ratio (**B**) of genes classified into clusters of orthologous genes (COG) functional categories. The genomes of CfP3-15, NkDv07, RsTa-C01, and their free-living relatives *Clostridium leptum* and *Ruminococcus bromii* were compared. The categories denote the following functions: (A) RNA processing and modification; (B) chromatin structure and dynamics; (C) energy production and conversion; (D) cell cycle control, cell division, chromosome partitioning; (E) amino acid transport and metabolism; (F) nucleotide transport and metabolism; (G) carbohydrate transport and metabolism; (H) coenzyme transport and metabolism; (I) lipid transport and metabolism; (J) translation, ribosomal structure and biogenesis; (K) transcription; (L) replication, recombination and repair; (M) cell wall/membrane/envelope biogenesis; (N) cell motility; (O) posttranslational modification, protein turnover, chaperones; (P) inorganic ion transport and metabolism; (Q) secondary metabolites biosynthesis, transport and catabolism; (R) general function prediction only; (S) function unknown; (T) signal transduction mechanisms; (U) intracellular trafficking, secretion, and vesicular transport; (V) defense mechanisms; (W) extracellular structures; (X) mobilome: prophages, transposons; (Y) nuclear structure; (Z) cytoskeleton.

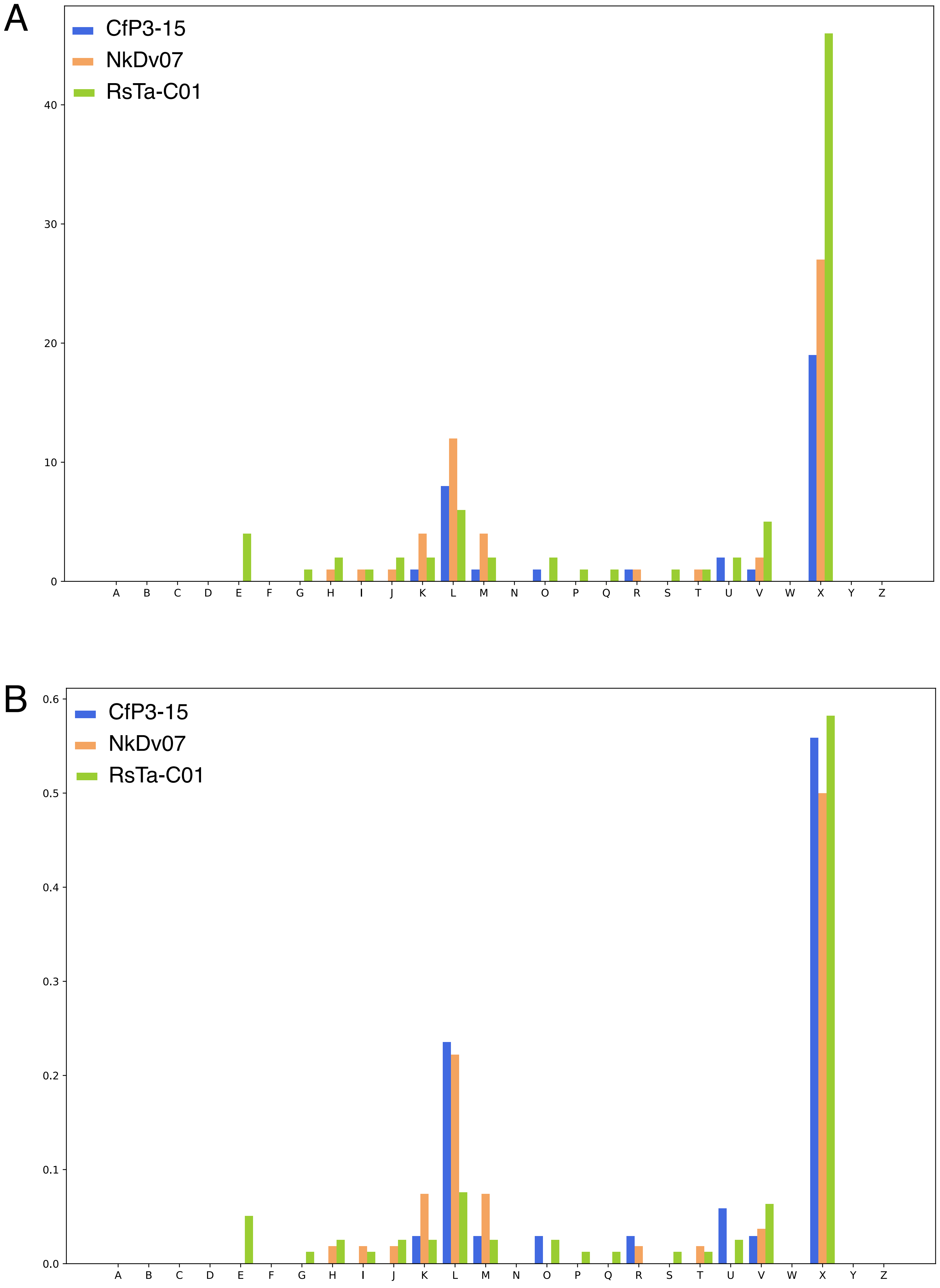

Figure S6. Comparison of the number (**A**) and ratio (**B**) of pseudogenes classified into clusters of orthologous genes (COG) functional categories. See also the legend to Fig. S5.

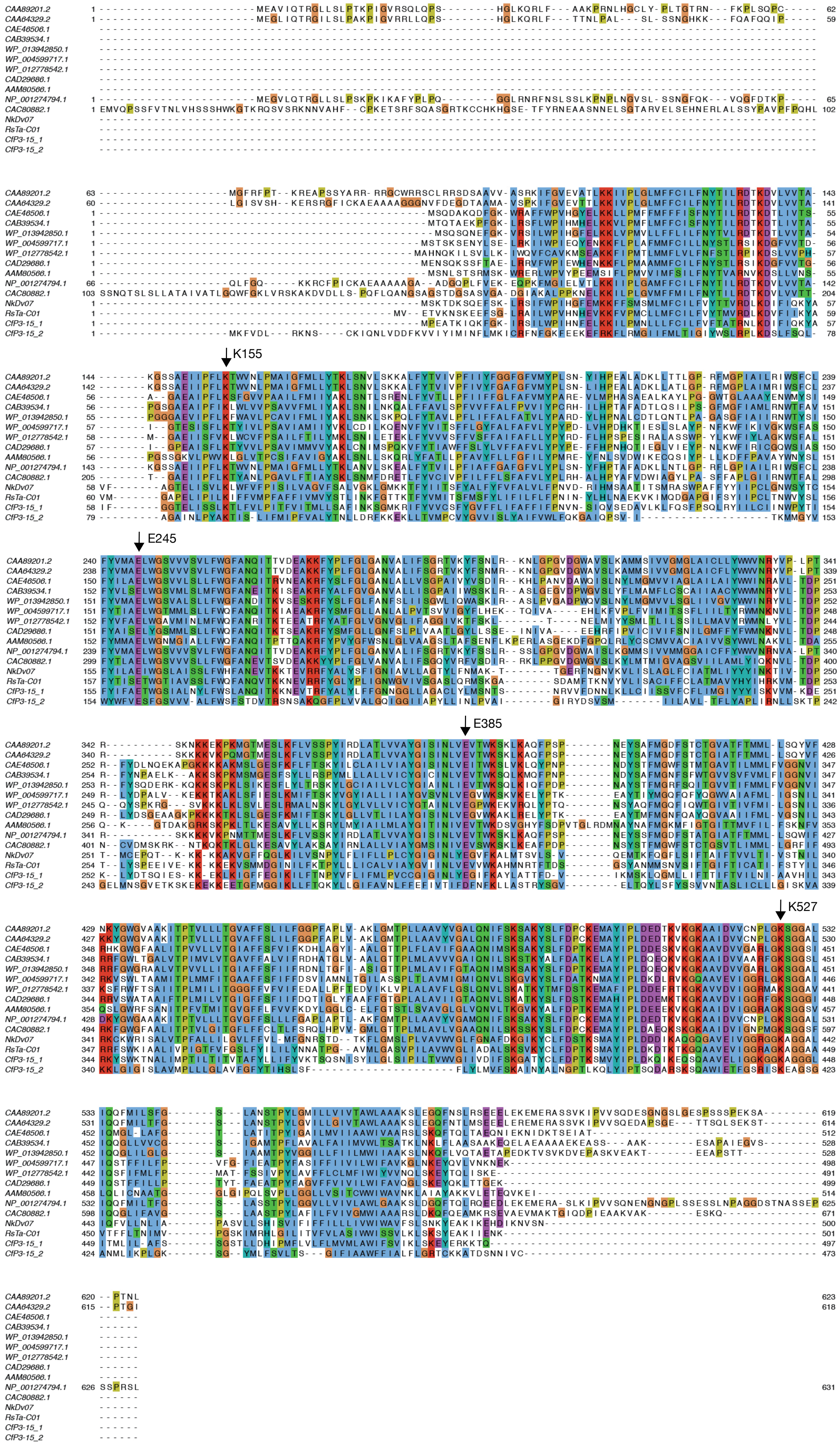

Figure S7. Amino acid sequence alignment of ATP/ADP translocase and other nucleotide transport proteins from the endosymbiotic *Clostridia*, other parasitic bacteria, and plastids. Conserved residues important for substrate specificity, transport efficiency, and counter exchange of ATP/ADP translocase (CAA89201.2) of the plastid of *Arabidopsis thaliana* are indicated with arrows.

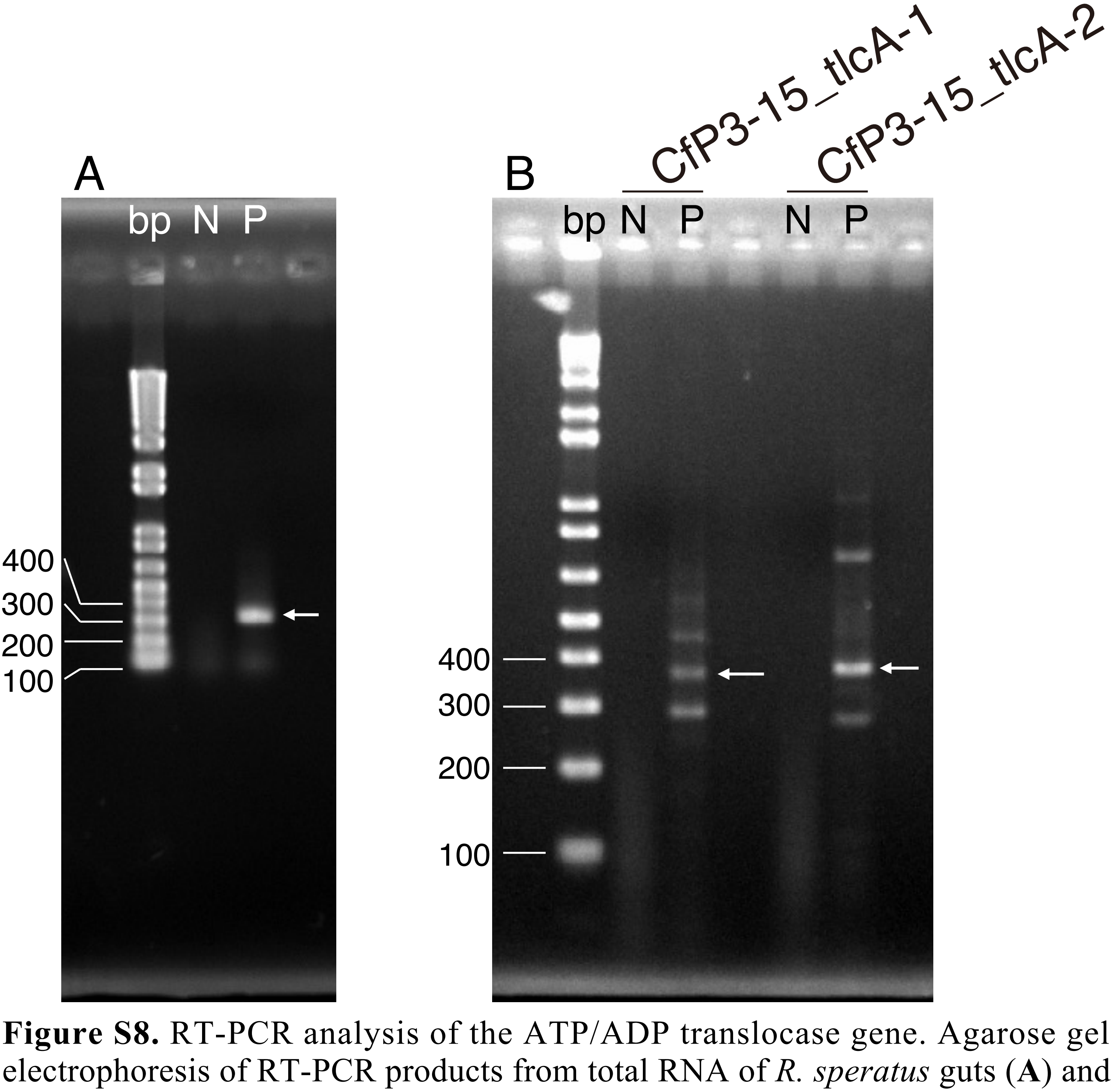

Figure S8. RT-PCR analysis of the ATP/ADP translocase gene. Agarose gel electrophoresis of RT-PCR products from total RNA of *R. speratus* guts (**A**) and *C. formosanus* guts (**B**). N, negative control (RNA samples without reverse transcription); P, products of RT-PCR using primer sets specific to the respective ATP/ADP translocase genes (see Table S2). These bands were excised from gels, and their sequences were verified by cloning and Sanger sequencing analysis.

A

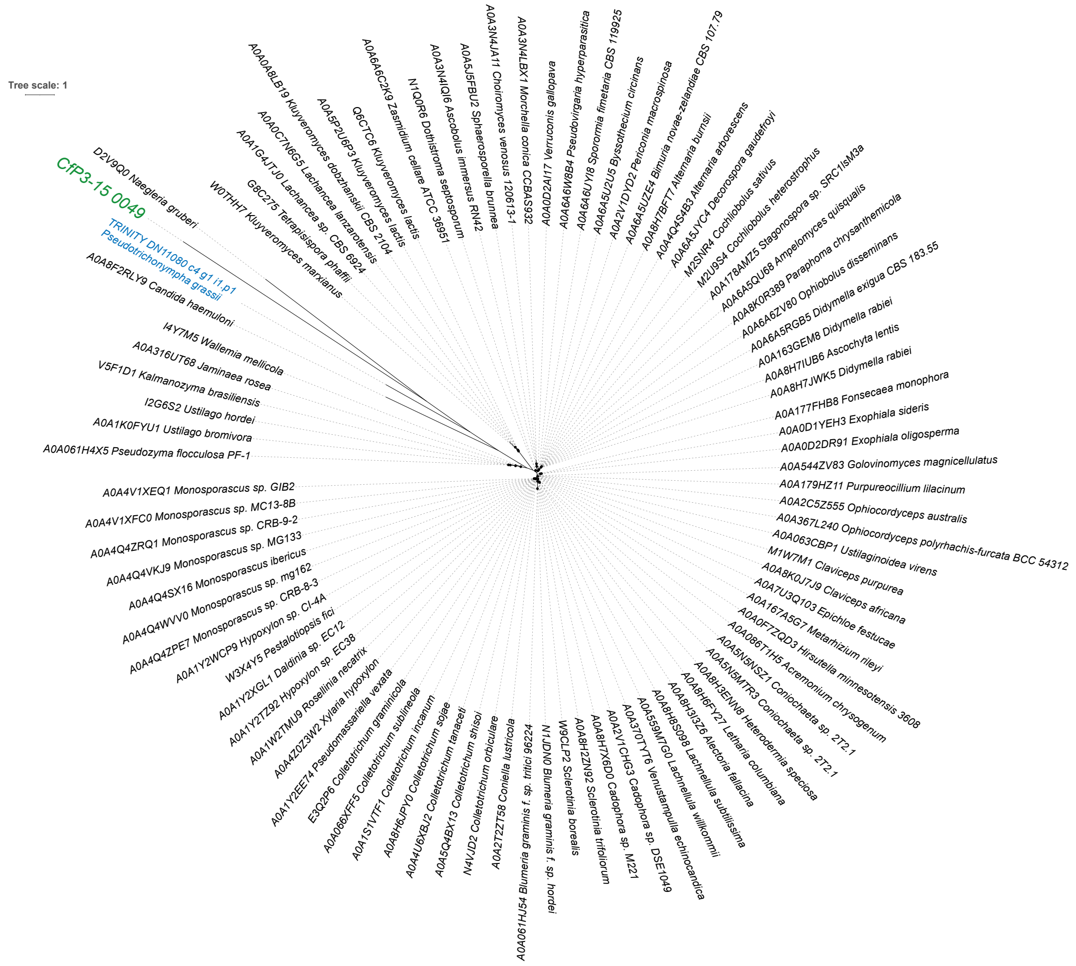

Tree scale: 1
CfP3-15 0049
D2V9Q0 Naegleria gruberi
TRINITY DN11080 c4 g1 i1.p1 Pseudotrichonympha grassii
A0A8F2RLY9 Candida haemuloni

B

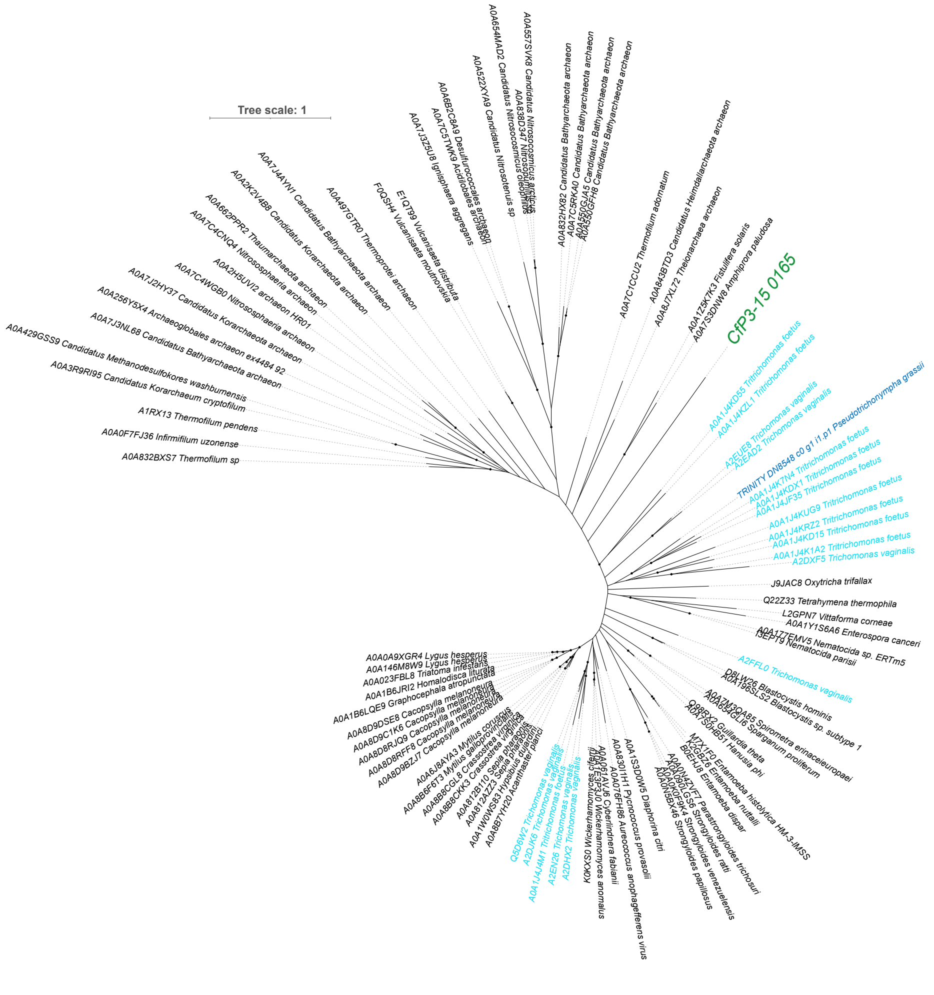

Tree scale: 1
CfP3-15 0165
A2FFL0 Trichomonas vaginalis
J9JAC8 Oxytricha trifallax
Q22Z33 Tetrahymena thermophila
L2GPN7 Vittaforma corneae
A0A1Y1S6A6 Enterospora canceri

C

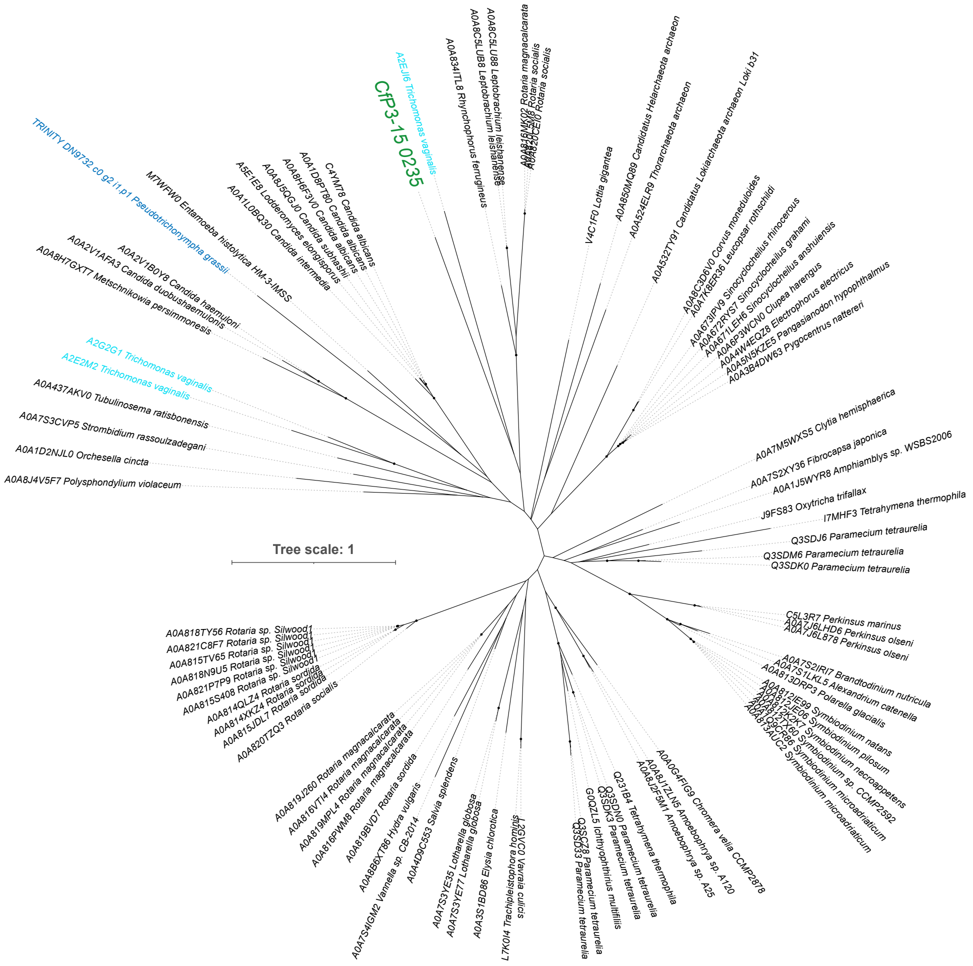

CfP3-15 0235
A2EJI6 Trichomonas vaginalis
A0A834ITL8 Rhynchophorus ferrugineus
V4C1F0 Lottia gigantea
A0A850MQ89 Candidatus Heliarchaeota archaeon
A0A524ELR9 Thorarchaeota archaeon
A0A532TY91 Candidatus Lokiarchaeota archaeon Loki b31
TRINITY DN9732 c0 g2 i1.p1 Pseudotrichonympha grassii
M7WFW0 Entamoeba histolytica HM-3:IMSS
A0A2V1B0Y8 Candida haemuloni
A0A2V1AFA3 Candida duobushaemulonis
A0A8H7GXT7 Metschnikowia persimmonesis
A2G2G1 Trichomonas vaginalis
A2E2M2 Trichomonas vaginalis
A0A437AKV0 Tubulinosema ratisbonensis
A0A7S3CVP5 Strombidium rassoulzadegani
A0A1D2NJL0 Orchesella cincta
A0A8J4V5F7 Polysphondylium violaceum
Tree scale: 1
A0A7M5WXS5 Clytia hemisphaerica
A0A7S2XY36 Fibrocapsa japonica
A0A1J5WYR8 Amphiamblys sp. WSBS2006
J9FS83 Oxytricha trifallax
I7MHF3 Tetrahymena thermophila
Q3SDJ6 Paramecium tetraurelia
Q3SDM6 Paramecium tetraurelia
Q3SDK0 Paramecium tetraurelia
C5L3R7 Perkinsus marinus
A0A7J6LHD6 Perkinsus olseni
A0A7J6L878 Perkinsus olseni
A0A7S2IRI7 Brandtodinium nutricula
A0A7S1LKL5 Alexandrium catenella
A0A813DRP3 Polarella glacialis
A0A0G4FIG9 Chromera velia CCMP2878
A0A819J260 Rotaria magnacalcarata
A0A819BVD7 Rotaria sordida
A0A8B6XT86 Hydra vulgaris
A0A7S4GM2 Vannella sp. CB-2014
A0A4D9C553 Salvia splendens
A0A7S3YE35 Lotharella globosa
A0A7S3YE77 Lotharella globosa
A0A3S1BD86 Elysia chlorotica
L7K0I4 Trachipleistophora hominis
G0QZL5 Ichthyophthirius multifiliis
Q23IB4 Tetrahymena thermophila
A0A818TY56 Rotaria sp. Silwood1
A0A821C8F7 Rotaria sp. Silwood1
A0A815TV65 Rotaria sp. Silwood1
A0A818N9U5 Rotaria sp. Silwood1
A0A821P7P9 Rotaria sp. Silwood1
A0A815S408 Rotaria sp. Silwood1
A0A814QLZ4 Rotaria sordida
A0A814XKZ4 Rotaria sordida
A0A815JDL7 Rotaria sordida
A0A820T7Q3 Rotaria socialis

D

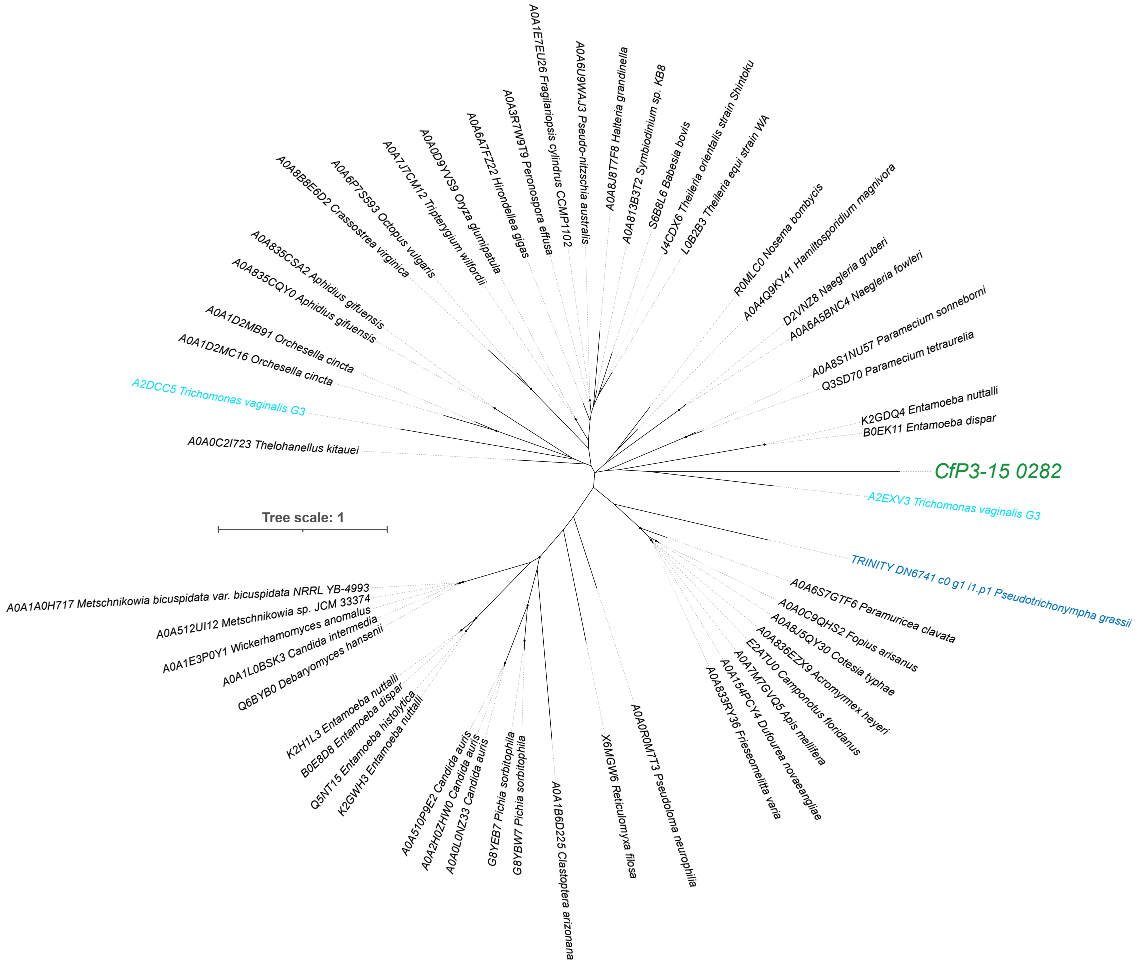

A0A1E7EU26 Fragilariopsis cylindrus CCMP1102
A0A6U9WAJ3 Pseudo-nitzschia australis
A0A8J8T7F8 Halteria grandinella
A0A813B3T2 Symbiodinium sp. KB8
S6BBL6 Babesia bovis
J4CDX6 Theileria orientalis strain Shintoku
L0B2B3 Theileria equi strain WA
A0A3R7W9T9 Peronospora effusa
A0A6A7FZ22 Hirondellea gigas
A0A0D9VVS9 Oryza glumipatula
A0A7J7CM12 Tripterygium wilfordii
A0A6P7S593 Octopus vulgaris
A0A8B8E6D2 Crassostrea virginica
A0A835CSA2 Aphidius gifuensis
A0A835CQY0 Aphidius gifuensis
A0A1D2MB91 Orchesella cincta
A0A1D2MC16 Orchesella cincta
A2DCC5 Trichomonas vaginalis G3
A0A0C2I723 Thelohanellus kitauei
R0MLC0 Nosema bombycis
A0A4Q9KY41 Hamiltosporidium magnivora
D2VNZ8 Naegleria gruberi
A0A6A5BNC4 Naegleria fowleri
A0A8S1NU57 Paramecium sonneborni
Q3SD70 Paramecium tetraurelia
K2GDQ4 Entamoeba nuttalli
B0EK11 Entamoeba dispar
CfP3-15 0282
A2EXV3 Trichomonas vaginalis G3
Tree scale: 1
TRINITY DN6741 c0 g1 i1.p1 Pseudotrichonympha grassii
A0A6S7GTF6 Paramuricea clavata
A0A0C9QHS2 Fopius arisanus
A0A8J5QY30 Cotesia typhae
A0A836EZX9 Acromyrmex heyeri
E2ATU0 Camponotus floridanus
A0A7M7GVQ5 Apis mellifera
A0A154PCY4 Dufourea novaeangliae
A0A833RY36 Frieseomelitta varia
A0A1A0H717 Metschnikowia bicuspidata var. bicuspidata NRRL YB-4993
A0A512UI12 Metschnikowia sp. JCM 33374
A0A1E3P0Y1 Wickerhamomyces anomalus
A0A1L0BSK3 Candida intermedia
Q6BYB0 Debaryomyces hansenii
K2H1L3 Entamoeba nuttalli
B0E8D8 Entamoeba dispar
Q5NT15 Entamoeba histolytica
K2GWH3 Entamoeba nuttalli
A0A510P9E2 Candida auris
A0A2H0ZHW0 Candida auris
A0A0L0NZ33 Candida auris
G8YEB7 Pichia sorbitophila
G8YBW7 Pichia sorbitophila
A0A1B6D225 Clastoptera arizonana
X6MGW6 Reticulomyxa filosa
A0A0R0M7T3 Pseudoloma neurophilia

E

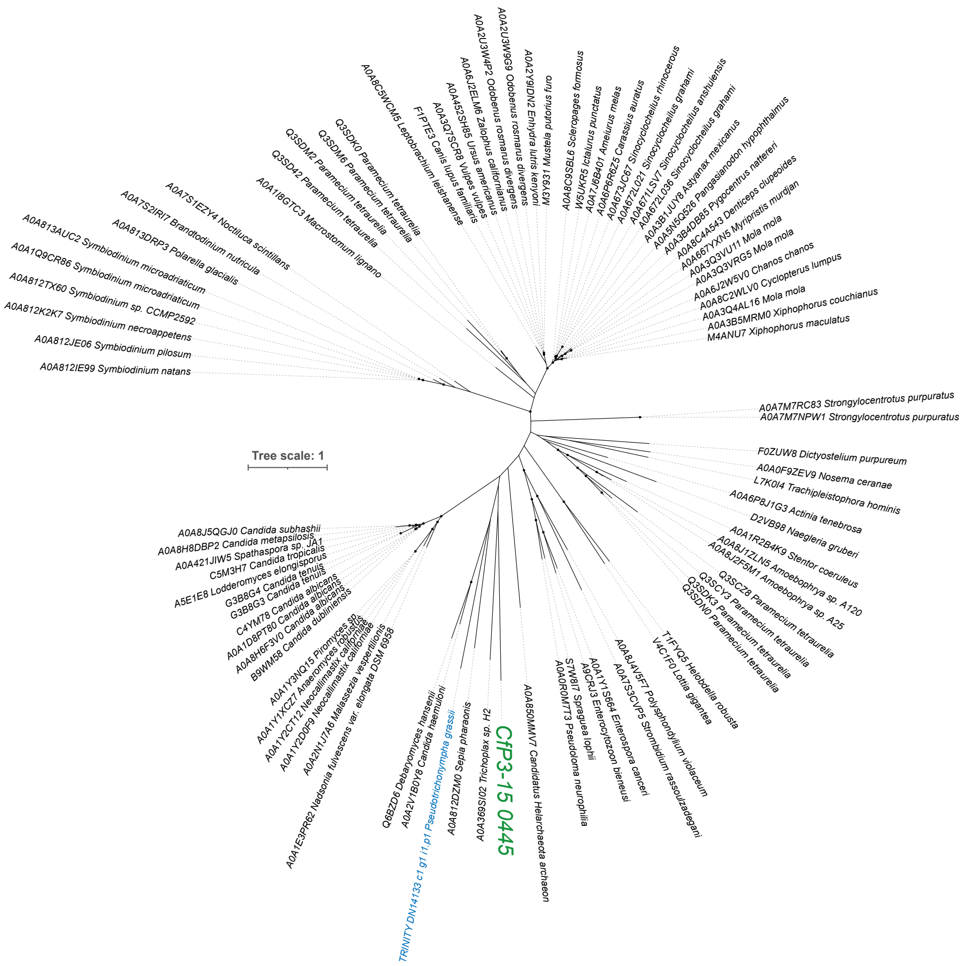
Tree scale: 1
CfP3-15 0445
TRINITY DN14133 c1 g1 i1 p1 Pseudotrichonympha grassii
A0A7M7RC83 Strongylocentrotus purpuratus
A0A7M7NPW1 Strongylocentrotus purpuratus
F0ZUW8 Dictyostelium purpureum
A0A0F9ZEV9 Nosema ceranae
L7K0I4 Trachipleistophora hominis
A0A6P8J1G3 Actinia tenebrosa
D2VB98 Naegleria gruberi
A0A1R2B4K9 Stentor coeruleus
A0A812IE99 Symbiodinium natans
A0A812JE06 Symbiodinium pilosum
A0A812K2K7 Symbiodinium necroappetens
A0A812TX60 Symbiodinium sp. CCMP2592
A0A1Q9CR86 Symbiodinium microadriaticum
A0A813AUC2 Symbiodinium microadriaticum
A0A813DRP3 Polarella glacialis
A0A7S2IRI7 Brandtodinium nutricula
A0A7S1EZY4 Noctiluca scintillans
A0A1I8GTC3 Macrostomum lignano
A0A8J5QGJ0 Candida subhashii
A0A8H8DBP2 Candida metapsilosis
A0A421JIW5 Spathaspora sp. JA1
C5M3H7 Candida tropicalis
A5E1E8 Lodderomyces elongisporus
Q6BZD6 Debaryomyces hansenii
A0A2V1B0Y8 Candida haemuloni
A0A812DZM0 Sepia pharaonis
A0A369SI02 Trichoplax sp. H2
A0A850MMV7 Candidatus Helarchaeota archaeon
A0A0R0M7T3 Pseudoloma neurophilia
S7W8I7 Spraguea lophii
A0A1Y3S664 Enterospora canceri
A0A7S3CVP5 Strombidium rassoulzadegani
T1FYQ5 Helobdella robusta
V4C1F0 Lottia gigantea
M4ANU7 Xiphophorus maculatus
A0A3B5MRM0 Xiphophorus couchianus
A0A3Q4AL16 Mola mola
A0A8C2WLV0 Cyclopterus lumpus
A0A3Q3VRG5 Mola mola
A0A667YXN5 Myripristis murdjan
A0A8C9SBL6 Scleropages formosus
W5UKR5 Ictalurus punctatus
A0A2Y9IDN2 Enhydra lutris kenyoni
A0A2U3W4P2 Odobenus rosmarus divergens
A0A2U3W9G9 Odobenus rosmarus divergens
F1PTE3 Canis lupus familiaris

F

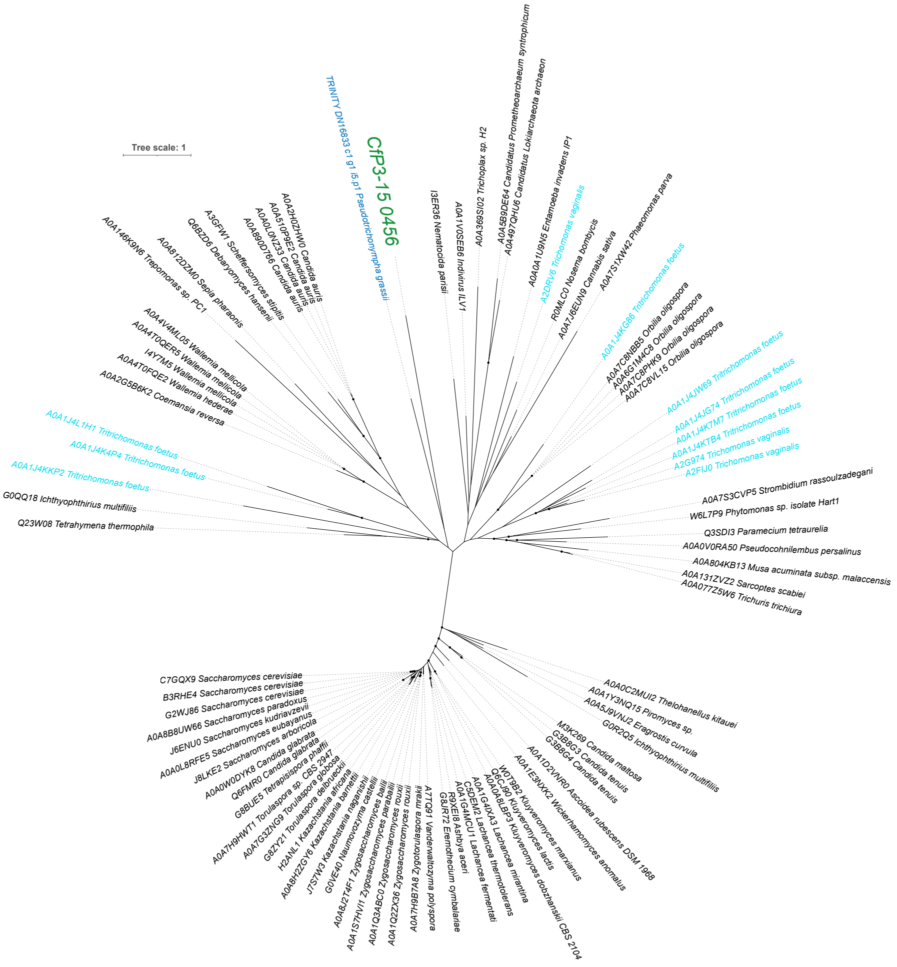
Tree scale: 1
CfP3-15 0456
TRINITY DN16833 c1 g1 i5 p1 Pseudotrichonympha grassii
A0A2N3DE64 Candidatus Prometheoarchaeum syntrophicum
A0A497QHU6 Candidatus Lokiarchaeota archaeon
A0A369SI02 Trichoplax sp. H2
A0A1V0SEB6 Indivirus ILV1
I3ER36 Nematocida parisii
A0A146K8N6 Trepomonas sp. PC1
A0A812DZM0 Sepia pharaonis
Q6BZD6 Debaryomyces hansenii
A0A1J4L1H1 Tritrichomonas foetus
A0A1J4K4P4 Tritrichomonas foetus
A0A1J4KKP2 Tritrichomonas foetus
G0QQ18 Ichthyophthirius multifiliis
Q23W08 Tetrahymena thermophila
A0A2G5B6K2 Coemansia reversa
A0A7S3CVP5 Strombidium rassoulzadegani
W6L7P9 Phytomonas sp. isolate Hart1
Q3SDI3 Paramecium tetraurelia
A0A0V0RA50 Pseudocohnilembus persalinus
A0A804KB13 Musa acuminata subsp. malaccensis
A0A131ZVZ2 Sarcoptes scabiei
A0A077Z5W6 Trichuris trichiura
A0A0C2MUI2 Thelohanellus kitauei
A0A1Y3NQ15 Piromyces sp.
C7GQX9 Saccharomyces cerevisiae
B3RHE4 Saccharomyces cerevisiae
G2WJ86 Saccharomyces cerevisiae
A0A8B8UW66 Saccharomyces paradoxus
J6ENU0 Saccharomyces kudriavzevii

G

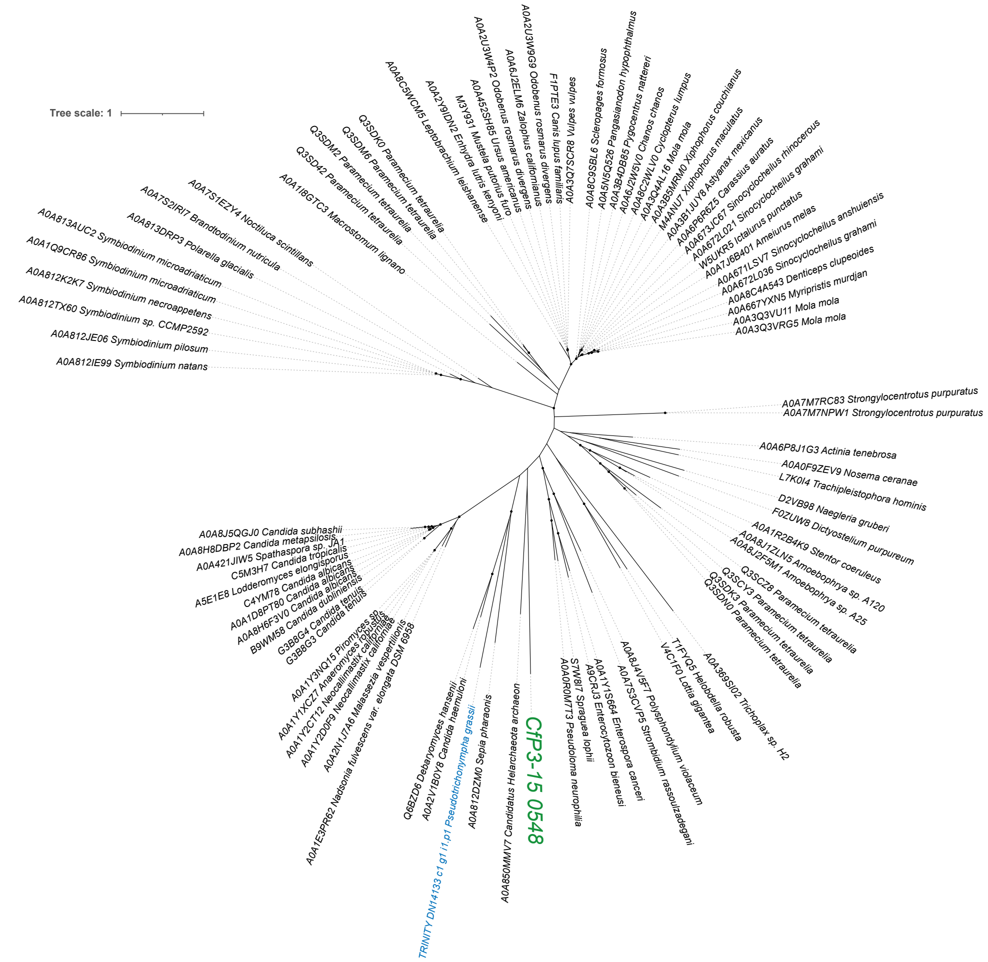
Tree scale: 1
A0A812IE99 Symbiodinium natans
A0A812JE06 Symbiodinium pilosum
A0A812TX60 Symbiodinium sp. CCMP2592
A0A812K2K7 Symbiodinium necroappetens
A0A1Q9CR86 Symbiodinium microadriaticum
A0A813AUC2 Symbiodinium microadriaticum
A0A813DRP3 Polarella glacialis
A0A7S2IRI7 Brandtodinium nutricula
A0A7S1EZY4 Noctiluca scintillans
A0A1I8GTC3 Macrostomum lignano
Q3SD42 Paramecium tetraurelia
Q3SDM2 Paramecium tetraurelia
Q3SDM6 Paramecium tetraurelia
Q3SDK0 Paramecium tetraurelia
A0A8C5WCM5 Leptobrachium leishanense
A0A2Y9DN2 Enhydra lutris kenyoni
M3Y931 Mustela putorius furo
A0A452SH85 Ursus americanus
A0A2U3W4P2 Odobenus rosmarus divergens
A0A6J2ELM6 Zalophus californianus
A0A2U3W9G9 Odobenus rosmarus divergens
F1PTE3 Canis lupus familiaris
A0A3Q7SCR8 Vulpes vulpes
A0A8C9SBL6 Scleropages formosus
A0A5N5Q526 Pangasianodon hypophthalmus
A0A3B4DB65 Pygocentrus nattereri
A0A6J2WLV0 Chanos chanos
A0A8C2WLV0 Cyclopterus lumpus
A0A3Q4AL16 Mola mola
A0A3B5MRM0 Xiphophorus couchianus
A0A3B1JJY8 Astyanax mexicanus
A0A6P6R625 Carassius auratus
A0A672L021 Sinocyclocheilus rhinocerous
A0A673JC67 Sinocyclocheilus grahami
W5UKR5 Ictalurus punctatus
A0A7J6B401 Ameiurus melas
A0A671LSV7 Sinocyclocheilus anshuiensis
A0A672L036 Sinocyclocheilus grahami
A0A8C4A543 Denticeps clupeoides
A0A667YXN5 Myripristis murdjan
A0A3Q3VU11 Mola mola
A0A3Q3VRG5 Mola mola
A0A7M7RC83 Strongylocentrotus purpuratus
A0A7M7NPW1 Strongylocentrotus purpuratus
A0A6P8J1G3 Actinia tenebrosa
A0A0F9ZEV9 Nosema ceranae
L7K0I4 Trachipleistophora hominis
D2VB98 Naegleria gruberi
F0ZUW8 Dictyostelium purpureum
A0A1R2B4K9 Stentor coeruleus
A0A8J1ZLN5 Amoebophrya sp. A120
A0A8J2F5M1 Amoebophrya sp. A25
Q3SCZ8 Paramecium tetraurelia
Q3SCY3 Paramecium tetraurelia
Q3SDK3 Paramecium tetraurelia
Q3SDN0 Paramecium tetraurelia
A0A369SI02 Trichoplax sp. H2
T1FYQ5 Helobdella robusta
V4C1F0 Lottia gigantea
A0A8J4V5F7 Polysphondylium violaceum
A0A7S3CVP5 Strombidium rassoulzadegani
A0A1Y1S664 Enterospora canceri
A9CRJ3 Enterocytozoon bieneusi
S7W8I7 Spraguea lophii
A0A0R0M7T3 Pseudoloma neurophilia
CfP3-15 0548
A0A850MMV7 Candidatus Heliarchaeota archaeon
A0A812DZM0 Sepia pharaonis
TRINITY DN14133 c1 g1 i1.p1 Pseudotrichonympha grassii
A0A2V1B0Y6 Candida haemulonii
Q6BZD6 Debaryomyces hansenii
A0A1E3PR62 Nadsonia fulvescens var. elongata DSM 6958
A0A2N1J7A6 Malassezia vespertilionis
A0A1Y2D0F9 Neocallimastix californiae
A0A1Y2CT12 Neocallimastix californiae
A0A1Y1XCZ7 Anaeromyces robustus
A0A1Y3NQ15 Piromyces sp.
G3B8G3 Candida tenuis
G3B8G4 Candida tenuis
B9WM58 Candida dubliniensis
A0A8H6F3V0 Candida albicans
A0A1D8PT80 Candida albicans
C4YM78 Candida albicans
A5E1E8 Lodderomyces elongisporus
C5M3H7 Candida tropicalis
A0A421JIW5 Spathaspora sp. JA1
A0A8H8DBP2 Candida metapsilosis
A0A8J5QGJ0 Candida subhashii

H

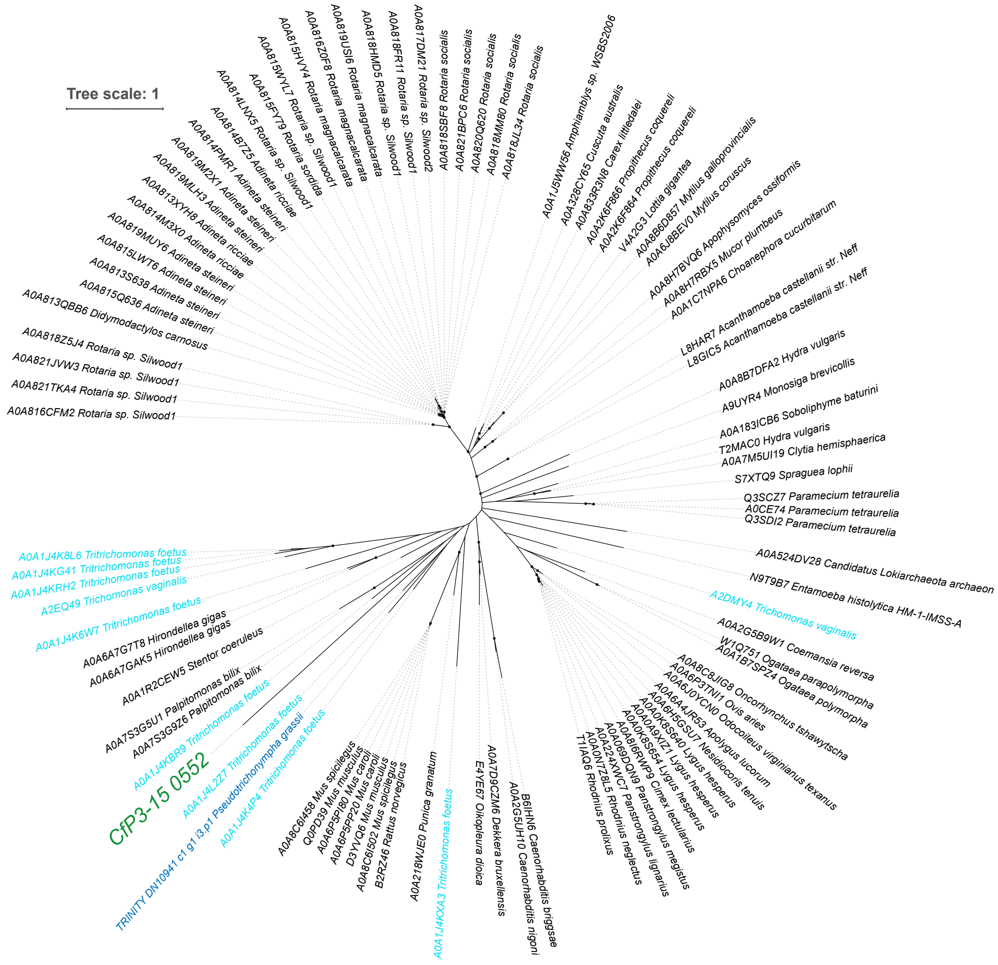
Tree scale: 1
A0A816CFM2 Rotaria sp. Silwood1
A0A821TKA4 Rotaria sp. Silwood1
A0A821JVW3 Rotaria sp. Silwood1
A0A818Z5J4 Rotaria sp. Silwood1
A0A813QBB6 Didymodactylos carnosus
A0A815Q636 Adineta steineri
A0A813S638 Adineta steineri
A0A815LWT6 Adineta steineri
A0A819MUY6 Adineta steineri
A0A814M3X0 Adineta ricciae
A0A813XYH8 Adineta steineri
A0A819MLH3 Adineta ricciae
A0A819M2X1 Adineta steineri
A0A814PMR1 Adineta steineri
A0A814BTZ5 Adineta ricciae
A0A814LNX5 Rotaria sp. Silwood1
A0A815FY79 Rotaria sordida
A0A815WYL7 Rotaria sp. Silwood1
A0A815HYV4 Rotaria magnacalcarata
A0A816Z0F8 Rotaria magnacalcarata
A0A819USI6 Rotaria magnacalcarata
A0A818HMD5 Rotaria sp. Silwood1
A0A818FR11 Rotaria sp. Silwood1
A0A817DM21 Rotaria sp. Silwood2
A0A818SBF8 Rotaria socialis
A0A821BPC6 Rotaria socialis
A0A820Q620 Rotaria socialis
A0A818MM80 Rotaria socialis
A0A818JL34 Rotaria socialis
A0A1J5WW56 Amphiamblys sp. WSBS2006
A0A328CY65 Cuscuta australis
A0A833R3N8 Carex littledalei
A0A2K6F866 Propithecus coquereli
A0A2K6F864 Propithecus coquereli
V4A2G3 Lottia gigantea
A0A8B6D857 Mytilus galloprovincialis
A0A6J8BEV0 Mytilus coruscus
A0A8H7BVQ6 Apophysomyces ossiformis
A0A8H7RBX5 Mucor plumbeus
A0A1C7NPA6 Choanephora cucurbitarum
L8HAR7 Acanthamoeba castellanii str. Neff
L8GIC5 Acanthamoeba castellanii str. Neff
A0A8B7DFA2 Hydra vulgaris
A9UYR4 Monosiga brevicollis
A0A183ICB6 Soboliphyme baturini
T2MAC0 Hydra vulgaris
A0A7M5UI19 Clytia hemisphaerica
S7XTQ9 Spraguea lophii
Q3SCZ7 Paramecium tetraurelia
A0CE74 Paramecium tetraurelia
Q3SDI2 Paramecium tetraurelia
A0A524DV28 Candidatus Lokiarchaeota archaeon
N9T9B7 Entamoeba histolytica HM-1-IMSS-A
A2DMY4 Trichomonas vaginalis
A0A2G5B9W1 Coemansia reversa
W1Q751 Ogataea parapolymorpha
A0A1B7SFZ4 Ogataea polymorpha
A0A8C8JIG8 Oncorhynchus tshawytscha
A0A6P3TNI1 Ovis aries
A0A6J0YCN0 Odocoileus virginianus texanus
A0A6A4JR53 Apolygus lucorum
A0A0K8SGU7 Nesidiocoris tenuis
A0A6H5GSV7 Lygus hesperus
A0A0A9XT21 Lygus hesperus
A0A0K8S654 Cimex lectularius
A0A8I6RWP9 Panstrongylus megistus
A0A069DQN9 Panstrongylus lignarius
A0A224XWC7 Rhodnius neglectus
A0A0N7Z8L5 Rhodnius prolixus
T1IAQ6 Rhodnius prolixus
B6IHN6 Caenorhabditis briggsae
A0A2G5UH10 Caenorhabditis nigoni
A0A7D9CZM6 Dekkera bruxellensis
E4YE67 Oikopleura dioica
A0A1J4KXA3 Tritrichomonas foetus
A0A218WJE0 Punica granatum
B2RZ46 Rattus norvegicus
A0A8C6I502 Mus spicilegus
D3YVQ6 Mus musculus
A0A6P5PF20 Mus caroli
A0A6P5PI80 Mus caroli
Q0PD39 Mus musculus
A0A8C6I458 Mus spicilegus
A0A1J4K4P4 Tritrichomonas foetus
TRINITY DN10941 c1 g1 i3.p1 Pseudotrichonympha grassii
A0A1J4L2Z7 Tritrichomonas foetus
CfP3-15 0552
A0A1J4KBR9 Tritrichomonas foetus
A0A7S3G9Z6 Palpitomonas bilix
A0A7S3G5U1 Palpitomonas bilix
A0A1R2CEW5 Stentor coeruleus
A0A6A7GAK5 Hirondellea gigas
A0A6A7G7T8 Hirondellea gigas
A0A1J4K6W7 Tritrichomonas foetus
A2EQ49 Trichomonas vaginalis
A0A1J4KRH2 Tritrichomonas foetus
A0A1J4KG41 Tritrichomonas foetus
A0A1J4K8L6 Tritrichomonas foetus

I

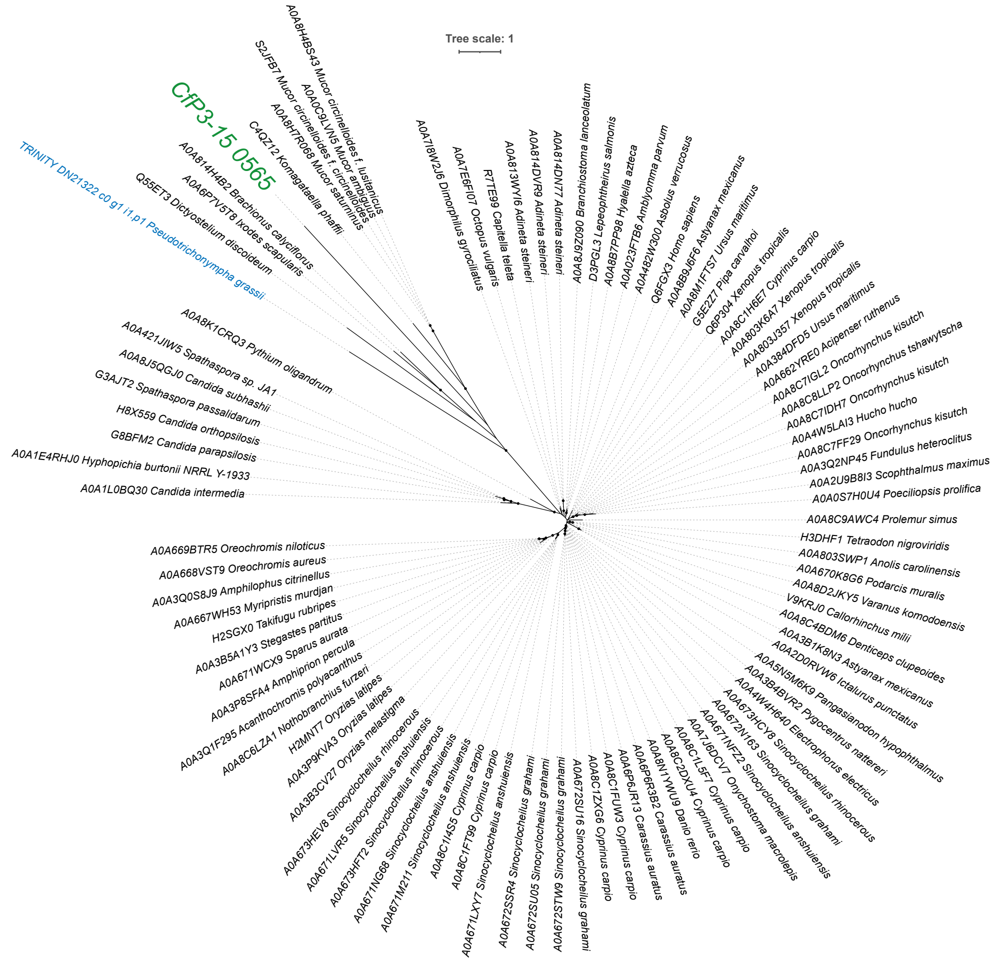
Tree scale: 1
CfP3-15 0565
TRINITY DN21322 c0 g1 i1.p1 Pseudotrichonympha grassii
A0A8H4BS43 Mucor circinelloides f. lusitanicus
A0A0C9LVN5 Mucor ambiguus
S2JFB7 Mucor circinelloides f. circinelloides
A0A8H7R068 Mucor saturninus
C4QZ12 Komagataella phaffii
A0A814H4B2 Brachionus calyciflorus
A0A6P7V5T8 Ixodes scapularis
Q55ET3 Dictyostelium discoideum
A0A8K1CRQ3 Pythium oligandrum
A0A421JIW5 Spathaspora sp. JA1
A0A8J5QGJ0 Candida subhashii
G3AJT2 Spathaspora passalidarum
H8X559 Candida orthopsilosis
G8BFM2 Candida parapsilosis
A0A1E4RHJ0 Hyphopichia burtonii NRRL Y-1933
A0A1L0BQ30 Candida intermedia
A0A7I8W2J6 Dimorphilus gyrociliatus
A0A7E6FI07 Octopus vulgaris
R7TE99 Capitella teleta
A0A813WYI6 Adineta steineri
A0A814DVR9 Adineta steineri
A0A814DN77 Adineta steineri
A0A8J9Z090 Branchiostoma lanceolatum
D3PGL3 Lepeophtheirus salmonis
A0A8B7PP98 Hyalella azteca
A0A023FTB6 Amblyomma parvum
A0A482W300 Asbolus verrucosus
Q6FGX3 Homo sapiens
A0A8B9J6F6 Astyanax mexicanus
A0A8M1FTS7 Ursus maritimus
G5E227 Pipa carvalhoi
Q6P304 Xenopus tropicalis
A0A8C1H6E7 Cyprinus carpio
A0A803K6A7 Xenopus tropicalis
A0A803J357 Xenopus tropicalis
A0A384DFD5 Ursus maritimus
A0A662YRE0 Acipenser ruthenus
A0A8C7IGL2 Oncorhynchus kisutch
A0A8C8LLP2 Oncorhynchus tshawytscha
A0A8C7IDH7 Oncorhynchus kisutch
A0A4W5LAI3 Hucho hucho
A0A8C7FF29 Oncorhynchus kisutch
A0A3Q2NP45 Fundulus heteroclitus
A0A2U9B8I3 Scophthalmus maximus
A0A0S7H0U4 Poeciliopsis prolifica
A0A8C9AWC4 Prolemur simus
H3DHF1 Tetraodon nigroviridis
A0A803SWP1 Anolis carolinensis
A0A670K8G6 Podarcis muralis
A0A8D2JKY5 Varanus komodoensis
V9KRJ0 Callorhinchus milii
A0A8C4BDM6 Denticeps clupeoides
A0A3B1K8N3 Astyanax mexicanus
A0A2D0RVW6 Ictalurus punctatus
A0A5N5M6K9 Pangasianodon hypophthalmus
A0A3B4BVR2 Pygocentrus nattereri
A0A4W4H640 Electrophorus electricus
A0A673HCY8 Sinocyclocheilus rhinocerous
A0A672N163 Sinocyclocheilus grahami
A0A671NFZ2 Sinocyclocheilus anshuiensis
A0A7J6DCV7 Onychostoma macrolepis
A0A8C1L5F7 Cyprinus carpio
A0A8C2DXU4 Cyprinus carpio
A0A8N1YWU9 Danio rerio
A0A6P6R3B2 Carassius auratus
A0A6P6JR13 Carassius auratus
A0A8C1FUW3 Cyprinus carpio
A0A8C1ZXG6 Cyprinus carpio
A0A672SU16 Sinocyclocheilus grahami
A0A672STM9 Sinocyclocheilus grahami
A0A672SU05 Sinocyclocheilus grahami
A0A672SSR4 Sinocyclocheilus grahami
A0A671LXY7 Sinocyclocheilus anshuiensis
A0A8C1FT99 Cyprinus carpio
A0A8C1I4S5 Cyprinus carpio
A0A671M211 Sinocyclocheilus anshuiensis
A0A671NG68 Sinocyclocheilus anshuiensis
A0A673HFT2 Sinocyclocheilus rhinocerous
A0A671LVR5 Sinocyclocheilus anshuiensis
A0A673HEV8 Sinocyclocheilus rhinocerous
A0A3B3CV27 Oryzias melastigma
A0A3P9KVA3 Oryzias latipes
H2MNT7 Oryzias latipes
A0A8C6LZA1 Nothobranchius furzeri
A0A3Q1F295 Acanthochromis polyacanthus
A0A3P8SFA4 Amphiprion percula
A0A671WCX9 Sparus aurata
A0A3B5A1Y3 Stegastes partitus
H2SGX0 Takifugu rubripes
A0A667WH53 Myripristis murdjan
A0A3Q0S8J9 Amphilophus citrinellus
A0A668VST9 Oreochromis aureus
A0A669BTR5 Oreochromis niloticus

J

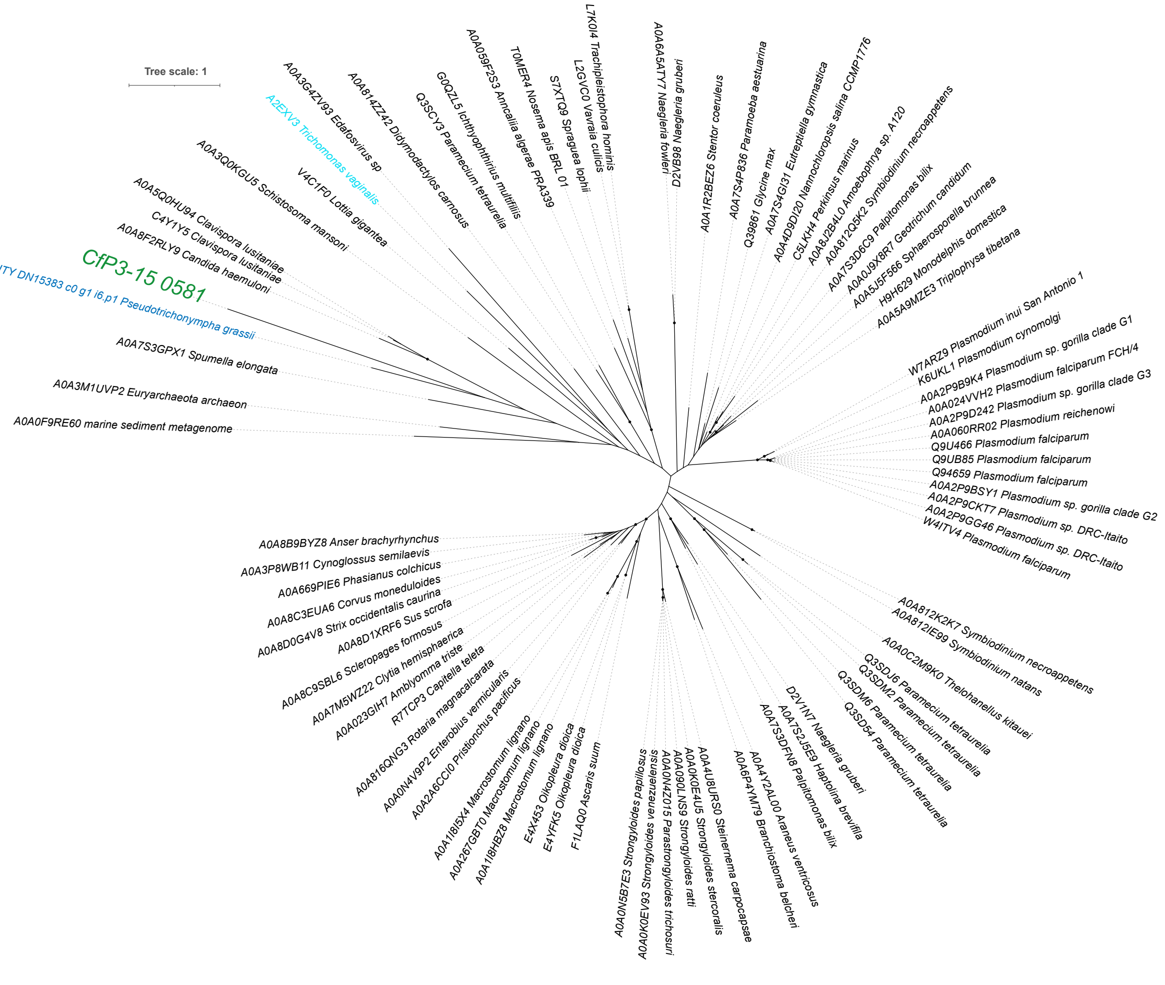
Tree scale: 1
CfP3-15 0581
TRINITY DN15383 c0 g1 i6.p1 Pseudotrichonympha grassii
A2EXV3 Trichomonas vaginalis
A0A3G4ZV93 Edafosvirus sp
A0A814ZZ42 Didymodactylos carnosus
A0A3Q0KGU5 Schistosoma mansoni
V4C1F0 Lottia gigantea
A0A5Q0HU94 Clavispora lusitaniae
C4Y1Y5 Clavispora lusitaniae
A0A8F2RLY9 Candida haemuloni
A0A7S3GPX1 Spumella elongata
A0A3M1UVP2 Euryarchaeota archaeon
A0A0F9RE60 marine sediment metagenome
Q3SCY3 Paramecium tetraurelia
G0QZL5 Ichthyophthirius multifiliis
A0A059F2S3 Anncaliia algerae PRA339
T0MER4 Nosema apis BRL 01
S7XTQ9 Spraguea lophii
L2GVC0 Vavraia culicis
L7K0I4 Trachipleistophora hominis
A0A6A5ATY7 Naegleria fowleri
D2VB98 Naegleria gruberi
A0A1R2BEZ6 Stentor coeruleus
A0A7S4P836 Paramoeba aestuarina
Q39861 Glycine max
A0A7S4GI31 Eutreptiella gymnastica
A0A4D9DI20 Nannochloropsis salina CCMP1776
C5LKH4 Perkinsus marinus
A0A8J2B4L0 Amoebophrya sp. A120
A0A812Q5K2 Symbiodinium necroappetens
A0A7S3D6C9 Palpitomonas bilix
A0A0J9X8R7 Geotrichum candidum
A0A5J5F566 Sphaerosporella brunnea
H9H629 Monodelphis domestica
A0A5A9MZE3 Triplophysa tibetana
W7ARZ9 Plasmodium inui San Antonio 1
K6UKL1 Plasmodium cynomolgi
A0A2P9B9K4 Plasmodium sp. gorilla clade G1
A0A024VVH2 Plasmodium falciparum FCH/4
A0A2P9D242 Plasmodium sp. gorilla clade G3
A0A060RR02 Plasmodium reichenowi
Q9U466 Plasmodium falciparum
Q9UB85 Plasmodium falciparum
Q94659 Plasmodium falciparum
A0A2P9BSY1 Plasmodium sp. gorilla clade G2
A0A2P9CKT7 Plasmodium sp. DRC-Itaito
A0A2P9GG46 Plasmodium sp. DRC-Itaito
W4ITV4 Plasmodium falciparum
A0A812K2K7 Symbiodinium necroappetens
A0A812IE99 Symbiodinium natans
A0A0C2M9K0 Thelohanellus kitauei
Q3SDJ6 Paramecium tetraurelia
Q3SDM2 Paramecium tetraurelia
Q3SDM6 Paramecium tetraurelia
Q3SD54 Paramecium tetraurelia
D2V1N7 Naegleria gruberi
A0A7S2JSE9 Haptolina brevifila
A0A7S3DFN8 Palpitomonas bilix
A0A4Y2AL00 Araneus ventricosus
A0A6P4YM79 Branchiostoma belcheri
A0A4U8URS0 Steinernema carpocapsae
A0A0K0E4U5 Strongyloides stercoralis
A0A090LNS9 Strongyloides ratti
A0A0N4Z015 Parastrongyloides trichosuri
A0A0K0EV93 Strongyloides venezuelensis
A0A0N5B7E3 Strongyloides papillosus
F1LAQ0 Ascaris suum
E4YFK5 Oikopleura dioica
E4X453 Oikopleura dioica
A0A1I8HBZ8 Macrostomum lignano
A0A267GBT0 Macrostomum lignano
A0A1I8I5X4 Macrostomum lignano
A0A2A6CCI0 Pristionchus pacificus
A0A0N4V9P2 Enterobius vermicularis
A0A816QNG3 Rotaria magnacalcarata
R7TCP3 Capitella teleta
A0A023GIH7 Amblyomma triste
A0A7M5WZ22 Clytia hemisphaerica
A0A8C9SBL6 Scleropages formosus
A0A8D1XRF6 Sus scrofa
A0A8D0G4V8 Strix occidentalis caurina
A0A8C3EUA6 Corvus moneduloides
A0A669PIE6 Phasianus colchicus
A0A3P8WB11 Cynoglossus semilaevis
A0A8B9BYZ8 Anser brachyrhynchus

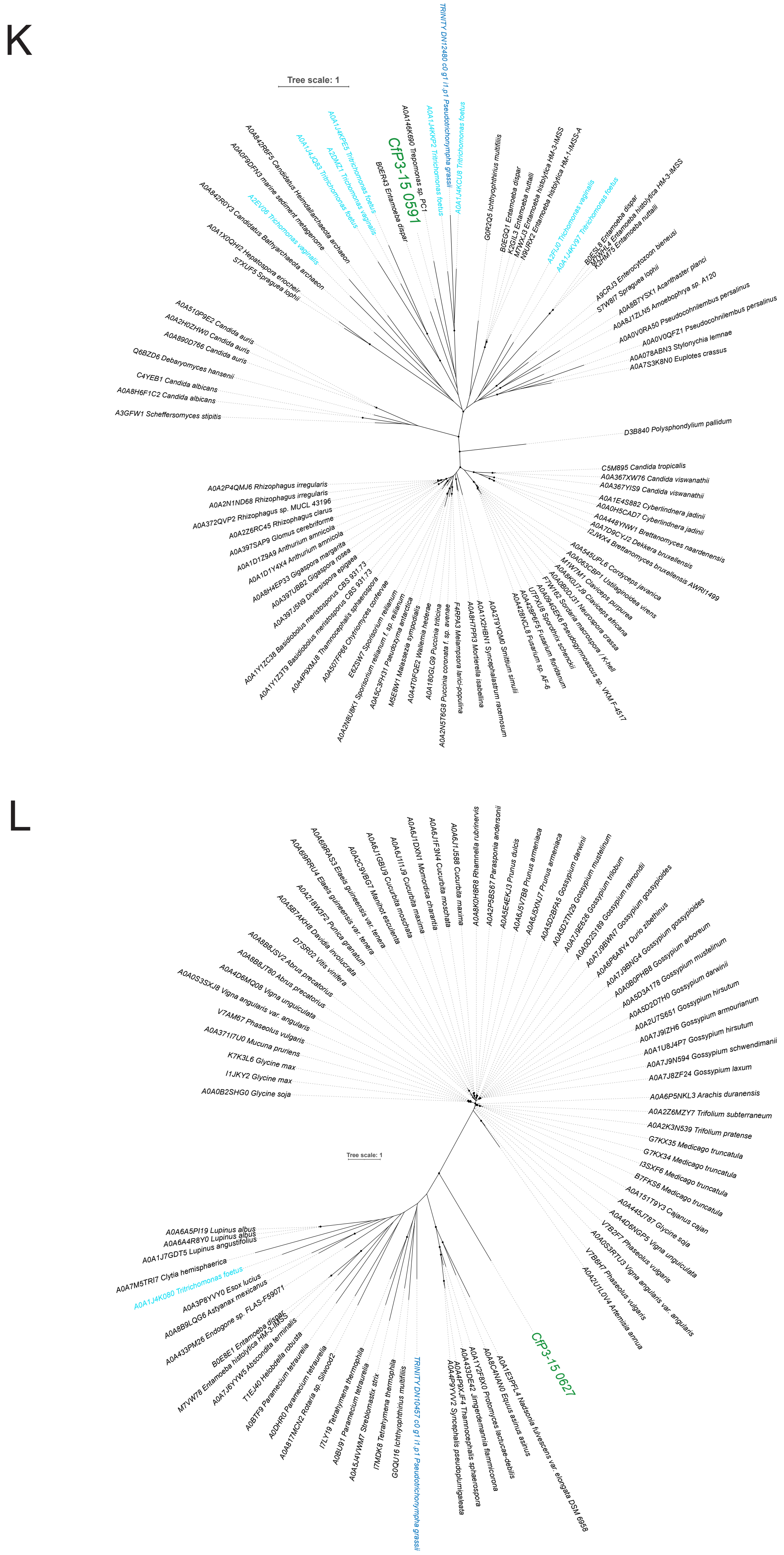
K
Tree scale: 1
TRINITY DN12480 c0 g1 i1 p1 Pseudotrichonympha grassii
A0A1J4JCU8 Tritrichomonas foetus
A0A1J4KKP2 Tritrichomonas foetus
A0A146K690 Trepomonas sp. PC1
CfP3-15 0591
B0ER43 Entamoeba dispar
A0A1J4KPE5 Tritrichomonas foetus
A2DMZ1 Trichomonas vaginalis
A0A1J4JQ83 Tritrichomonas foetus
A0A842R6F5 Candidatus Heimdallarchaeota archaeon
A0A0F9DFN3 marine sediment metagenome
A2EV06 Trichomonas vaginalis
A0A842R0Y3 Candidatus Bathyarchaeota archaeon
A0A1X0QHI2 Hepatospora eriocheir
S7XUF5 Spraguea lophii
G0R2Q5 Ichthyophthirius multifiliis
B0EGQ1 Entamoeba dispar
K2GIL3 Entamoeba nuttalli
M7WXJ3 Entamoeba histolytica HM-3:IMSS
N9URX2 Entamoeba histolytica HM-1:IMSS-A
A2FIJ0 Trichomonas vaginalis
A0A1J4KV97 Tritrichomonas foetus
B0ESL6 Entamoeba dispar
M7WHL6 Entamoeba histolytica HM-3:IMSS
K2MM75 Entamoeba nuttalli
A9CRJ3 Enterocytozoon bieneusi
S7W8I7 Spraguea lophii
A0A8B7YSX1 Acanthaster planci
A0A8J1ZLN5 Amoebophrya sp. A120
A0A0V0RA50 Pseudocohnilembus persalinus
A0A0V0QFZ1 Pseudocohnilembus persalinus
A0A078ABN3 Stylonychia lemnae
A0A7S3K8N0 Euplotes crassus
A0A510P9E2 Candida auris
A0A2H0ZHW0 Candida auris
A0A890D766 Candida auris
Q6BZD6 Debaryomyces hansenii
C4YEB1 Candida albicans
A0A8H6F1C2 Candida albicans
A3GFW1 Scheffersomyces stipitis
D3B840 Polysphondylium pallidum
C5M895 Candida tropicalis
A0A367XW76 Candida viswanathii
A0A367YIS9 Candida viswanathii
A0A1E4S882 Cyberlindnera jadinii
A0A0H5CAD7 Cyberlindnera jadinii
A0A448YNW1 Brettanomyces naardenensis
A0A7D9CYJ2 Dekkera bruxellensis
I2JWX4 Brettanomyces bruxellensis AWRI1499
A0A545UPL6 Cordyceps javanica
A0A063CBP1 Ustilaginoidea virens
M1W7M1 Claviceps purpurea
A0A8K0J1J9 Claviceps africana
A0A8B0DJ31 Neurospora crassa
F7W162 Sordaria macrospora k-hell
A0A094GEK6 Pseudogymnoascus sp. VKM F-4517
U7PXU8 Sporothrix schenckii
A0A428P6F5 Fusarium floridanum
A0A428NCL8 Fusarium sp. AF-6
A0A2P4QMJ6 Rhizophagus irregularis
A0A2N1ND68 Rhizophagus irregularis
A0A372QVP2 Rhizophagus sp. MUCL 43196
A0A2Z6RC45 Rhizophagus clarus
A0A397SAP9 Glomus cerebriforme
A0A1D1Z9A9 Anthurium amnicola
A0A1D1Y4X4 Anthurium amnicola
A0A8H4EP33 Gigaspora margarita
A0A397UBB2 Gigaspora rosea
A0A397J5N9 Diversispora epigaea
A0A1Y1ZC38 Basidiobolus meristosporus CBS 931.73
A0A1Y1Z3T9 Basidiobolus meristosporus CBS 931.73
A0A4P9XMJ8 Thamnocephalis sphaerospora
A0A507FP86 Chytriomyces confervae
E6ZSW7 Sporisorium reilianum
A0A2N8U8K1 Sporisorium reilianum f. sp. reilianum
A0A5C3FH31 Pseudozyma antarctica
M5E8W1 Malassezia sympodialis
A0A4T0FQE2 Wallemia hederae
A0A180GLG9 Puccinia triticina
A0A2N5T6G8 Puccinia coronata f. sp. avenae
F4RPA3 Melampsora larici-populina
A0A8H7PPI3 Mortierella isabellina
A0A1X2HBN1 Syncephalastrum racemosum
A0A2T9YQM0 Smittium simulii
L
Tree scale: 1
A0A6J0H8R8 Rhamnella rubrinervis
A0A2P5BS67 Parasponia andersonii
A0A6J1J588 Cucurbita maxima
A0A6J1F3N4 Cucurbita moschata
A0A6J1DXN1 Momordica charantia
A0A6J1I1J9 Cucurbita maxima
A0A6J1GBU9 Cucurbita moschata
A0A2C9VBG7 Manihot esculenta
A0A6J8RAS3 Elaeis guineensis var. tenera
A0A6I9RRU4 Elaeis guineensis var. tenera
A0A218WGF2 Punica granatum
A0A5B7AKH8 Davidia involucrata
D7SR02 Vitis vinifera
A0A8B8JSV2 Abrus precatorius
A0A8B8JT80 Abrus precatorius
A0A4D6MQ08 Vigna unguiculata
A0A0S3SXJ8 Vigna angularis var. angularis
V7AM67 Phaseolus vulgaris
A0A371I7U0 Mucuna pruriens
K7K3L6 Glycine max
I1JKY2 Glycine max
A0A0B2SHG0 Glycine soja
A0A5E4EKJ3 Prunus dulcis
A0A6J5Y7B8 Prunus armeniaca
A0A6J5XNJ7 Prunus armeniaca
A0A5D2BFA5 Gossypium darwinii
A0A5D2TN29 Gossypium mustelinum
A0A7J9E626 Gossypium trilobum
A0A0D2S189 Gossypium raimondii
A0A7J8BWN7 Gossypium gossypioides
A0A6P6A8Y4 Durio zibethinus
A0A7J9BNG4 Gossypium gossypioides
A0A0B0PHB8 Gossypium arboreum
A0A5D3A178 Gossypium mustelinum
A0A5D2D7H0 Gossypium darwinii
A0A2U7S651 Gossypium hirsutum
A0A7J9IZH6 Gossypium armourianum
A0A1U8J4P7 Gossypium hirsutum
A0A7J9N594 Gossypium schwendimanii
A0A7J8ZF24 Gossypium laxum
A0A6P5NKL3 Arachis duranensis
A0A2Z6MZY7 Trifolium subterraneum
A0A2K3N539 Trifolium pratense
G7KX35 Medicago truncatula
G7KX34 Medicago truncatula
I3SXF6 Medicago truncatula
B7FKS6 Medicago truncatula
A0A151T9Y3 Cajanus cajan
A0A445J787 Glycine soja
A0A4D6NGP5 Vigna unguiculata
V7B2F7 Phaseolus vulgaris
A0A0S3RTU3 Vigna angularis var. angularis
V7B6H7 Phaseolus vulgaris
A0A2U1L0V4 Artemisia annua
A0A6A5PI19 Lupinus albus
A0A6A4R8Y0 Lupinus albus
A0A1J7GDT5 Lupinus angustifolius
A0A7M5TRI7 Clytia hemisphaerica
A0A1J4K080 Tritrichomonas foetus
A0A3P8YVY0 Esox lucius
A0A8B9LQG6 Astyanax mexicanus
A0A433PM26 Endogone sp. FLAS-F59071
B0E8E1 Entamoeba dispar
M7VW78 Entamoeba histolytica HM-3:IMSS
A0A7J6YYW5 Abscondita terminalis
T1EJ40 Helobdella robusta
A0BTF9 Paramecium tetraurelia
A0DHR0 Paramecium tetraurelia
A0A817MCN2 Rotaria sp. Silwood2
I7LY19 Tetrahymena thermophila
A0BU91 Paramecium tetraurelia
A0A5J4VWM7 Streblomastix strix
I7MDK8 Tetrahymena thermophila
G0QU16 Ichthyophthirius multifiliis
TRINITY DN10457 c0 g1 i1 p1 Pseudotrichonympha grassii
A0A4P9YVV2 Syncephalis pseudoplumigaleata
A0A4P9XJF4 Thamnocephalis sphaerospora
A0A433DE42 Jimgerdemannia flammicorona
A0A1Y2F6X0 Protomyces lactucae-debilis
A0A8C4NAN0 Equus asinus asinus
A0A1E3PFL4 Nadsonia fulvescens var. elongata DSM 6958
CfP3-15 0627

M

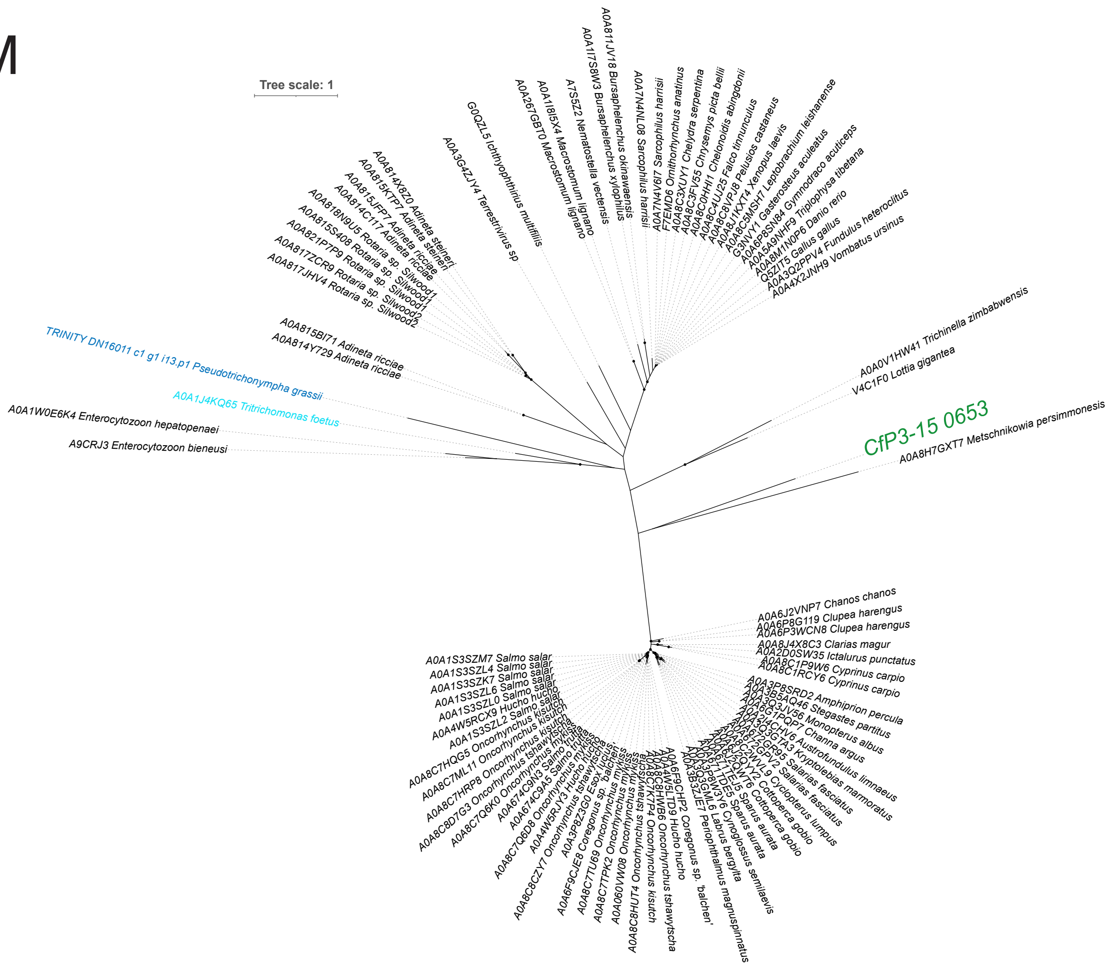
Tree scale: 1
CfP3-15 0653
A0A8H7GXT7 Metschnikowia persimmonesis
A0A0V1HW41 Trichinella zimbabwensis
V4C1F0 Lottia gigantea
TRINITY DN16011 c1 g1 i13.p1 Pseudotrichonympha grassii
A0A1J4KQ65 Tritrichomonas foetus
A0A1W0E6K4 Enterocytozoon hepatopenaei
A9CRJ3 Enterocytozoon bieneusi
A0A815BI71 Adineta ricciae
A0A814Y729 Adineta ricciae
G0QZL5 Ichthyophthirius multifiliis
A0A3G4ZJY4 Terrestrivirus sp
A7S5Z2 Nematostella vectensis
A0A6J2VNP7 Chanos chanos
A0A8J4X8C3 Clarias magur

N

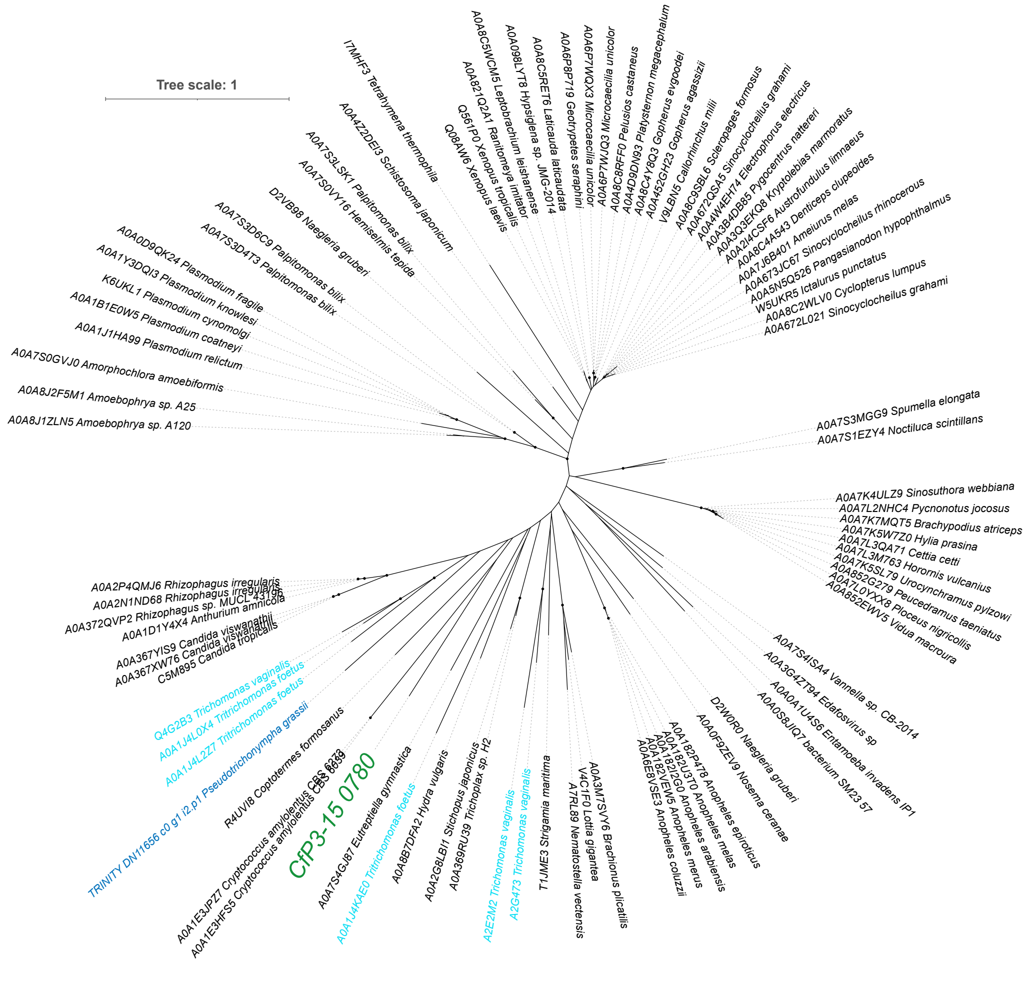
Tree scale: 1
CfP3-15 0780
I7MHF3 Tetrahymena thermophila
A0A4Z2DEI3 Schistosoma japonicum
D2VB98 Naegleria gruberi
A0A7S3MGG9 Spumella elongata
A0A7S1EZY4 Noctiluca scintillans
A0A7K4ULZ9 Sinosuthora webbiana
A0A7L2NHC4 Pycnonotus jocosus
A0A7S0GVJ0 Amorphochlora amoebiformis
A0A8J2F5M1 Amoebophrya sp. A25
A0A8J1ZLN5 Amoebophrya sp. A120
A0A7S4ISA4 Vannella sp. CB-2014
A0A3G4ZT94 Edafosvirus sp
A0A0A1U4S6 Entamoeba invadens IP1
A0A0S8JIQ7 bacterium SM23 57
D2W0R0 Naegleria gruberi
A0A0F9ZEV9 Nosema ceranae
A0A3M7SVY6 Brachionus plicatilis
V4C1F0 Lottia gigantea
A7RL89 Nematostella vectensis
T1JME3 Strigamia maritima
A2E2M2 Trichomonas vaginalis
A2G473 Trichomonas vaginalis
A0A1J4KAE0 Tritrichomonas foetus
A0A8B7DFA2 Hydra vulgaris
R4UVI8 Coptotermes formosanus
TRINITY DN11656 c0 g1 i2.p1 Pseudotrichonympha grassii
Q4G2B3 Trichomonas vaginalis
A0A1J4L0X4 Tritrichomonas foetus
A0A1J4L2Z7 Tritrichomonas foetus
C5M895 Candida tropicalis
A0A2P4QMJ6 Rhizophagus irregularis

O

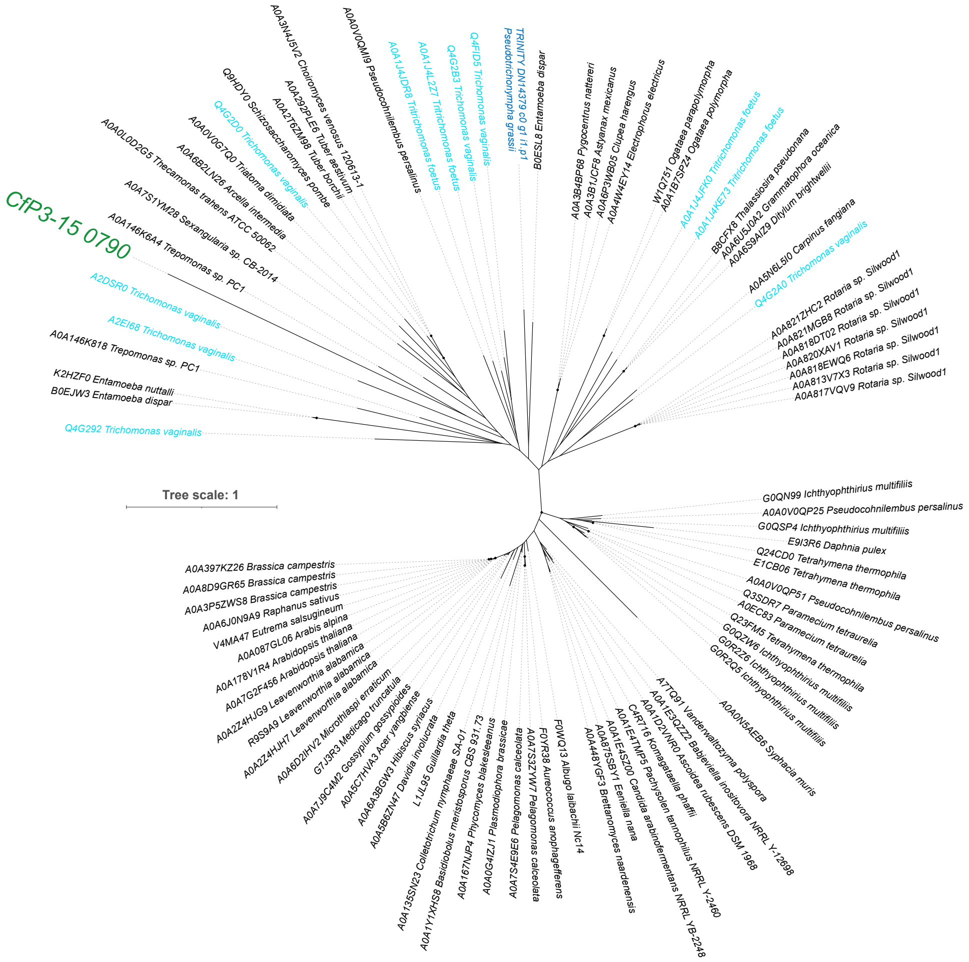
CfP3-15 0790
A2DSR0 Trichomonas vaginalis
A2EI66 Trichomonas vaginalis
A0A146K818 Trepomonas sp. PC1
K2HZF0 Entamoeba nuttalli
B0EJW3 Entamoeba dispar
Q4G292 Trichomonas vaginalis
A0A146K6A4 Trepomonas sp. PC1
A0A7S1YM28 Sexangularia sp. CB-2014
A0A0L0D2G5 Thecamonas trahens ATCC 50062
A0A6B2LN26 Arcella intermedia
A0A0V0G7Q0 Triatoma dimidiata
Q4G2D0 Trichomonas vaginalis
Q9HDV0 Schizosaccharomyces pombe
A0A2T6ZM98 Tuber borchii
A0A292PLE6 Tuber aestivum
A0A3N4J5V2 Choiromyces venosus 120613-1
A0A0V0QMI9 Pseudocohnilembus persalinus
A0A1J4JDR8 Tritrichomonas foetus
A0A1J4L2Z7 Tritrichomonas foetus
Q4G2B3 Trichomonas vaginalis
Q4FID5 Trichomonas vaginalis
TRINITY DN14379 c0 g1 i1 p1
Pseudotrichonympha grassii
B0ESL8 Entamoeba dispar
A0A3B4BF68 Pygocentrus nattereri
A0A3B1JCF8 Astyanax mexicanus
A0A6P3WB05 Clupea harengus
A0A4W4EY14 Electrophorus electricus
W1QI51 Ogataea parapolymorpha
A0A1B7SPZ4 Ogataea polymorpha
A0A1J4JFK0 Tritrichomonas foetus
A0A1J4KE73 Tritrichomonas foetus
B8CFX8 Thalassiosira pseudonana
A0A6U5J0A3 Grammatophora oceanica
A0A6S9AI29 Ditylum brightwellii
A0A5N6L5I0 Carpinus fangiana
Q4G2A0 Trichomonas vaginalis
A0A821ZHC2 Rotaria sp. Silwood1
A0A821MGB8 Rotaria sp. Silwood1
A0A818DT02 Rotaria sp. Silwood1
A0A820XAV1 Rotaria sp. Silwood1
A0A818EWQ6 Rotaria sp. Silwood1
A0A813V7X3 Rotaria sp. Silwood1
A0A817VQV9 Rotaria sp. Silwood1
Tree scale: 1
G0QN99 Ichthyophthirius multifiliis
A0A0V0QP25 Pseudocohnilembus persalinus
G0QSP4 Ichthyophthirius multifiliis
E9I3R6 Daphnia pulex
Q24CD0 Tetrahymena thermophila
E1CB06 Tetrahymena thermophila
A0A0V0QP51 Pseudocohnilembus persalinus
Q3SDR7 Paramecium tetraurelia
A0EC83 Paramecium tetraurelia
Q23FM5 Tetrahymena thermophila
G0QZW6 Ichthyophthirius multifiliis
G0R226 Ichthyophthirius multifiliis
G0R2Q5 Ichthyophthirius multifiliis
A0A0N5AEB6 Syphacia muris
A7TQ91 Vanderwaltozyma polyspora
A0A1E3QZZ2 Babjeviella inositovora NRRL Y-12698
A0A1D2VNR0 Ascoidea rubescens DSM 1968
C4R716 Komagataella phaffii
A0A1E4TMP5 Pachysolen tannophilus NRRL Y-2460
A0A1E4SZ00 Candida arabinofermentans NRRL YB-2248
A0A6T5SBY1 Eenlella nana
A0A448YGF3 Brettanomyces naardenensis
F0WQ13 Albugo laibachii Nc14
F0YR38 Aureococcus anophagefferens
A0A7S3ZYW7 Pelagomonas calceolata
A0A7S4E9E6 Pelagomonas calceolata
A0A0G4IZJ1 Plasmodiophora brassicae
A0A167NJP4 Phycomyces blakesleeanus
A0A1Y1XHS8 Basidiobolus meristosporus CBS 931.73
A0A135SN23 Colletotrichum nymphaeae SA-01
L1JL95 Guillardia theta
A0A5B6ZN47 Davidia involucrata
A0A6A3BGW3 Hibiscus syriacus
A0A5C7HVA3 Acer yangbiense
A0A7J9C4M2 Gossypium gossypioides
G7J3R3 Medicago truncatula
A0A6D2IHV2 Microthlaspi erraticum
A0A2Z4HJH7 Leavenworthia alabamica
R9S9A9 Leavenworthia alabamica
A0A2Z4HJG9 Leavenworthia alabamica
A0A7G2F456 Arabidopsis thaliana
A0A178V1R4 Arabidopsis thaliana
A0A087GL06 Arabis alpina
V4MA47 Eutrema salsugineum
A0A6J0N9A9 Raphanus sativus
A0A3P5ZWS8 Brassica campestris
A0A8D9GR65 Brassica campestris
A0A397KZ26 Brassica campestris

P

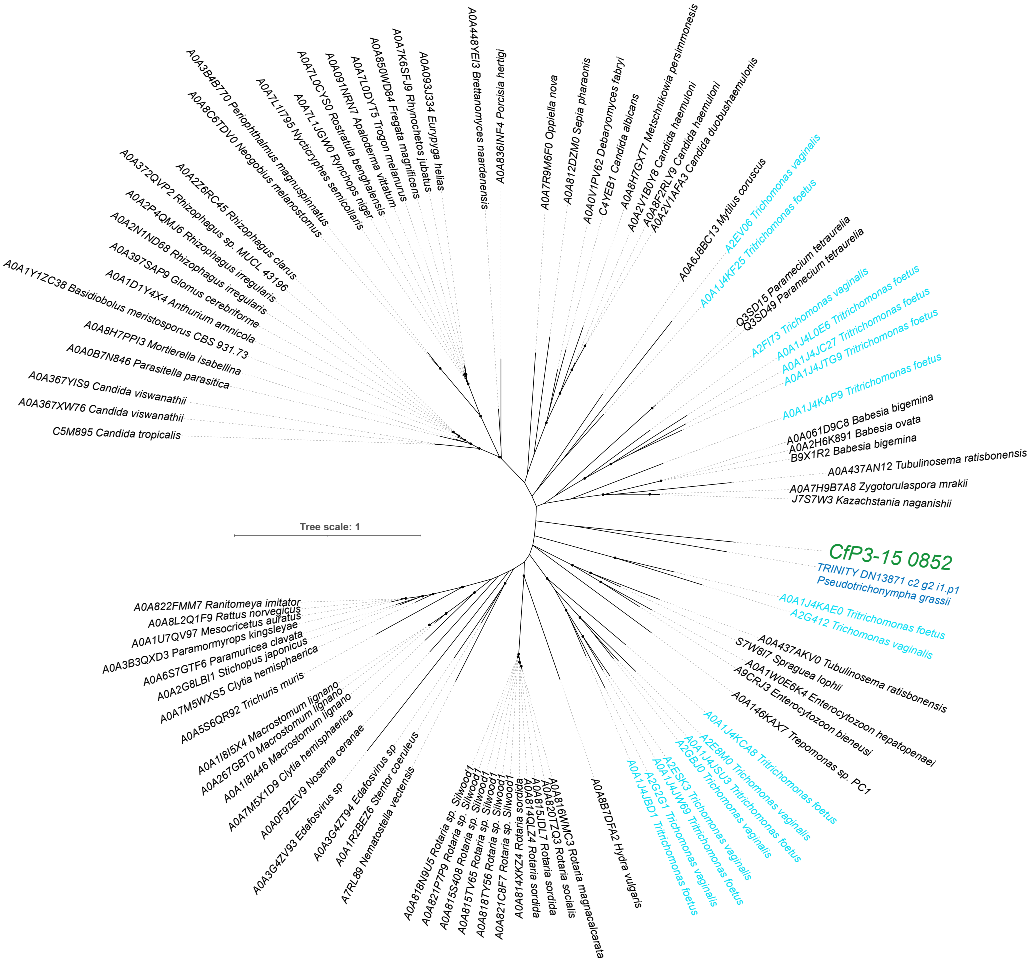
A0A3B4B770 Periophthalmus magnuspinnatus
A0A8C6TDV0 Neogobius melanostomus
A0A7L1I795 Nyctibius grandis
A0A7L1JGW0 Rynchops niger
A0A7L0CYS0 Rostratula benghalensis
A0A091NRN7 Apaloderma vittatum
A0A7L0DYT5 Trogon melanurus
A0A8S0WD84 Fregata magnificens
A0A7K6SFJ9 Rhynochetos jubatus
A0A093J334 Eurypyga helias
A0A448YEI3 Brettanomyces naardenensis
A0A836INF4 Porcisia hertigi
A0A7R9M6F0 Oppiella nova
A0A812DZM0 Sepia pharaonis
A0A0V1PV62 Debaryomyces fabryi
C4YEB1 Candida albicans
A0A8H7GXT7 Metschnikowia persimmonesis
A0A2V1B0Y8 Candida haemuloni
A0A8F2RLY9 Candida haemuloni
A0A2V1AFA3 Candida duobushaemulonis
A0A6J8BC13 Mytilus coruscus
A2EV06 Trichomonas vaginalis
A0A1J4KF25 Tritrichomonas foetus
Q3SD15 Paramecium tetraurelia
Q3SD49 Paramecium tetraurelia
A2FI73 Trichomonas vaginalis
A0A1J4L0E6 Tritrichomonas foetus
A0A1J4JC27 Tritrichomonas foetus
A0A1J4JTG9 Tritrichomonas foetus
A0A1J4KAP9 Tritrichomonas foetus
A0A061D9C8 Babesia bigemina
A0A2H6K891 Babesia ovata
B9X1R2 Babesia bigemina
A0A437AN12 Tubulinosema ratisbonensis
A0A7H9B7A8 Zygotorulaspora mrakii
J7S7W3 Kazachstania naganishii
A0A372QVP2 Rhizophagus sp. MUCL 43196
A0A2Z6RC45 Rhizophagus clarus
A0A2P4QMJ6 Rhizophagus irregularis
A0A2N1ND68 Rhizophagus irregularis
A0A397SAP9 Glomus cerebriforme
A0A1D1Y4X4 Anthurium amnicola
A0A1Y1ZC38 Basidiobolus meristosporus CBS 931.73
A0A8H7PPI3 Mortierella isabellina
A0A0B7N846 Parasitella parasitica
A0A367YIS9 Candida viswanathii
A0A367XW76 Candida viswanathii
C5M895 Candida tropicalis
Tree scale: 1
CfP3-15 0852
TRINITY DN13871 c2 g2 i1 p1
Pseudotrichonympha grassii
A0A1J4KAE0 Tritrichomonas foetus
A2G412 Trichomonas vaginalis
A0A437AKV0 Tubulinosema ratisbonensis
S7W8I7 Spraguea lophii
A0A1W0E6K4 Enterocytozoon hepatopenaei
A9CRJ3 Enterocytozoon bieneusi
A0A146KAX7 Trepomonas sp. PC1
A0A1J4KCA8 Tritrichomonas foetus
A2E8M0 Trichomonas vaginalis
A0A1J4JSJ3 Tritrichomonas foetus
A2GBJ0 Trichomonas vaginalis
A2ESK3 Trichomonas vaginalis
A0A1J4JW69 Tritrichomonas foetus
A2G2G1 Trichomonas vaginalis
A0A1J4JBD1 Tritrichomonas foetus
A0A8B7DFA2 Hydra vulgaris
A0A816WNC3 Rotaria magnacalcarata
A0A820TZQ3 Rotaria socialis
A0A815UDU7 Rotaria sordida
A0A814QLZ4 Rotaria sordida
A0A814XKZ4 Rotaria sordida
A0A821C8F7 Rotaria sp. Silwood1
A0A818TY56 Rotaria sp. Silwood1
A0A815TV65 Rotaria sp. Silwood1
A0A815S408 Rotaria sp. Silwood1
A0A821P7P9 Rotaria sp. Silwood1
A0A818N9U5 Rotaria sp. Silwood1
A7RL89 Nematostella vectensis
A0A1R2BEZ6 Stentor coeruleus
A0A3G4ZT94 Edafosvirus sp
A0A3G4ZV93 Edafosvirus sp
A0A0F9ZEV9 Nosema ceranae
A0A7M5X1D9 Clytia hemisphaerica
A0A1I8I446 Macrostomum lignano
A0A267GBT0 Macrostomum lignano
A0A1I8I5X4 Macrostomum lignano
A0A5S6QR92 Trichuris muris
A0A7M5WXS5 Clytia hemisphaerica
A0A2G8LBI1 Stichopus japonicus
A0A6S7GTF6 Paramuricea clavata
A0A3B3QXD3 Paramormyrops kingsleyae
A0A1U7QV97 Mesocricetus auratus
A0A8L2Q1F9 Rattus norvegicus
A0A822FMM7 Ranitomeya imitator

Q

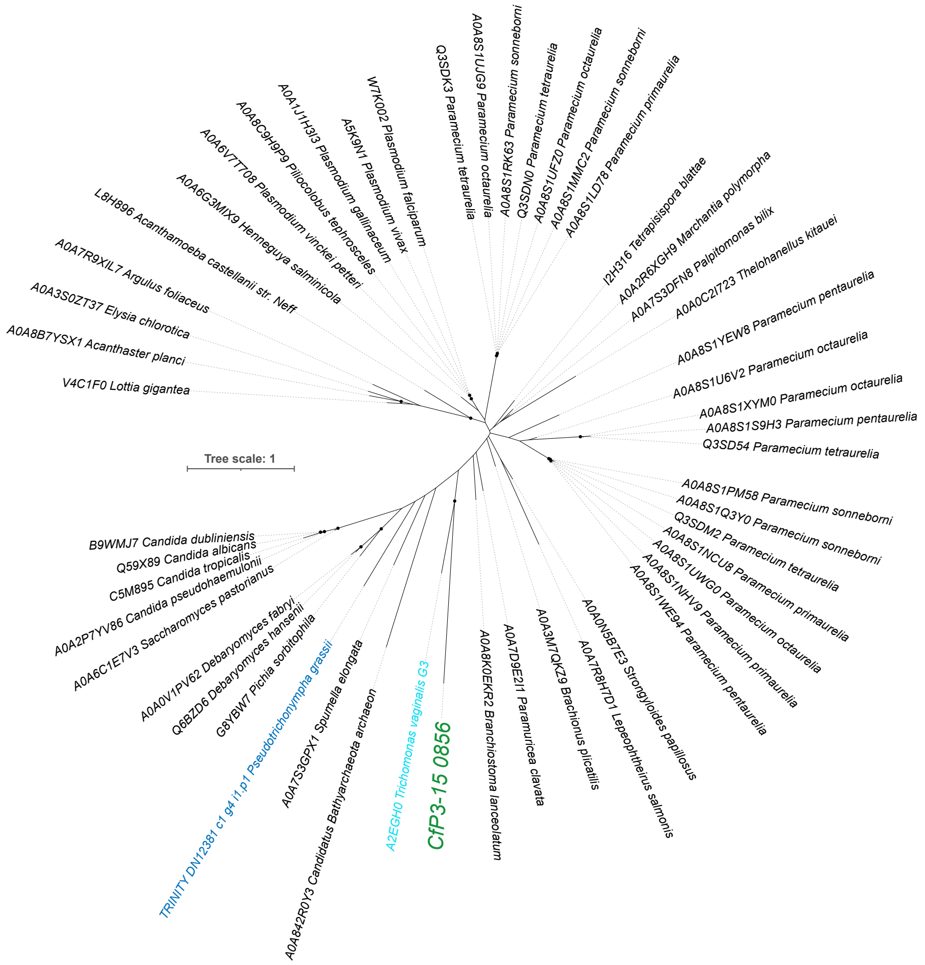
Tree scale: 1
Q3SDK3 Paramecium tetraurelia
A0A8S1UJG9 Paramecium octaurelia
A0A8S1RK63 Paramecium sonneborni
Q3SDN0 Paramecium tetraurelia
A0A8S1UF70 Paramecium octaurelia
A0A8S1MMC2 Paramecium sonneborni
A0A8S1LD78 Paramecium primaurelia
I2H316 Tetrapisispora blattae
A0A2R6XGH9 Marchantia polymorpha
A0A7S3DFN8 Palpitomonas bilix
A0A0C2I723 Thelohanellus kitauei
A0A8S1YEW8 Paramecium pentaurelia
A0A8S1U6V2 Paramecium octaurelia
A0A8S1XYM0 Paramecium octaurelia
A0A8S1S9H3 Paramecium pentaurelia
Q3SD54 Paramecium tetraurelia
A0A8S1PM58 Paramecium sonneborni
A0A8S1Q3Y0 Paramecium sonneborni
Q3SDM2 Paramecium tetraurelia
A0A8S1NCU8 Paramecium primaurelia
A0A8S1UWG0 Paramecium octaurelia
A0A8S1NHV9 Paramecium primaurelia
A0A8S1WE94 Paramecium pentaurelia
A0A0N5B7E3 Strongyloides papillosus
A0A7R8H7D1 Lepeophtheirus salmonis
A0A3M7QKZ9 Brachionus plicatilis
A0A7D9E2I1 Paramuricea clavata
A0A8K0EKR2 Branchiostoma lanceolatum
CfP3-15 0856
A2EGH0 Trichomonas vaginalis G3
A0A842R0Y3 Candidatus Bathyarchaeota archaeon
A0A7S3GPX1 Spumella elongata
TRINITY DN12381 c1 g4 i1.p1 Pseudotrichonympha grassii
G8YBW7 Pichia sorbitophila
Q6BZD6 Debaryomyces hansenii
A0A0V1PV62 Debaryomyces fabryi
A0A6C1E7V3 Saccharomyces pastorianus
A0A2P7YV86 Candida pseudohaemulonii
C5M895 Candida tropicalis
Q59X89 Candida albicans
B9WMJ7 Candida dubliniensis
V4C1F0 Lottia gigantea
A0A8B7YSX1 Acanthaster planci
A0A3S0ZT37 Elysia chlorotica
A0A7R9XIL7 Argulus foliaceus
L8H696 Acanthamoeba castellanii str. Neff
A0A6G3MIX9 Henneguya salminicola
A0A6V7T708 Plasmodium vinckei petteri
A0A8C9H9P9 Piliocolobus tephrosceles
A0A1J1H3I3 Plasmodium gallinaceum
A5K9N1 Plasmodium vivax
W7K002 Plasmodium falciparum

R

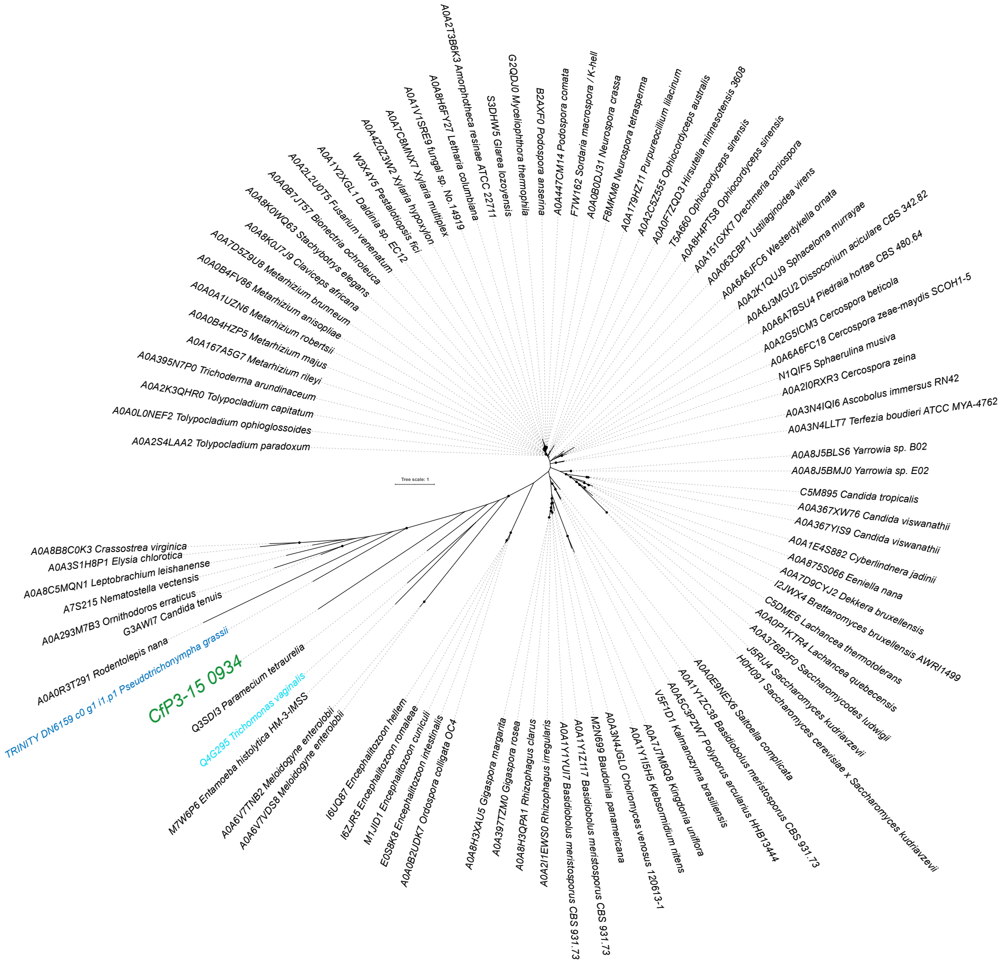
Tree scale: 1
A0A2T3B6K3 Amorphotheca resinae ATCC 22711
S3DHW5 Glarea lozoyensis
G2QDJ0 Myceliophthora thermophila
B2AXF0 Podospora anserina
A0A447CM14 Podospora comata
F7W162 Sordaria macrospora / K-hell
A0A0B0DJ31 Neurospora crassa
F8MKM8 Neurospora tetrasperma
A0A179HZ11 Purpureocillium lilacinum
A0A2C5Z555 Ophiocordyceps australis
A0A0F7ZQD3 Hirsutella minnesotensis 3608
T5A660 Ophiocordyceps sinensis
A0A8H4PTS8 Ophiocordyceps sinensis
A0A151GXK7 Drechmeria coniospora
A0A063CBP1 Ustilaginoidea virens
A0A6A6JFC6 Westerdykella ornata
A0A2K1QUJ9 Sphaceloma murrayae
A0A6J3MGU2 Dissoconium aciculare CBS 342.82
A0A6A7BSU4 Piedraia hortae CBS 480.64
A0A2G5ICM3 Cercospora beticola
A0A6A6FC18 Cercospora zeae-maydis SCOH1-5
N1QIF5 Sphaerulina musiva
A0A2I0RXR3 Cercospora zeina
A0A3N4IQI6 Ascobolus immersus RN42
A0A3N4LLT7 Terfezia boudieri ATCC MYA-4762
A0A8J5BLS6 Yarrowia sp. B02
A0A8J5BMJ0 Yarrowia sp. E02
C5M895 Candida tropicalis
A0A367XW76 Candida viswanathii
A0A367YIS9 Candida viswanathii
A0A1E4S882 Cyberlindnera jadinii
A0A875S066 Eeniella nana
A0A7D9CYJ2 Dekkera bruxellensis
I2JWX4 Brettanomyces bruxellensis AWRI1499
C5DME6 Lachancea thermotolerans
A0A0P1KTR4 Lachancea quebecensis
A0A376B2F0 Saccharomycodes ludwigii
J5RIJ4 Saccharomyces kudriavzevii
H0H091 Saccharomyces cerevisiae x Saccharomyces kudriavzevii
A0A0E9NEX6 Saitoella complicata
A0A1Y1ZC38 Basidiobolus meristosporus CBS 931.73
A0A5C3PZW7 Polyporus arcularius HHB13444
V5F1D1 Kalmanozyma brasiliensis
A0A7J7M8Q8 Kingdonia uniflora
A0A1Y1I5H5 Klebsormidium nitens
A0A3N4JGL0 Choiromyces venosus 120613-1
M2N699 Baudoinia panamericana
A0A1Y1Z117 Basidiobolus meristosporus CBS 931.73
A0A1Y1YUI7 Basidiobolus meristosporus CBS 931.73
A0A2I1EWS0 Rhizophagus irregularis
A0A8H3QPA1 Rhizophagus clarus
A0A397TZM0 Gigaspora rosea
A0A8H3XAU5 Gigaspora margarita
A0A0B2UDK7 Ordospora colligata OC4
E0S8K8 Encephalitozoon intestinalis
M1JID1 Encephalitozoon cuniculi
I6ZJR5 Encephalitozoon romaleae
I6UQ87 Encephalitozoon hellem
A0A6V7VDS8 Meloidogyne enterolobii
A0A6V7TNB2 Meloidogyne enterolobii
M7W6P6 Entamoeba histolytica HM-3-IMSS
Q4G295 Trichomonas vaginalis
Q3SDI3 Paramecium tetraurelia
CfP3-15 0934
TRINITY DN6159 c0 g1 i1.p1 Pseudotrichonympha grassii
A0A0R3T291 Rodentolepis nana
G3AWI7 Candida tenuis
A0A293M7B3 Ornithodoros erraticus
A7S215 Nematostella vectensis
A0A8C5MQN1 Leptobrachium leishanense
A0A3S1H8P1 Elysia chlorotica
A0A8B8C0K3 Crassostrea virginica
A0A2S4LAA2 Tolypocladium paradoxum
A0A0L0NEF2 Tolypocladium ophioglossoides
A0A2K3QHR0 Tolypocladium capitatum
A0A395N7P0 Trichoderma arundinaceum
A0A167A5G7 Metarhizium rileyi
A0A0B4HZP5 Metarhizium majus
A0A0A1UZN6 Metarhizium robertsii
A0A0B4FV86 Metarhizium anisopliae
A0A7D5Z9U8 Metarhizium brunneum
A0A8K0J7J9 Claviceps africana
A0A8K0WQ63 Stachybotrys elegans
A0A0B7JT57 Bionectria ochroleuca
A0A2L2U0T5 Fusarium venenatum
A0A1Y2XGL1 Daldinia sp. EC12
W3X4Y5 Pestalotiopsis fici
A0A4Z0Z3W2 Xylaria hypoxylon
A0A7C8MNX7 Xylaria multiplex
A0A1V1SRE9 fungal sp. No.14919
A0A8H6FY27 Letharia columbiana

S

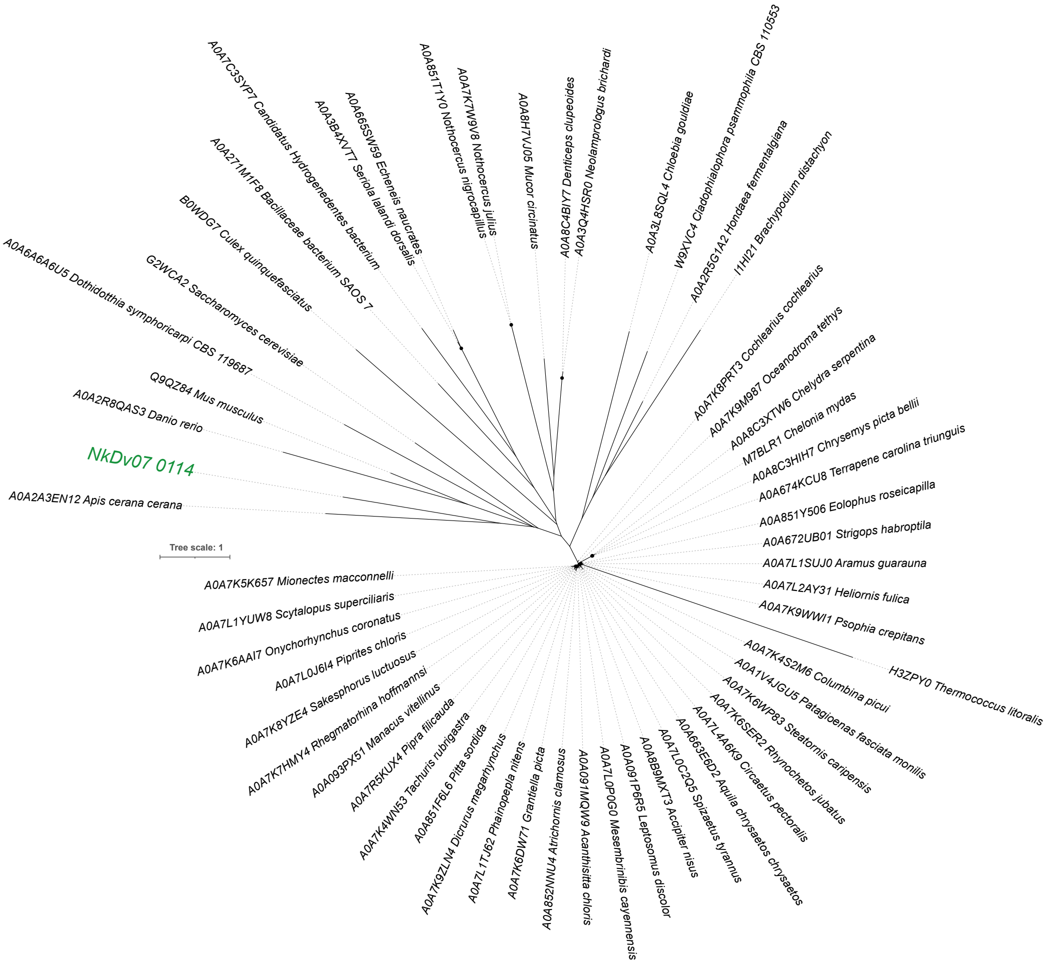
A0A7C3SYP7 Candidatus Hydrogenedentes bacterium
A0A665SW59 Echeneis naucrates
A0A3B4XVT7 Seriola lalandi dorsalis
A0A851T1Y0 Nothocercus nigrocapillus
A0A7K7W9V8 Nothocercus julius
A0A8H7VJ05 Mucor circinatus
A0A8C4BIY7 Denticeps clupeoides
A0A3Q4HSR0 Neolamprologus brichardi
A0A3L8SQL4 Chloebia gouldiae
W9XVC4 Cladophialophora psammophila CBS 110553
A0A2R5G1A2 Hondaea fermentalgiana
I1HI21 Brachypodium distachyon
A0A271M1F8 Bacillaceae bacterium SAOS 7
B0WDG7 Culex quinquefasciatus
G2WCA2 Saccharomyces cerevisiae
A0A6A6A6U5 Dothidotthia symphoricarpi CBS 119687
Q9QZ84 Mus musculus
A0A2R8QAS3 Danio rerio
NkDv07 0114
A0A2A3EN12 Apis cerana cerana
Tree scale: 1
A0A7K8PRT3 Cochlearius cochlearius
A0A7K9M987 Oceanodroma tethys
A0A8C3XTW6 Chelydra serpentina
M7BLR1 Chelonia mydas
A0A8C3HIH7 Chrysemys picta bellii
A0A674KCU8 Terrapene carolina triunguis
A0A851Y506 Eolophus roseicapilla
A0A672UB01 Strigops habroptila
A0A7L1SUJ0 Aramus guarauna
A0A7L2AY31 Heliornis fulica
A0A7K9WWI1 Psophia crepitans
H3ZPY0 Thermococcus litoralis
A0A7K5K657 Mionectes macconnelli
A0A7L1YUW8 Scytalopus superciliaris
A0A7K6AAI7 Onychorhynchus coronatus
A0A7L0J6I4 Piprites chloris
A0A7K6YZE4 Sakesphorus luctuosus
A0A7K7HMY4 Rhegmatorhina hoffmannsi
A0A093PX51 Manacus vitellinus
A0A7R5KUX4 Pipra filicauda
A0A7K4WN53 Tachuris rubrigastra
A0A851F6L6 Pitta sordida
A0A7K9ZLN4 Dicrurus megarhynchus
A0A7L1TJ62 Phainopepla nitens
A0A7K6DW71 Grantiella picta
A0A852NNU4 Atrichornis clamosus
A0A091MQW9 Acanthisitta chloris
A0A7L0P0G0 Mesembrinibis cayennensis
A0A091P6R5 Leptosomus discolor
A0A8B9MXT3 Accipiter nisus
A0A7L0C2Q5 Spizaetus tyrannus
A0A663E6D2 Aquila chrysaetos chrysaetos
A0A7L4A6K9 Circaetus pectoralis
A0A7K6SER2 Rhynochetos jubatus
A0A7K6WP83 Steatornis caripensis
A0A1V4JGU5 Patagioenas fasciata monilis
A0A7K4S2M6 Columbina picui

T

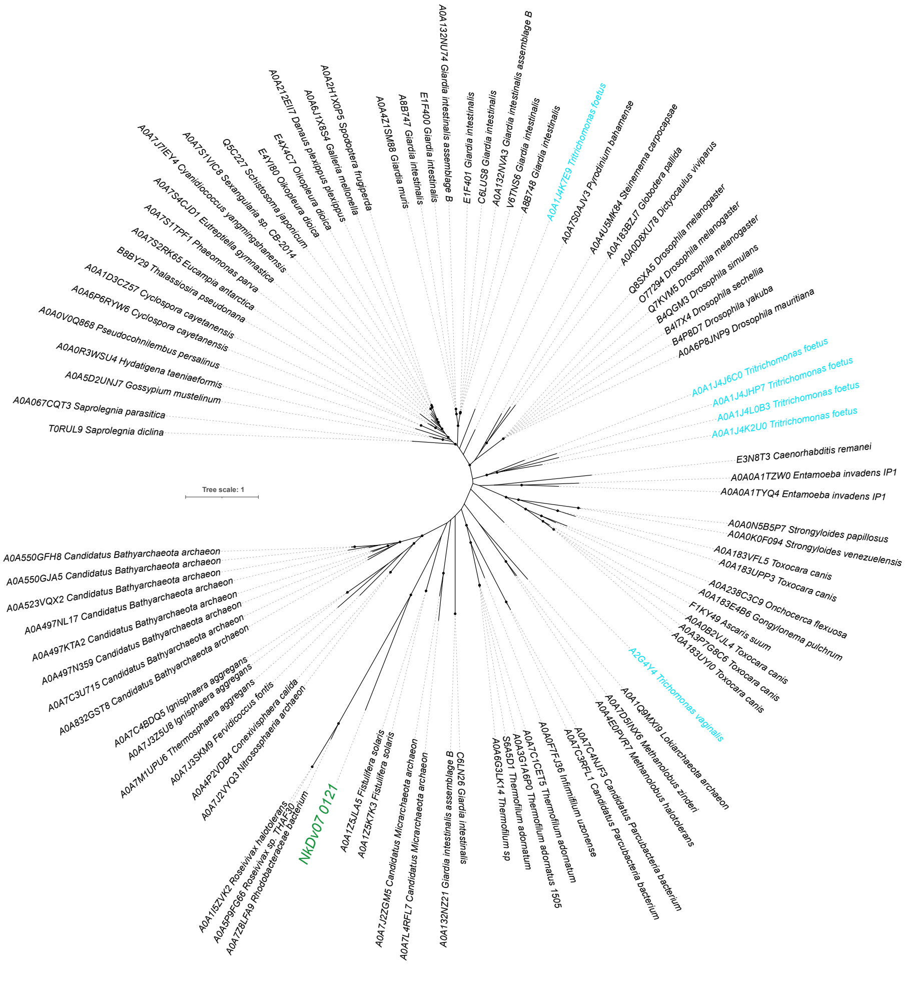
A0A132NU74 Giardia intestinalis assemblage B
A0A2H1X0P5 Spodoptera frugiperda
A0A6J1X8S4 Galleria mellonella
A0A212EII7 Danaus plexippus plexippus
E4XXC7 Oikopleura dioica
E4Y180 Oikopleura dioica
Q5C227 Schistosoma japonicum
A0A7S1VIC8 Sexangularia sp. CB-2014
A0A7J7IEY4 Cyanidiococcus yangmingshanensis
A0A7S4CJD1 Eutreptiella gymnastica
A0A7S1TPF1 Phaeomonas parva
A0A7S2RK65 Eucampia antarctica
B8BY29 Thalassiosira pseudonana
A0A1D3CZ57 Cyclospora cayetanensis
A0A6P6RYW6 Cyclospora cayetanensis
A0A0V0Q868 Pseudocohnilembus persalinus
A0A0R3WSU4 Hydatigena taeniaeformis
A0A5D2UNJ7 Gossypium mustelinum
A0A067CQT3 Saprolegnia parasitica
T0RUL9 Saprolegnia diclina
A0A4Z1SM88 Giardia muris
A8B747 Giardia intestinalis
E1F400 Giardia intestinalis assemblage B
E1F401 Giardia intestinalis
C6LUS8 Giardia intestinalis
A0A132NVA3 Giardia intestinalis assemblage B
V6TNS6 Giardia intestinalis
A8B748 Giardia intestinalis
A0A1J4KYE9 Tritrichomonas foetus
A0A7S0AJV3 Pyrodinium bahamense
A0A4U5MK84 Steinernema carpocapsae
A0A183BZJ7 Globodera pallida
A0A0D8XU78 Dictyocaulus viviparus
Q8SXA5 Drosophila melanogaster
O77294 Drosophila melanogaster
Q7KVM5 Drosophila melanogaster
B4QGM3 Drosophila simulans
B4I7X4 Drosophila sechellia
B4P8D7 Drosophila yakuba
A0A6P8JNP9 Drosophila mauritiana
A0A1J4J8C0 Tritrichomonas foetus
A0A1J4JHP7 Tritrichomonas foetus
A0A1J4L0B3 Tritrichomonas foetus
A0A1J4K2U0 Tritrichomonas foetus
E3N8T3 Caenorhabditis remanei
A0A0A1TZW0 Entamoeba invadens IP1
A0A0A1TYQ4 Entamoeba invadens IP1
Tree scale: 1
A0A0N5B5P7 Strongyloides papillosus
A0A0K0F094 Strongyloides venezuelensis
A0A183VFL5 Toxocara canis
A0A183UPP3 Toxocara canis
A0A238C3C9 Onchocerca flexuosa
A0A183E4B6 Gongylonema pulchrum
F1KY49 Ascaris suum
A0A0B2VJL4 Toxocara canis
A0A3P7G8C6 Toxocara canis
A0A183UYI0 Toxocara canis
A2G4Y4 Trichomonas vaginalis
A0A550GFH8 Candidatus Bathyarchaeota archaeon
A0A550GJA5 Candidatus Bathyarchaeota archaeon
A0A523VQX2 Candidatus Bathyarchaeota archaeon
A0A497NL17 Candidatus Bathyarchaeota archaeon
A0A497KTA2 Candidatus Bathyarchaeota archaeon
A0A497N359 Candidatus Bathyarchaeota archaeon
A0A7C3U715 Candidatus Bathyarchaeota archaeon
A0A832GST8 Candidatus Bathyarchaeota archaeon
A0A7C4BDQ5 Ignisphaera aggregans
A0A7J3Z5U8 Ignisphaera aggregans
A0A7M1UPU6 Thermosphaera aggregans
A0A7J3SKM9 Fervidicoccus fontis
A0A4P2VDB4 Conexivisphaera calida
A0A7J2VYQ3 Nitrososphaera archaeon
A0A1I5ZJK2 Roseivivax halodurans
A0A5P9FG66 Roseivivax sp. THAF30
A0A7Z8LFA9 Rhodobacteraceae bacterium
NkDv07 0121
A0A1Z5JLA5 Fistulifera solaris
A0A1Z5K7K3 Fistulifera solaris
A0A7J2ZGM5 Candidatus Micrarchaeota archaeon
A0A7L4RFL7 Candidatus Micrarchaeota archaeon
A0A132NZ21 Giardia intestinalis assemblage B
C6LN26 Giardia intestinalis
A0A6G3LK14 Thermofilum sp
S6A5D1 Thermofilum adornatum
A0A3G1A6P0 Thermofilum adornatum
A0A7C1CET5 Thermofilum adornatum 1505
A0A0F7FJ36 Infirmifilum uzonense
A0A7C3RFL1 Candidatus Parcubacteria bacterium
A0A7C4NJF3 Candidatus Parcubacteria bacterium
A0A4E0PVR7 Methanolobus halotolerans
A0A7D5INX6 Methanolobus zinderi
A0A1Q9MX19 Lokiarchaeota archaeon

U

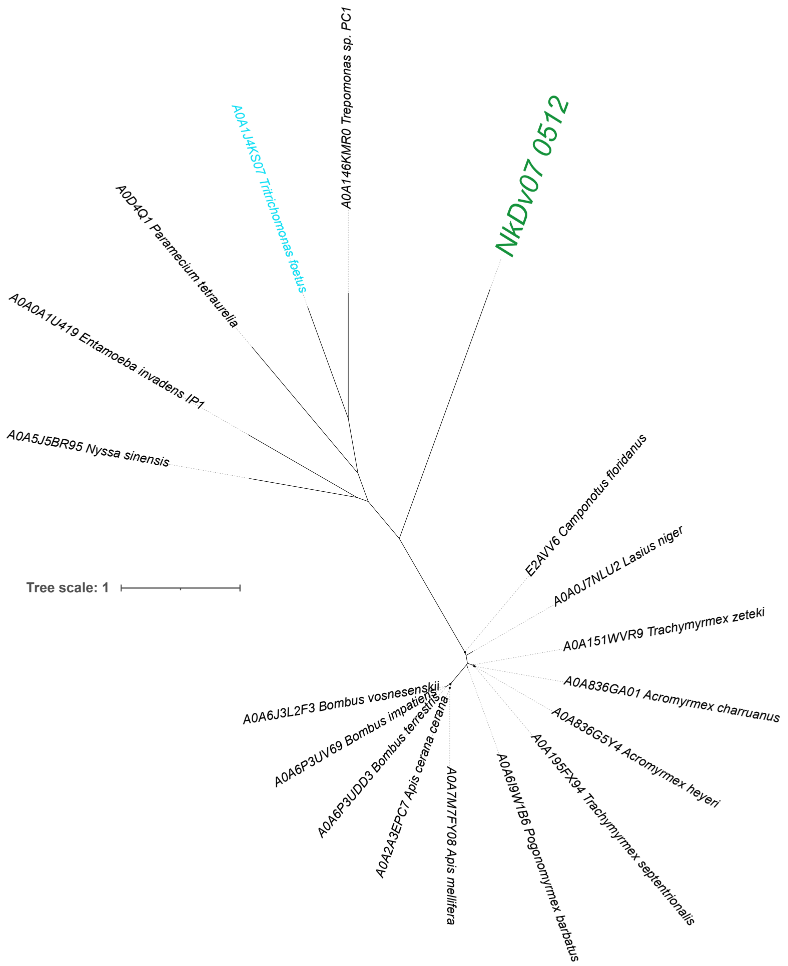
A0A146KMR0 Trepomonas sp. PC1
A0A1J4KS07 Tritrichomonas foetus
NkDv07 0512
A0D4Q1 Paramecium tetraurelia
A0A0A1U419 Entamoeba invadens IP1
A0A5J5BR95 Nyssa sinensis
Tree scale: 1
E2AVV6 Camponotus floridanus
A0A0J7NLU2 Lasius niger
A0A151WVR9 Trachymyrmex zeteki
A0A836GA01 Acromyrmex charruanus
A0A836G5Y4 Acromyrmex heyeri
A0A195FX94 Trachymyrmex septentrionalis
A0A6J3L2F3 Bombus vosnesenskii
A0A6P3UV69 Bombus impatiens
A0A6P3UDD3 Bombus terrestris
A0A2A3EPC7 Apis cerana cerana
A0A7M7FY08 Apis mellifera
A0A6I9W1B6 Pogonomyrmex barbatus

V

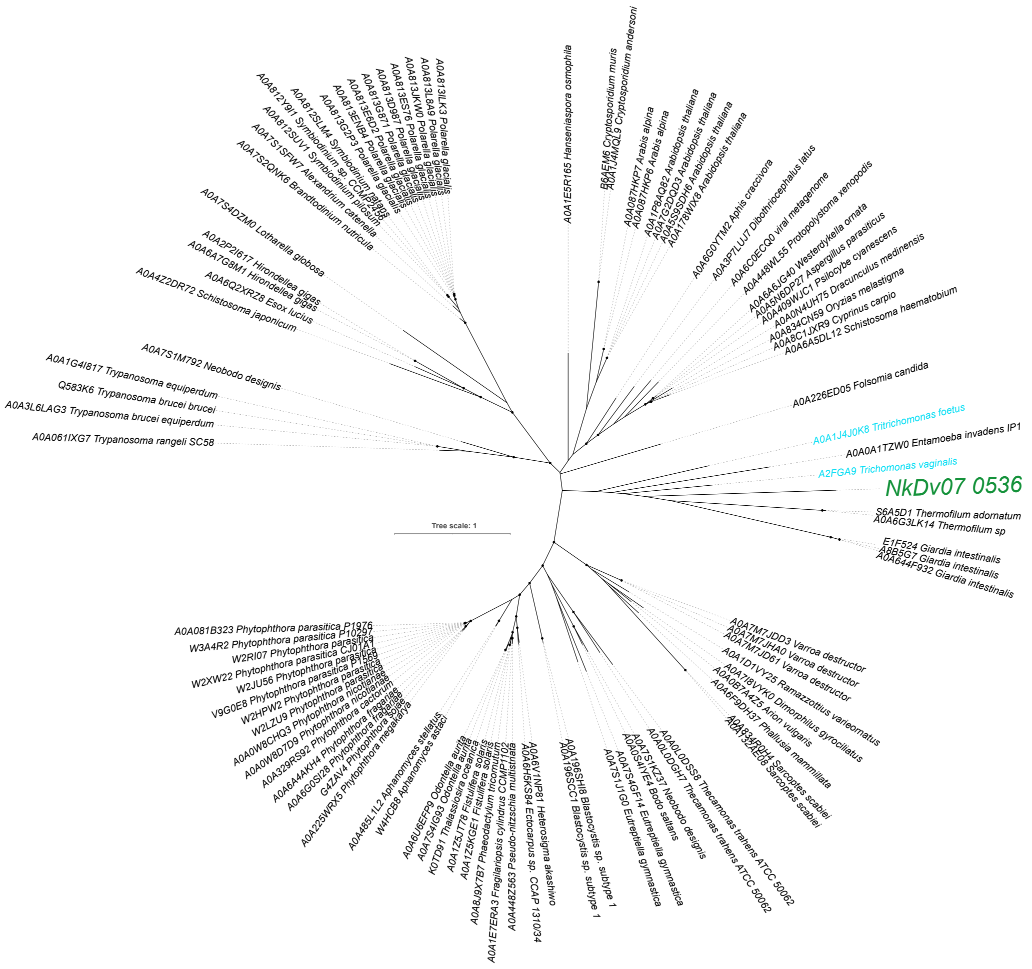
A0A1E5R165 Hanseniaspora osmophila
B6AEM6 Cryptosporidium muris
A0A1J4MQL9 Cryptosporidium andersoni
A0A087HKP7 Arabis alpina
A0A087HKP6 Arabis alpina
A0A1P8AQ82 Arabidopsis thaliana
A0A7G2DQD3 Arabidopsis thaliana
A0A5S9SDH6 Arabidopsis thaliana
A0A178WIX8 Arabidopsis thaliana
A0A813ILK3 Polarella glacialis
A0A813L8A9 Polarella glacialis
A0A813JKW0 Polarella glacialis
A0A813ES76 Polarella glacialis
A0A813D987 Polarella glacialis
A0A813G871 Polarella glacialis
A0A813E6D2 Polarella glacialis
A0A813ENB4 Polarella glacialis
A0A813G2P3 Polarella glacialis
A0A812SLM4 Symbiodinium natans
A0A812Y9I1 Symbiodinium sp. CCMP2456
A0A812SUV1 Symbiodinium pilosum
A0A7S1SFW7 Alexandrium catenella
A0A7S2QNK6 Brandtodinium nutricula
A0A7S4DZM0 Lotharella globosa
A0A2P2I617 Hirondellea gigas
A0A6A7G8M1 Hirondellea gigas
A0A6Q2XRZ8 Esox lucius
A0A4Z2DR72 Schistosoma japonicum
A0A7S1M792 Neobodo designis
A0A1G4I817 Trypanosoma equiperdum
Q583K6 Trypanosoma brucei brucei
A0A3L6LAG3 Trypanosoma brucei equiperdum
A0A061IXG7 Trypanosoma rangeli SC58
A0A6G0YTM2 Aphis craccivora
A0A3P7LUJ7 Dibothriocephalus latus
A0A6C0ECQ0 viral metagenome
A0A448WL55 Protopolystoma xenopodis
A0A6A6JG40 Westerdykella ornata
A0A5N6DP27 Aspergillus parasiticus
A0A409WJC1 Psilocybe cyanescens
A0A0N4UH75 Dracunculus medinensis
A0A834CN59 Oryzias melastigma
A0A8C1JXR9 Cyprinus carpio
A0A6A5DL12 Schistosoma haematobium
A0A226ED05 Folsomia candida
A0A1J4J0K8 Tritrichomonas foetus
A0A0A1TZW0 Entamoeba invadens IP1
A2FGA9 Trichomonas vaginalis
NkDv07 0536
S6A5D1 Thermofilum adornatum
A0A6G3LK14 Thermofilum sp
E1F524 Giardia intestinalis
A8B5G7 Giardia intestinalis
A0A644F932 Giardia intestinalis
Tree scale: 1
A0A7M7JDD3 Varroa destructor
A0A7M7JHA0 Varroa destructor
A0A7M7JD61 Varroa destructor
A0A1D1VY25 Ramazzottius varieornatus
A0A7I8VYK0 Dimorphilus gyrociliatus
A0A0B7A4Z5 Arion vulgaris
A0A6F9DH37 Phallusia mammillata
A0A834R0H4 Sarcoptes scabiei
A0A132AE08 Sarcoptes scabiei
A0A0L0DSS8 Thecamonas trahens ATCC 50062
A0A0L0DGH7 Thecamonas trahens ATCC 50062
A0A7S1KZ37 Neobodo designis
A0A0S4IYE4 Bodo saltans
A0A7S4GF14 Eutreptiella gymnastica
A0A7S1J1G0 Eutreptiella gymnastica
A0A196SHI8 Blastocystis sp. subtype 1
A0A196SCC1 Blastocystis sp. subtype 1
A0A6V1NP81 Heterosigma akashiwo
A0A6H5KS84 Ectocarpus sp. CCAP 1310/34
A0A448Z563 Pseudo-nitzschia multistriata
A0A1E7ERA3 Fragilariopsis cylindrus CCMP1102
A0A8J9X7B7 Phaeodactylum tricornutum
A0A1Z5KGE1 Fistulifera solaris
A0A1Z5JT78 Fistulifera solaris
K0TD91 Thalassiosira oceanica
A0A7S4IG93 Odontella aurita
A0A6U6EFP9 Odontella aurita
W4HCB8 Aphanomyces astaci
A0A485L1L2 Aphanomyces stellatus
A0A081B323 Phytophthora parasitica P1976
W3A4R2 Phytophthora parasitica P10297
W2RI07 Phytophthora parasitica
W2XW22 Phytophthora parasitica CJ01A1
W2JU56 Phytophthora parasitica
V9G0E8 Phytophthora parasitica P1569
W2HPW2 Phytophthora parasitica
W2LZU9 Phytophthora nicotianae
A0A0W8CHQ3 Phytophthora nicotianae
A0A0W8D7D9 Phytophthora nicotianae
A0A329RS92 Phytophthora cactorum
A0A6A4AKH4 Phytophthora fragariae
A0A6G0SI28 Phytophthora fragariae
G4ZAV4 Phytophthora sojae
A0A225WRX5 Phytophthora megakarya

W

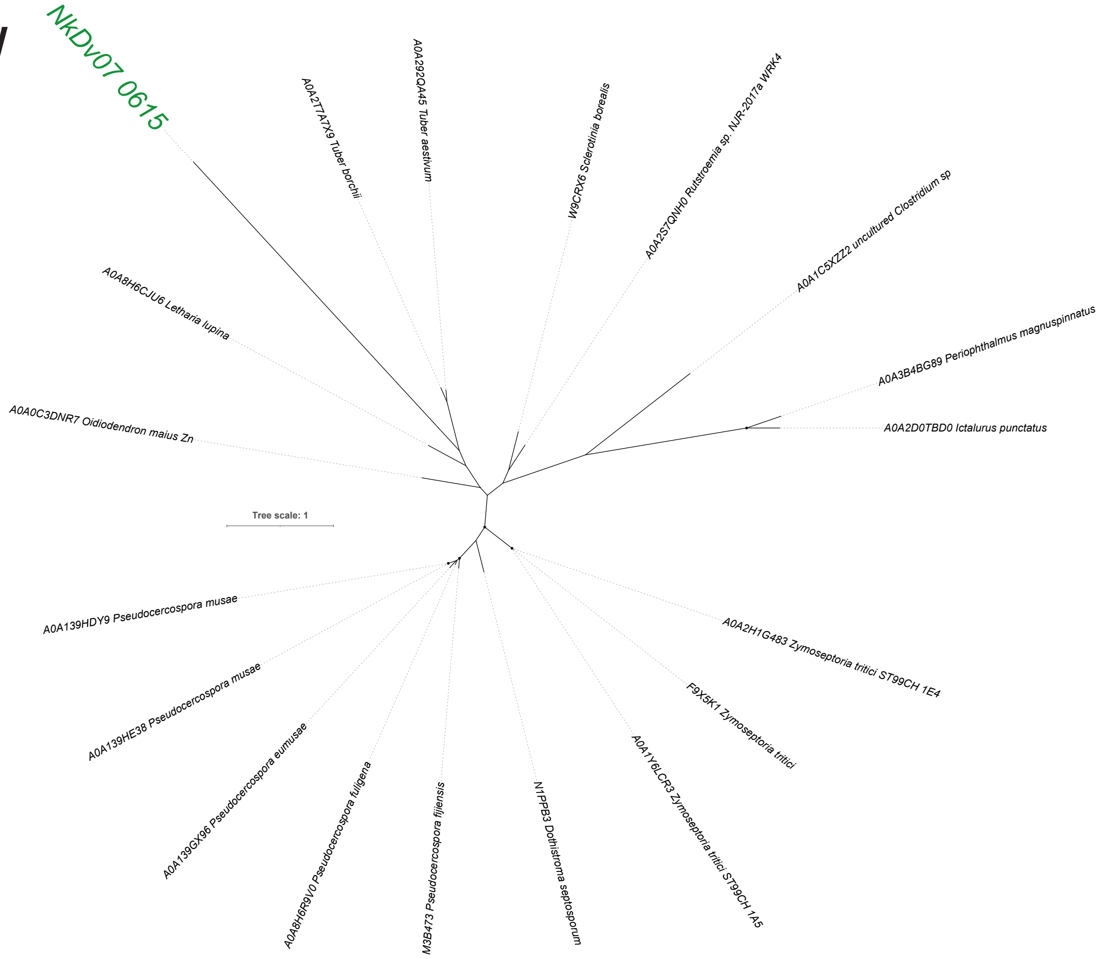
NkDv07 0615
A0A2T7A7X9 Tuber borchii
A0A292Q445 Tuber aestivum
W9CRX6 Sclerotinia borealis
A0A2S7QNH0 Rutstroemia sp. NJR-2017a WRK4
A0A1C5XZZ2 uncultured Clostridium sp
A0A3B4BG89 Periophthalmus magnuspinnatus
A0A2D0TBD0 Ictalurus punctatus
A0A8H6CJU6 Letharia lupina
A0A0C3DNR7 Oidiodendron maius Zn
Tree scale: 1
A0A139HDY9 Pseudocercospora musae
A0A139HE38 Pseudocercospora musae
A0A139GX96 Pseudocercospora eumusae
A0A8H6R9V0 Pseudocercospora fuligena
M3B473 Pseudocercospora fijiensis
N1PPB3 Dothistroma septosporum
A0A1Y6LCR3 Zymoseptoria tritici ST99CH 1A5
F9X5K1 Zymoseptoria tritici
A0A2H1G483 Zymoseptoria tritici ST99CH 1E4

X

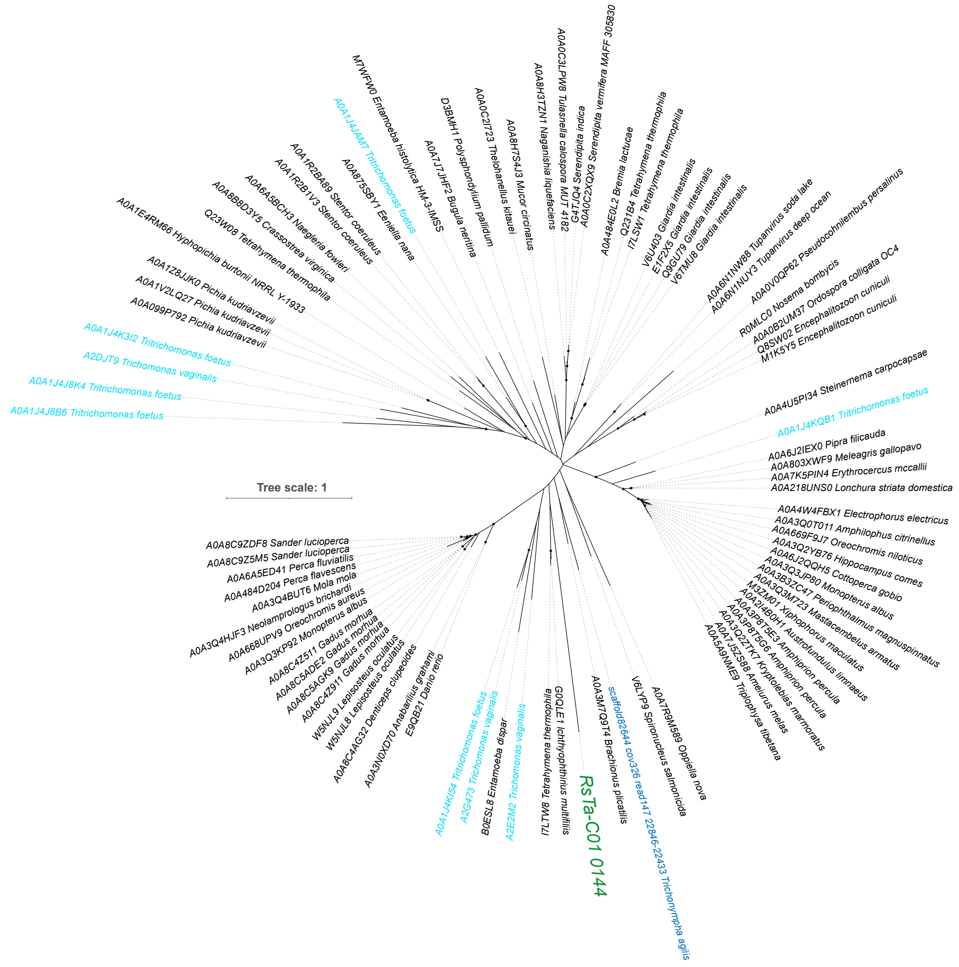
A0A1E4RM66 Hyphopichia burtonii NRRL Y-1933
A0A1Z8JJK0 Pichia kudriavzevii
A0A1V2LQ27 Pichia kudriavzevii
A0A099P792 Pichia kudriavzevii
A0A1J4K3I2 Tritrichomonas foetus
A2DJT9 Trichomonas vaginalis
A0A1J4J8K4 Tritrichomonas foetus
A0A1J4J8B6 Tritrichomonas foetus
Q23W08 Tetrahymena thermophila
A0A8B8D3Y5 Crassostrea virginica
A0A6A5BCH3 Naegleria fowleri
A0A1R2B1V3 Stentor coeruleus
A0A1R2BA89 Stentor coeruleus
A0A875SBY1 Eeniella nana
A0A1J4JAM7 Tritrichomonas foetus
MTWFW0 Entamoeba histolytica HM-3-IMSS
A0A7J7JHF2 Bugula neritina
D3BMH1 Polysphondylium pallidum
A0A0C2I723 Thelohanellus kitauei
A0A8H7S4J3 Mucor circinatus
A0A8H3TZN1 Naganishia liquefaciens
A0A0C3LPW8 Tulasnella calospora MUT 4182
A0A0C2XQX9 Serendipita vermifera MAFF 305830
A0A4E0L2 Bremia lactucae
Q23TB4 Tetrahymena thermophila
I7LSW1 Tetrahymena thermophila
V6U403 Giardia intestinalis
E1F2X5 Giardia intestinalis
Q9GU79 Giardia intestinalis
V6TMU8 Giardia intestinalis
A0A6N1NW88 Tupanvirus soda lake
A0A6N1NUY3 Tupanvirus deep ocean
A0A0V0QP62 Pseudocohnilembus persalinus
R0MLC0 Nosema bombycis
A0A0B2UM37 Ordospora colligata OC4
Q8SW02 Encephalitozoon cuniculi
M1K5Y5 Encephalitozoon cuniculi
A0A4U5PI34 Steinernema carpocapsae
A0A1J4KQB1 Tritrichomonas foetus
A0A6J2IEX0 Pipra filicauda
A0A803XWF9 Meleagris gallopavo
A0A7K5PIN4 Erythrocercus mccallii
A0A218UNS0 Lonchura striata domestica
A0A4W4FBX1 Electrophorus electricus
A0A3Q0T011 Amphilophus citrinellus
A0A669F9J7 Oreochromis niloticus
A0A3Q2YB76 Hippocampus comes
A0A6J2QQH5 Cottoperca gobio
A0A3Q3JP80 Monopterus albus
A0A3B3ZC47 Periophthalmus magnuspinnatus
A0A3Q3M723 Mastacembelus armatus
A0A3B5BT5E3 Amphiprion percula
A0A4W4FBX1
A0A3P8T9G6 Amphiprion percula
A0A3Q2ZTK7 Kryptolebias marmoratus
A0A7J5Z588 Ameiurus melas
A0A5A9NME9 Triplophysa tibetana
Tree scale: 1
A0A8C9ZDF8 Sander lucioperca
A0A8C9Z5M5 Sander lucioperca
A0A6A5ED41 Perca fluviatilis
A0A484D204 Perca flavescens
A0A3Q4BUT6 Mola mola
A0A3Q4HJF3 Neolamprologus brichardi
A0A668UPV9 Oreochromis aureus
A0A3Q3KP92 Monopterus albus
A0A8C4Z511 Gadus morhua
A0A8C5ADE2 Gadus morhua
A0A8C5AGK9 Gadus morhua
A0A8C4Z911 Gadus morhua
W5NJL9 Lepisosteus oculatus
W5NJL8 Lepisosteus oculatus
A0A8C4AG32 Denticeps clupeoides
A0A3N0XD70 Anabarilius grahami
E9QB21 Danio rerio
A0A1J4KI54 Tritrichomonas foetus
A2G473 Trichomonas vaginalis
B0ESL8 Entamoeba dispar
A2E2M2 Trichomonas vaginalis
I7LTW8 Tetrahymena thermophila
G0QLE1 Ichthyophthirius multifiliis
RsTa-C01 0144
A0A3M7Q9T4 Brachionus plicatilis
scaffold82644 cov326 read147 22846-22433 Trichonympha agilis
V6LYP9 Spironucleus salmonicida
A0A7R9M589 Oppiella nova

Y

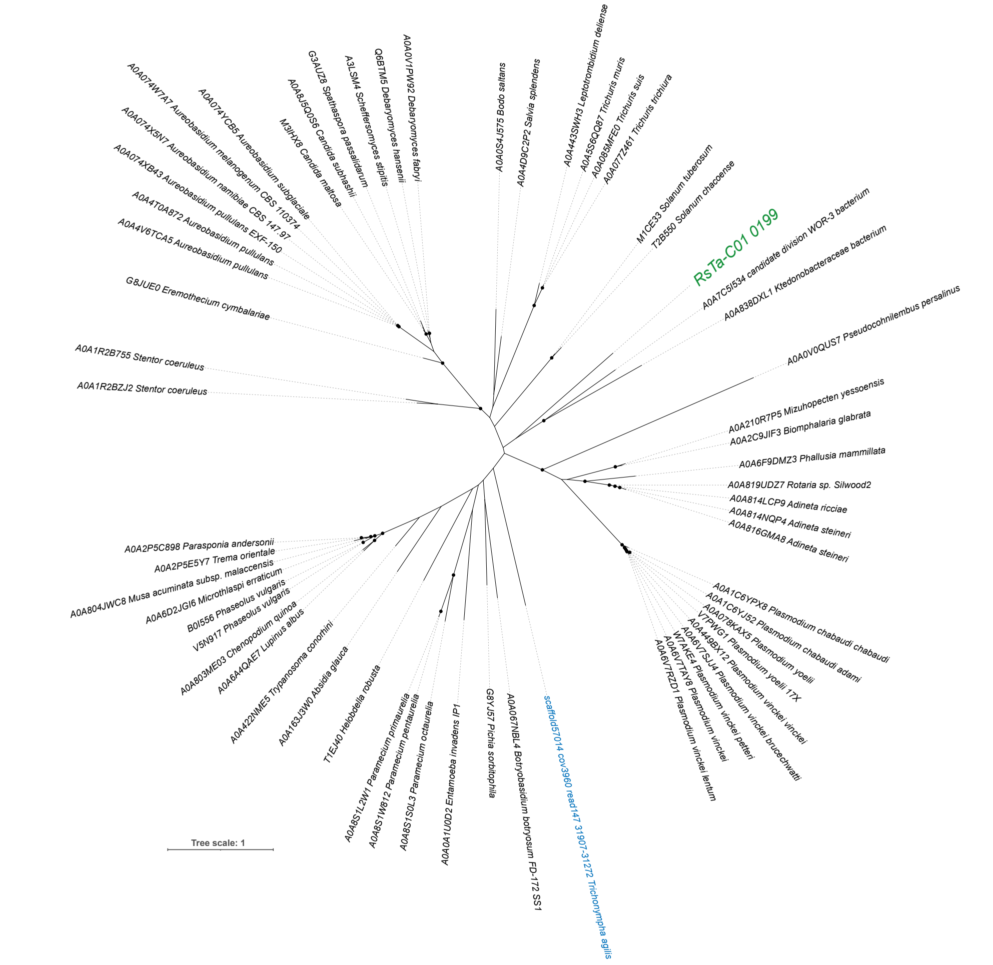

A0A074W7A7 Aureobasidium subglaciale
A0A074YCB5 Aureobasidium melanogenum CBS 110374
A0A074XSN7 Aureobasidium namibiae CBS 147.97
A0A074XB43 Aureobasidium pullulans EXF-150
A0A4T0A872 Aureobasidium pullulans
A0A4V6TCA5 Aureobasidium pullulans
G8JUE0 Eremothecium cymbalariae
A0A1R2B755 Stentor coeruleus
A0A1R2BZJ2 Stentor coeruleus
M3HX8 Candida maltosa
A0A8J5QOS6 Candida subhashii
G3AUZ8 Spathaspora passalidarum
A3LSM4 Scheffersomyces stipitis
Q6BTM5 Debaryomyces hansenii
A0A0V1PW92 Debaryomyces fabryi
A0A0S4J575 Bodo saltans
A0A4D9C2P2 Salvia splendens
A0A443SWH3 Leptotrombidium deliense
A0A5S6QQ87 Trichuris muris
A0A085MFE0 Trichuris suis
A0A077Z461 Trichuris trichiura
M1CE33 Solanum tuberosum
T2B550 Solanum chacoense
RsTa-C01 0199
A0A7C5I534 candidate division WOR-3 bacterium
A0A838DXL1 Ktedonobacteraceae bacterium
A0A0V0QUS7 Pseudocohnilembus persalinus
A0A210R7P5 Mizuhopecten yessoensis
A0A2C9JIF3 Biomphalaria glabrata
A0A6F9DMZ3 Phallusia mammillata
A0A819UDZ7 Rotaria sp. Silwood2
A0A814LCP9 Adineta ricciae
A0A814NQP4 Adineta steineri
A0A816GMA8 Adineta steineri
A0A1C6YPX8 Plasmodium chabaudi chabaudi
A0A1C6YJS2 Plasmodium chabaudi adami
A0A077BKX5 Plasmodium yoelii
V7PWG1 Plasmodium yoelii 17X
A0A449BX12 Plasmodium vinckei vinckei
A0A6V7SJJ4 Plasmodium vinckei brucechwatti
W7AKE4 Plasmodium vinckei petteri
A0A6V7TAY8 Plasmodium vinckei
A0A6V7RZD1 Plasmodium vinckei lentum
A0A2P5C898 Parasponia andersonii
A0A2P5E5Y7 Trema orientale
A0A804JWC8 Musa acuminata subsp. malaccensis
A0A6D2JGI6 Microthlaspi erraticum
B0I556 Phaseolus vulgaris
V5N917 Phaseolus vulgaris
A0A803ME03 Chenopodium quinoa
A0A6A4QAE7 Lupinus albus
A0A422NME5 Trypanosoma conorhini
A0A163J3W0 Absidia glauca
T1EJ40 Helobdella robusta
A0A8S1L2W1 Paramecium primaurelia
A0A8S1W812 Paramecium pentaurelia
A0A8S1S0L3 Paramecium octaurelia
A0A0A1U0D2 Entamoeba invadens IP1
G8YJ57 Pichia sorbitophila
A0A067NBL4 Botryobasidium botryosum FD-172 SS1
scaffold57014 cov3960 read147 31907-31272 Trichonympha agilis
Tree scale: 1

Z

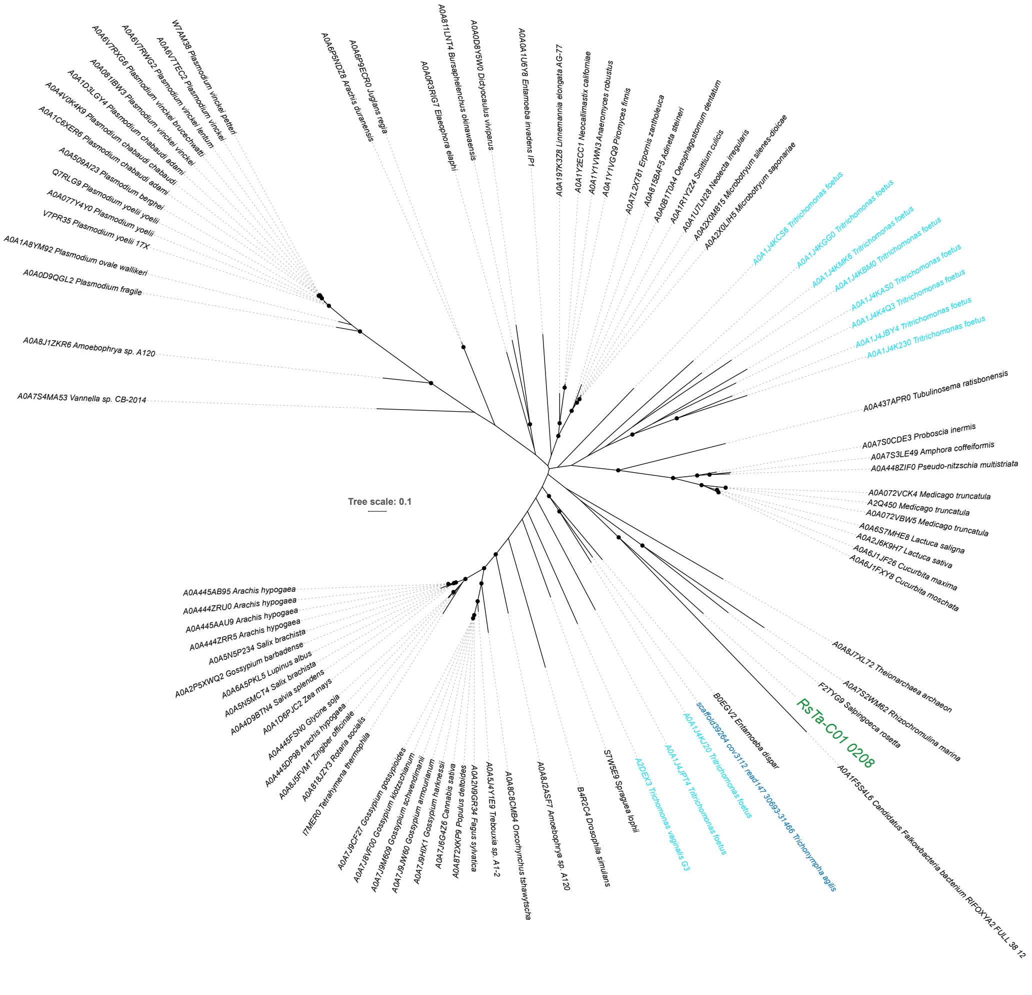

W7AM98 Plasmodium vinckei petteri
A0A6V7TEC2 Plasmodium vinckei
A0A6V7RWG2 Plasmodium vinckei lentum
A0A6V7RXG6 Plasmodium vinckei brucechwatti
A0A081IBW3 Plasmodium vinckei vinckei
A0A1D3LGY4 Plasmodium chabaudi chabaudi
A0A4V0K4K9 Plasmodium chabaudi adami
A0A1C6XER6 Plasmodium chabaudi
A0A509AJ23 Plasmodium berghei
Q7RLG9 Plasmodium yoelii yoelii
A0A077Y4Y0 Plasmodium yoelii
V7PR35 Plasmodium yoelii 17X
A0A1A8YM92 Plasmodium ovale wallikeri
A0A0D9QGL2 Plasmodium fragile
A0A8J1ZKR6 Amoebophrya sp. A120
A0A7S4MA53 Vannella sp. CB-2014
A0A6P5N2Z8 Arachis duranensis
A0A6P9ECR0 Juglans regia
A0A811LNT4 Bursaphelenchus okinawaensis
A0A0R3RGT7 Elaeophora elaphi
A0A0D8Y5W0 Dictyocaulus viviparus
A0A0A1U6Y8 Entamoeba invadens IP1
A0A197K328 Linnemannia elongata AG-77
A0A1Y2ECC1 Neocallimastix californiae
A0A1Y1VWI3 Anaeromyces robustus
A0A1Y1VGQ9 Piromyces finnis
A0A1L2X781 Erpornis zantholeuca
A0A815BAF5 Adineta steineri
A0A081T044 Oesophagostomum dentatum
A0A1R1YZ24 Smittium culicis
A0A1U7LN28 Neolecta irregularis
A0A2X0M615 Microbotryum silenes-dioicae
A0A2X0LJH5 Microbotryum saponariae
A0A1J4KCS6 Tritrichomonas foetus
A0A1J4KGG0 Tritrichomonas foetus
A0A1J4KMK6 Tritrichomonas foetus
A0A1J4KBM9 Tritrichomonas foetus
A0A1J4KAS0 Tritrichomonas foetus
A0A1J4K4Q3 Tritrichomonas foetus
A0A1J4JBY4 Tritrichomonas foetus
A0A1J4K230 Tritrichomonas foetus
A0A437APR0 Tubulinosema ratisbonensis
A0A7S0CDE3 Proboscia inermis
A0A7S3LE49 Amphora coffeiformis
A0A448ZIF0 Pseudo-nitzschia multistriata
A0A072VCK4 Medicago truncatula
A2Q450 Medicago truncatula
A0A072VBW5 Medicago truncatula
A0A6S7MHE8 Lactuca saligna
A0A2J6K9H7 Lactuca sativa
A0A6J1JF26 Cucurbita maxima
A0A6J1FXY8 Cucurbita moschata
A0A8J7XL72 Theionarchaea archaeon
A0A7S2WM62 Rhizochromulina marina
F2TYG9 Salpingoeca rosetta
RsTa-C01 0208
A0A1F5S4L6 Candidatus Falkowbacteria bacterium RIFOXYA2 FULL 38 12
A0A445AB95 Arachis hypogaea
A0A444ZRU0 Arachis hypogaea
A0A445AAU9 Arachis hypogaea
A0A444ZRR5 Arachis hypogaea
A0A5N5P234 Salix brachista
A0A2P5XWQ2 Gossypium barbadense
A0A6A5PKL5 Lupinus albus
A0A5N5MCT4 Salix brachista
A0A4D9BTN4 Salvia splendens
A0A1D6PJC2 Zea mays
A0A445FSN0 Glycine soja
A0A445DP98 Arachis hypogaea
A0A8J5FVM1 Zingiber officinale
A0A818JZY3 Rotaria socialis
I7MER0 Tetrahymena thermophila
A0A7J9CF27 Gossypium gossypioides
A0A7J8VF00 Gossypium klotzschianum
A0A7J9M609 Gossypium schwendimanii
A0A7J8JW60 Gossypium armourianum
A0A7J9HIX1 Gossypium harknessii
A0A7J6G426 Cannabis sativa
A0A8T2XKP9 Populus deltoides
A0A2N9GR34 Fagus sylvatica
A0A5J4YIE9 Trebouxia sp. A1-2
A0A8C8MBI4 Oncorhynchus tshawytscha
A0A8J2ASF7 Amoebophrya sp. A120
B4R2C4 Drosophila simulans
S7W6E9 Spraguea lophii
A2DEX3 Trichomonas vaginalis G3
A0A1J4JPT4 Tritrichomonas foetus
A0A1J4KV20 Tritrichomonas foetus
scaffold39284 cov3112 read147 30693-31466 Trichonympha agilis
B0EGV2 Entamoeba dispar
Tree scale: 0.1

AA

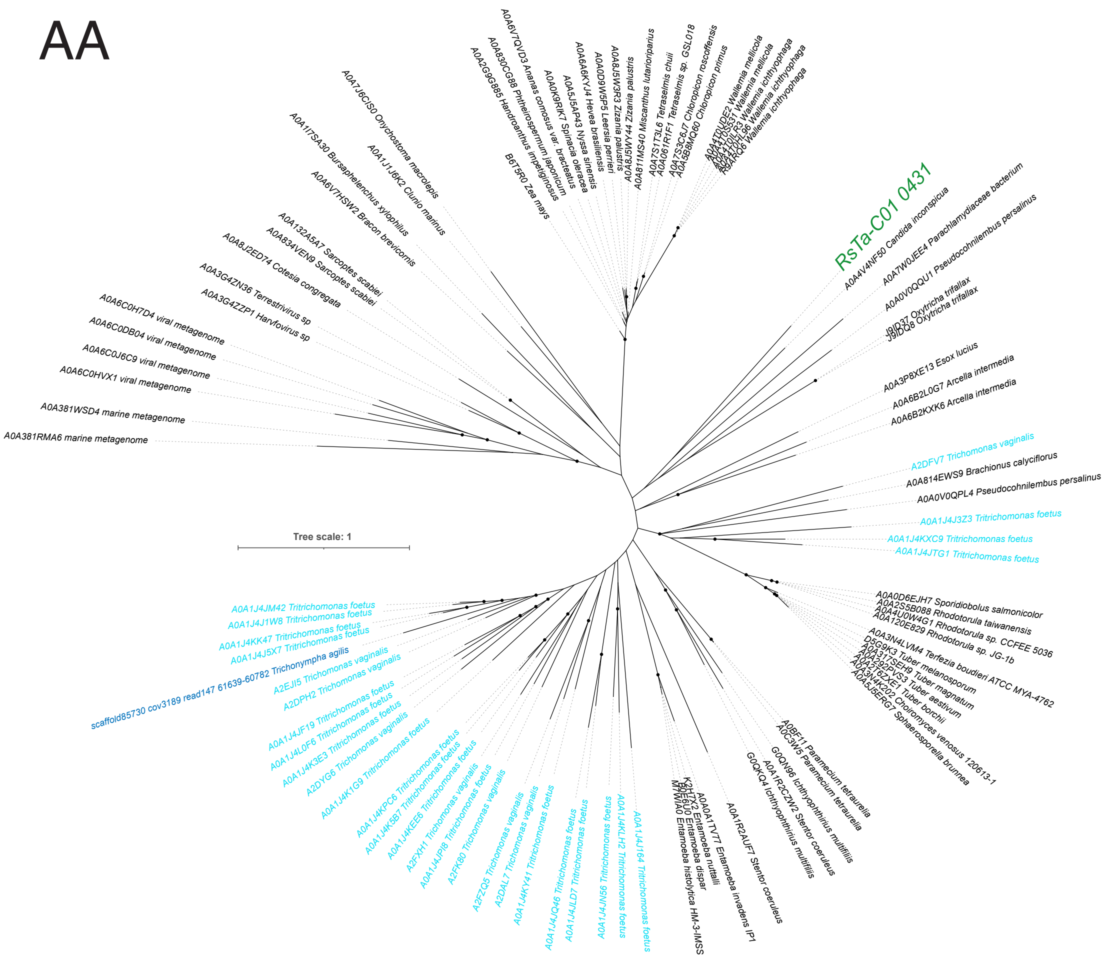

AB

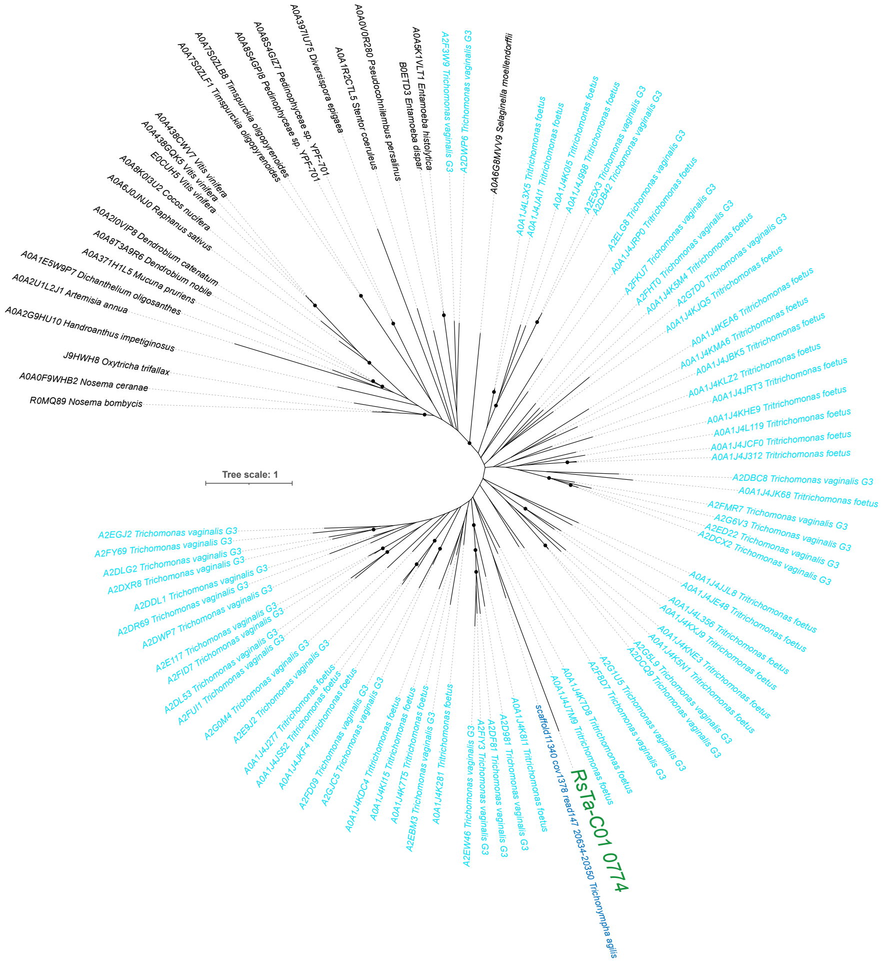

AC

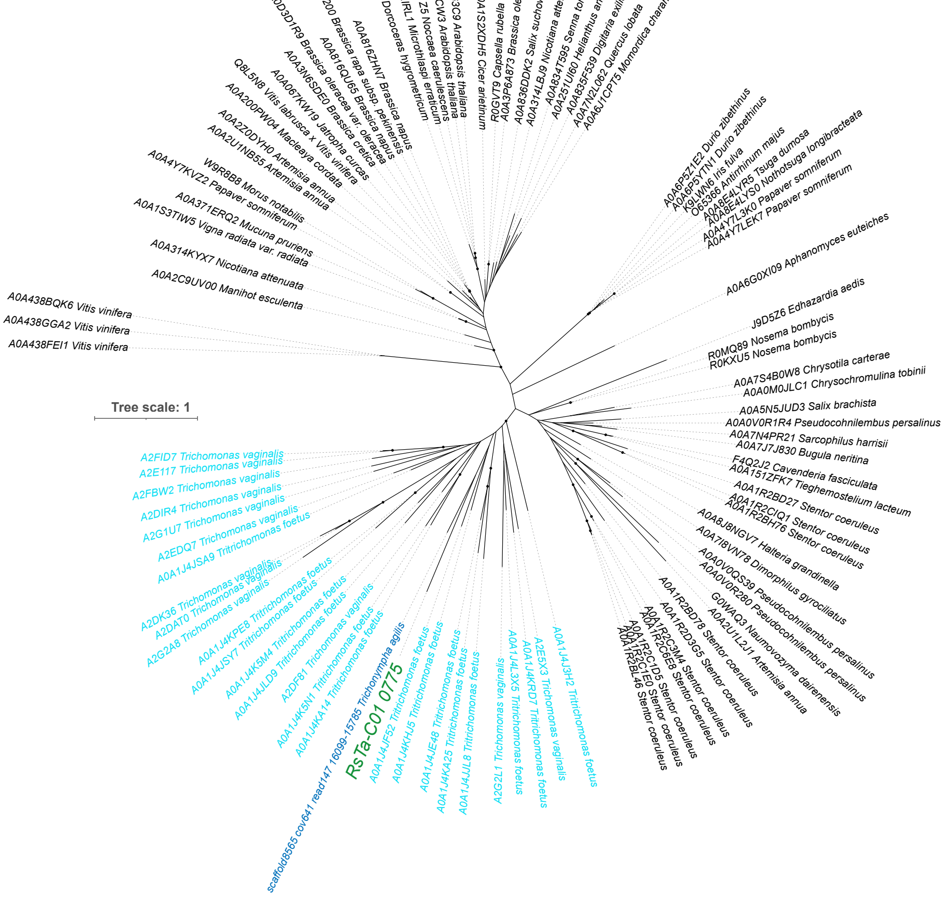
Tree scale: 1
RsTa-C01 0775
scaffold8565 cov641 read147 16099-15785 Trichonympha agilis
A0A438BQK6 Vitis vinifera
A0A438GGA2 Vitis vinifera
A0A438FEI1 Vitis vinifera
A0A6G0XI09 Aphanomyces euteiches
J9D5Z6 Edhazardia aedis
R0MQ89 Nosema bombycis
R0KXU5 Nosema bombycis

AD

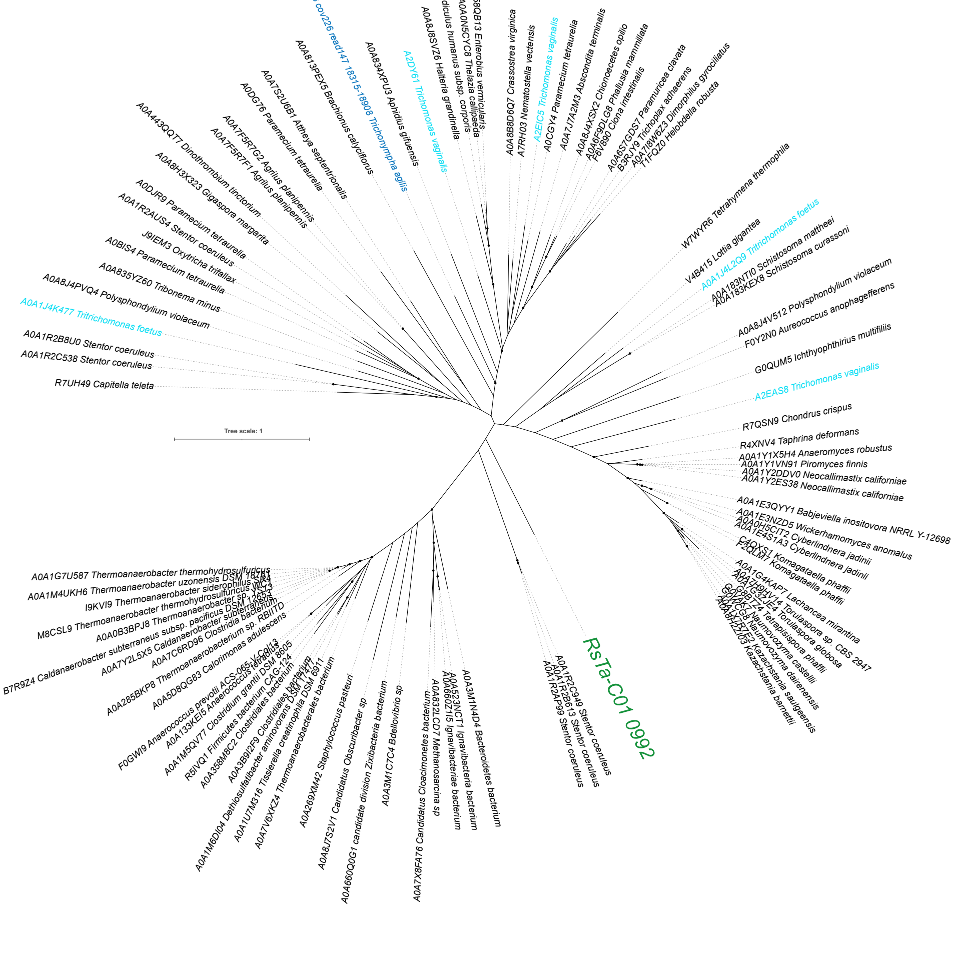
Tree scale: 1
RsTa-C01 0992
R7UH49 Capitella teleta
G0QUM5 Ichthyophthirius multifiliis
A2EAS8 Trichomonas vaginalis
R7QSN9 Chondrus crispus
R4XNV4 Taphrina deformans

Figure S9. Phylogenetic positions of eukaryotic-like genes contained in the genomes of CfP3-15, NkDv07, and RsTa-C01. The following genes were used as queries to identify homologues as reference sequences. (**A**) CfP3-15_0049. (**B**) CfP3-15_0165. (**C**) CfP3-15_0235. (**D**) CfP3-15_0282. (**E**) CfP3-15_0445. (**F**) CfP3-15_0456. (**G**) CfP3-15_0548. (**H**) CfP3-15_0552. (**I**) CfP3-15_0565. (**J**) CfP3-15_0581. (**K**) CfP3-15_0591. (**L**) CfP3-15_0627. (**M**) CfP3-15_0653. (**N**) CfP3-15_0780. (**O**) CfP3-15_0790. (**P**) CfP3-15_0852. (**Q**) CfP3-15_0856. (**R**) CfP3-15_0934. (**S**) NkDv07_0114. (**T**) NkDv07_0121. (**U**) NkDv07_0512. (**V**) NkDv07_0536. (**W**) NkDv07_0615. (**X**) RsTa-C01_0144. (**Y**) RsTa-C01_0199. (**Z**) RsTa-C01_0208. (**AA**) RsTa-C01_0431. (**AB**) RsTa-C01_0774. (**AC**) RsTa-C01_0775. (**AD**) RsTa-C01_0992. Highly supported nodes (SH-aLRT value ≥ 80% and ultrafast bootstrap support value ≥ 95%) are highlighted with closed circles. The sequence IDs highlighted by green, blue, and light blue correspond to the proteins of the endosymbiotic *Clostridia* used to find homologs, proteins of the protist hosts, and proteins from parabasalid protists, respectively.

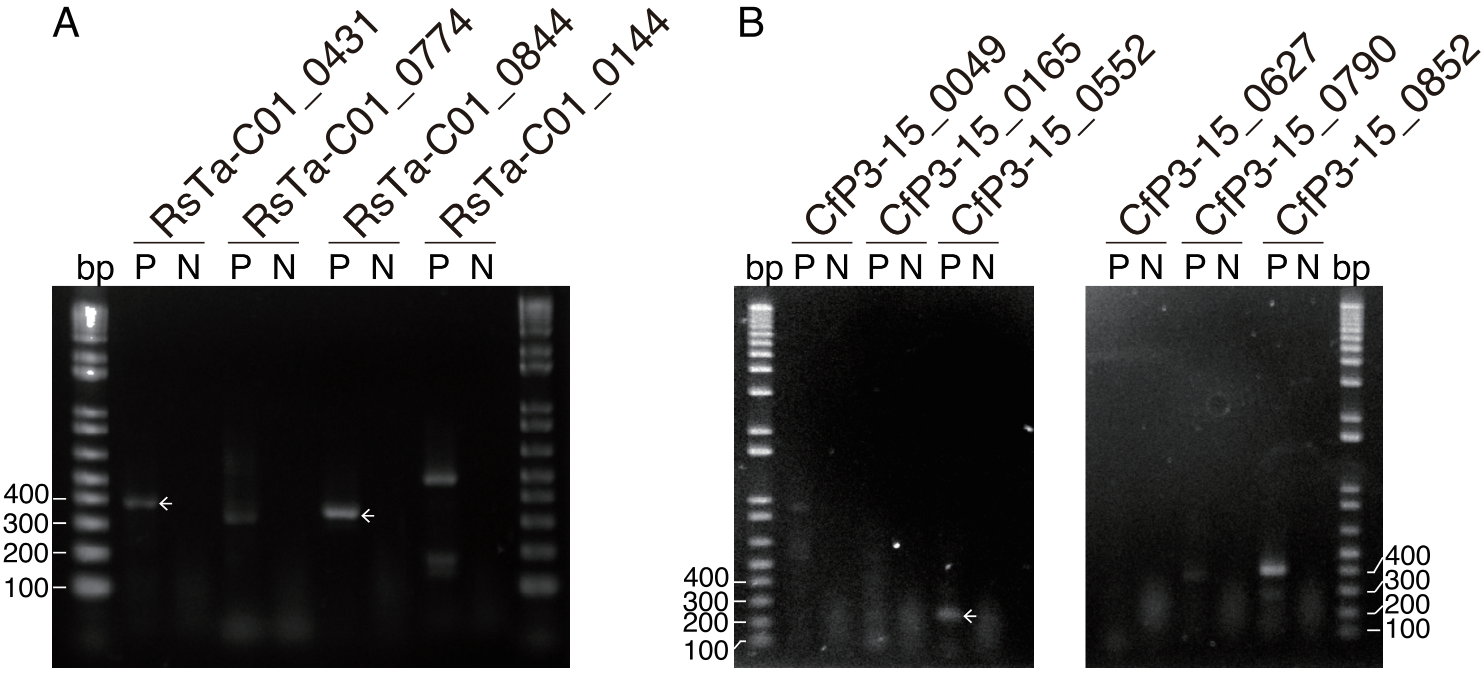

Figure S10. RT-PCR analysis of eukaryotic-like protein-coding genes. Agarose gel electrophoresis of RT-PCR products from total RNA of *R. speratus* guts (**A**) and *C. formosanus* guts (**B**). N, negative control (RNA samples without reverse transcription); P, products of RT-PCR using primer sets specific to the respective eukaryotic-like protein-coding genes (see Table S2). The amplification products of eukaryotic-like protein-coding genes are indicated by arrows, which were confirmed by Sanger sequencing.

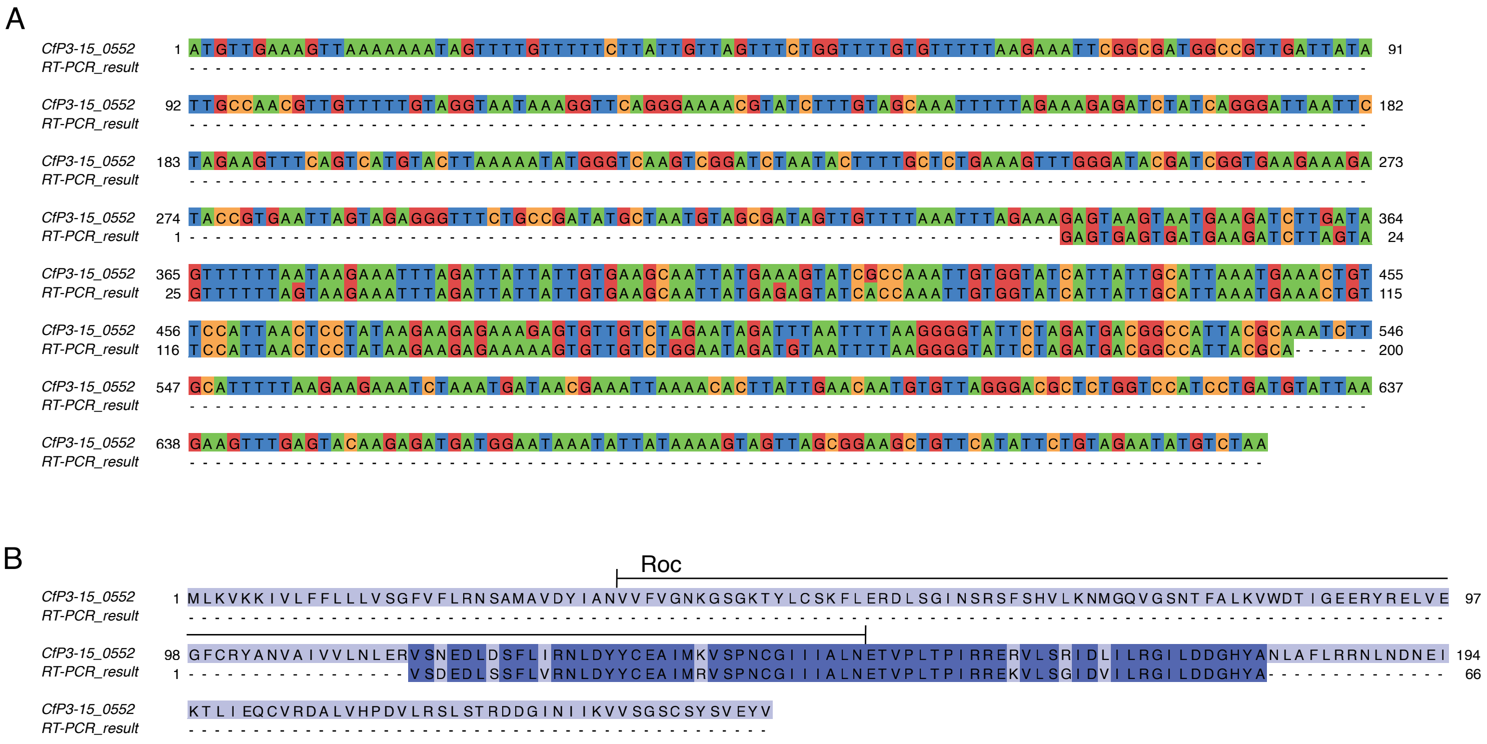

Figure S11. Comparison of a eukaryotic-like gene, CfP3-15_0552, in the CfP3-15 genome and that obtained by RT-PCR from *C. formosanus* guts. (**A**) Nucleotide sequences. (**B**) Amino acid sequences of **A**.

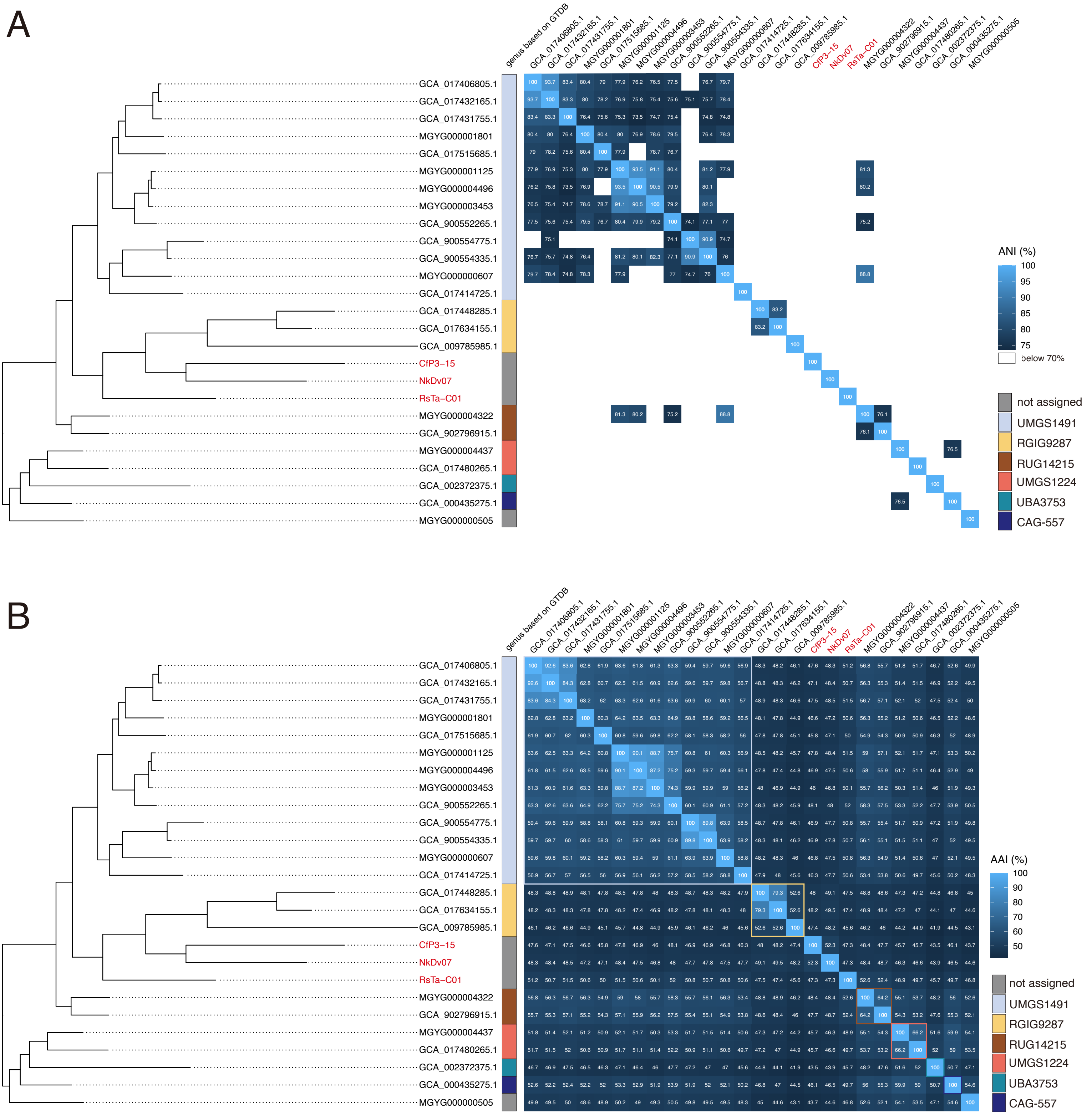

Figure S12. Pairwise ANI (**A**) and AAI (**B**) heatmap among the genomes in the small genome clade. The phylogenetic tree of the small genome clade is shown. In **B**, the genus-level clade based on GTDB r207 are indicated.

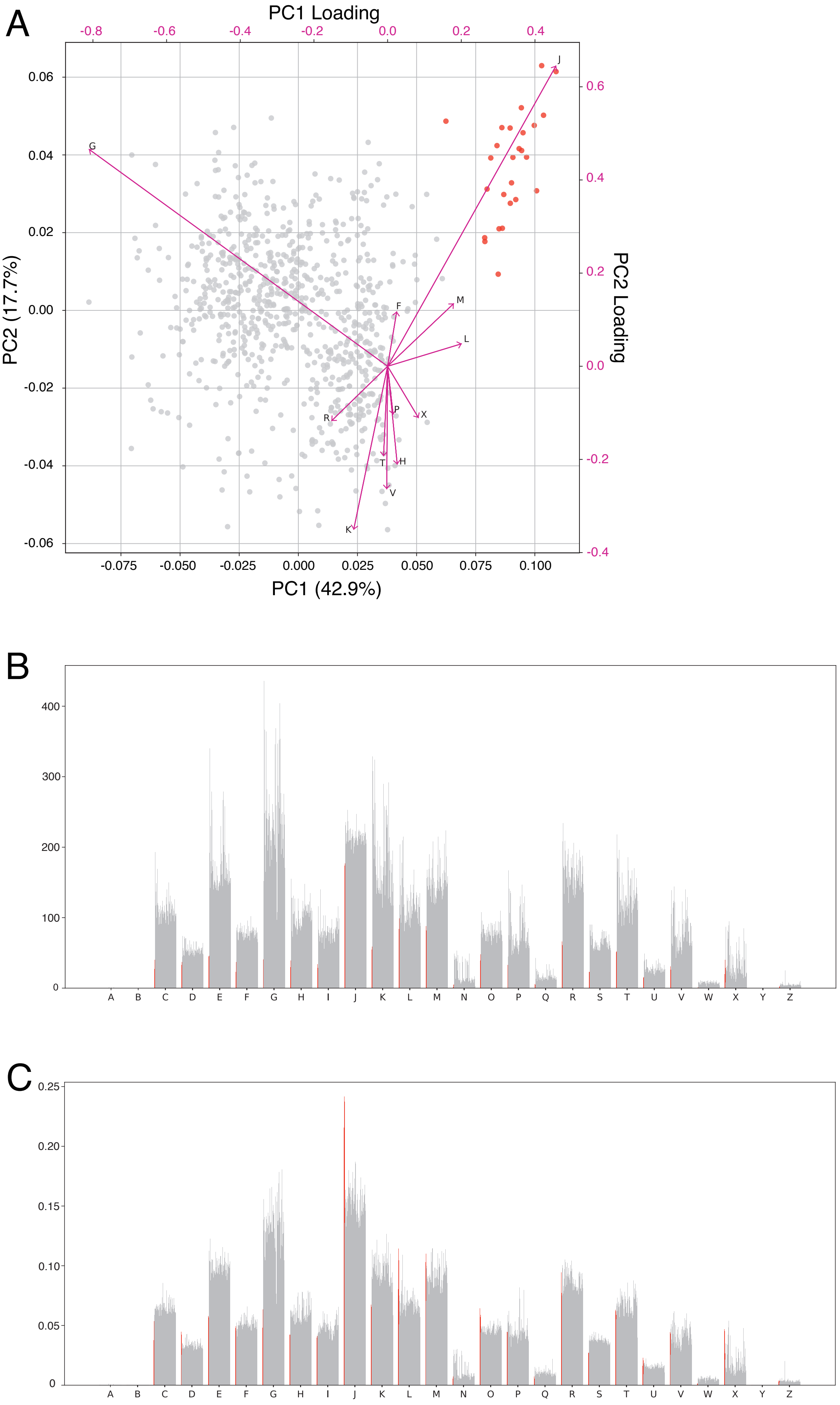

Figure S13. Principal component analysis of genomes of “Acutalibacteraceae” (Table S3) based on the relative abundance of the COG functional categories (**A**) and comparison of the number (**B**) and ratio (**C**) of the COG functional categories. Genomes belonging to the small genome clade are highlighted with red. Arrows indicate the loadings of each functional category. Several arrows are omitted to improve visibility. See also the legend to Fig. S5.

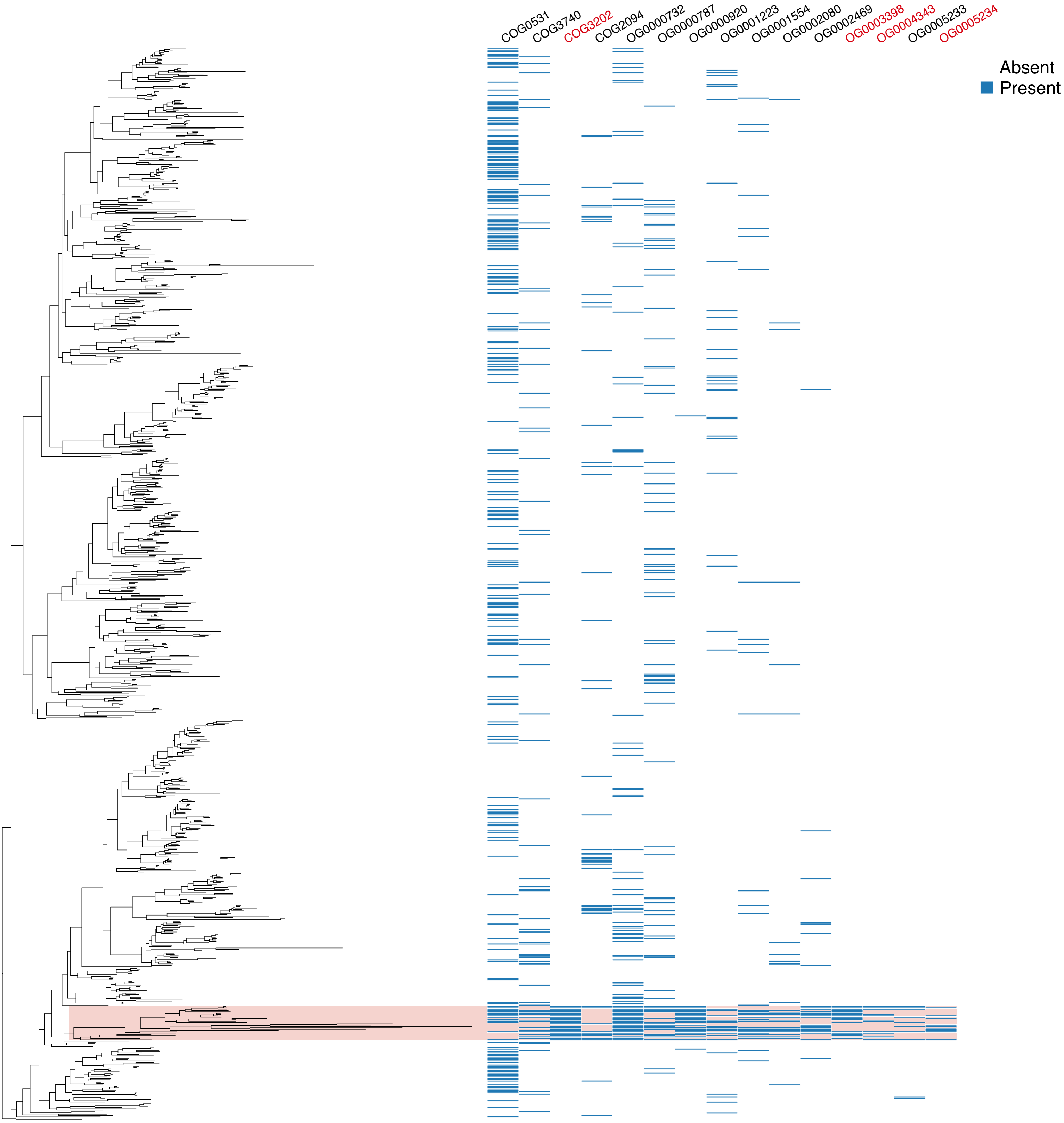

Figure S14. Distribution of genes acquired by the last common ancestor of the small genome clade, predicted in the gene flux analysis. The phylogenetic tree corresponds to that shown in Fig. 5A with the small genome clade being shaded in red.

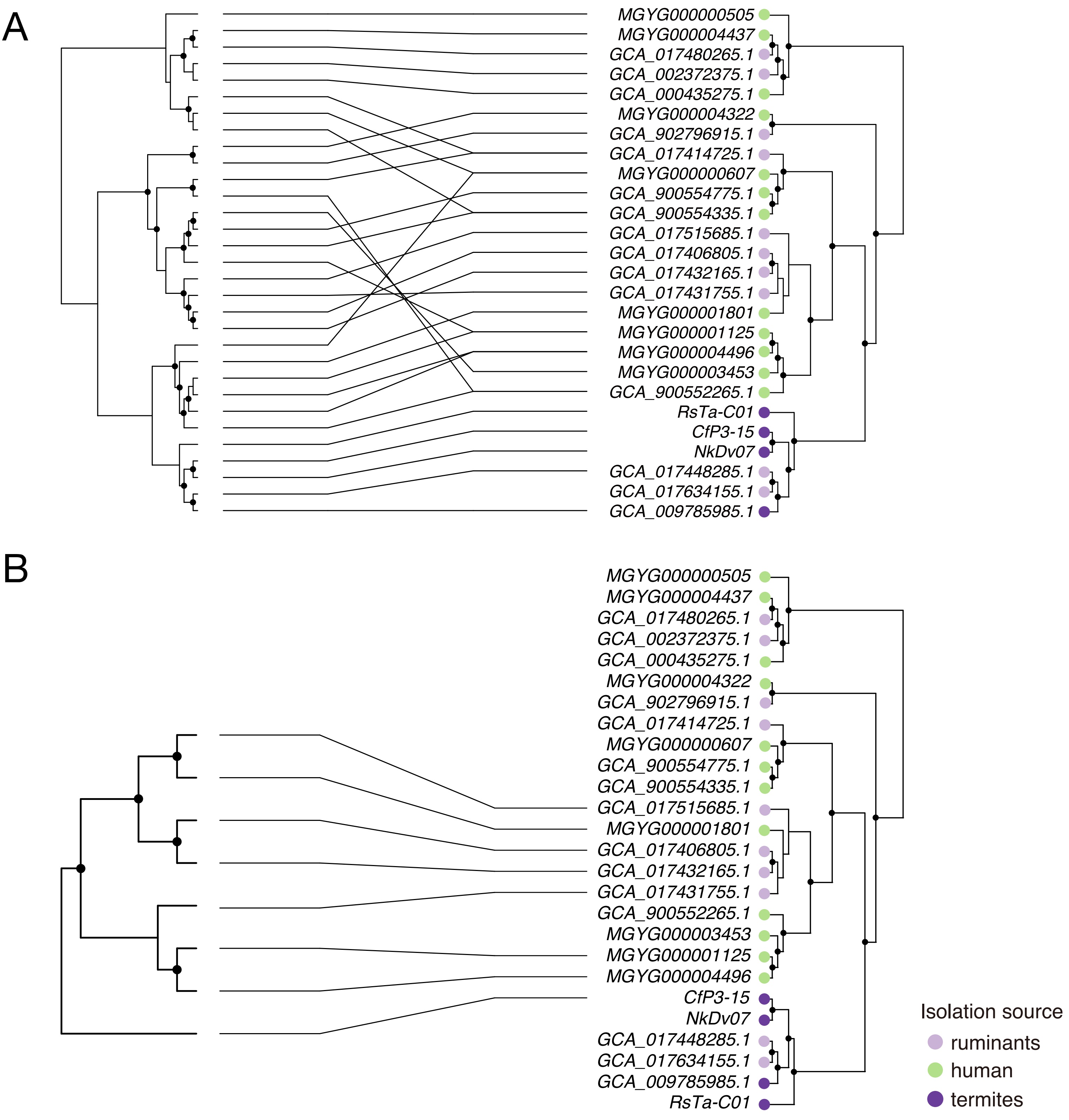

Figure S15. Comparison between the phylogenomic tree of the small genome clade and the gene tree of their ATP/ADP translocase. (**A**) Comparison of the *Clostridia* ATP/ADP translocase I (left) with the phylogenomic tree (right). (**B**) Comparison of the *Clostridia* ATP/ADP translocase II (left) and the phylogenomic tree (right). Highly supported nodes (SH-aLRT value ≥ 80% and ultrafast bootstrap support value ≥ 95%) are indicated with a closed circle.

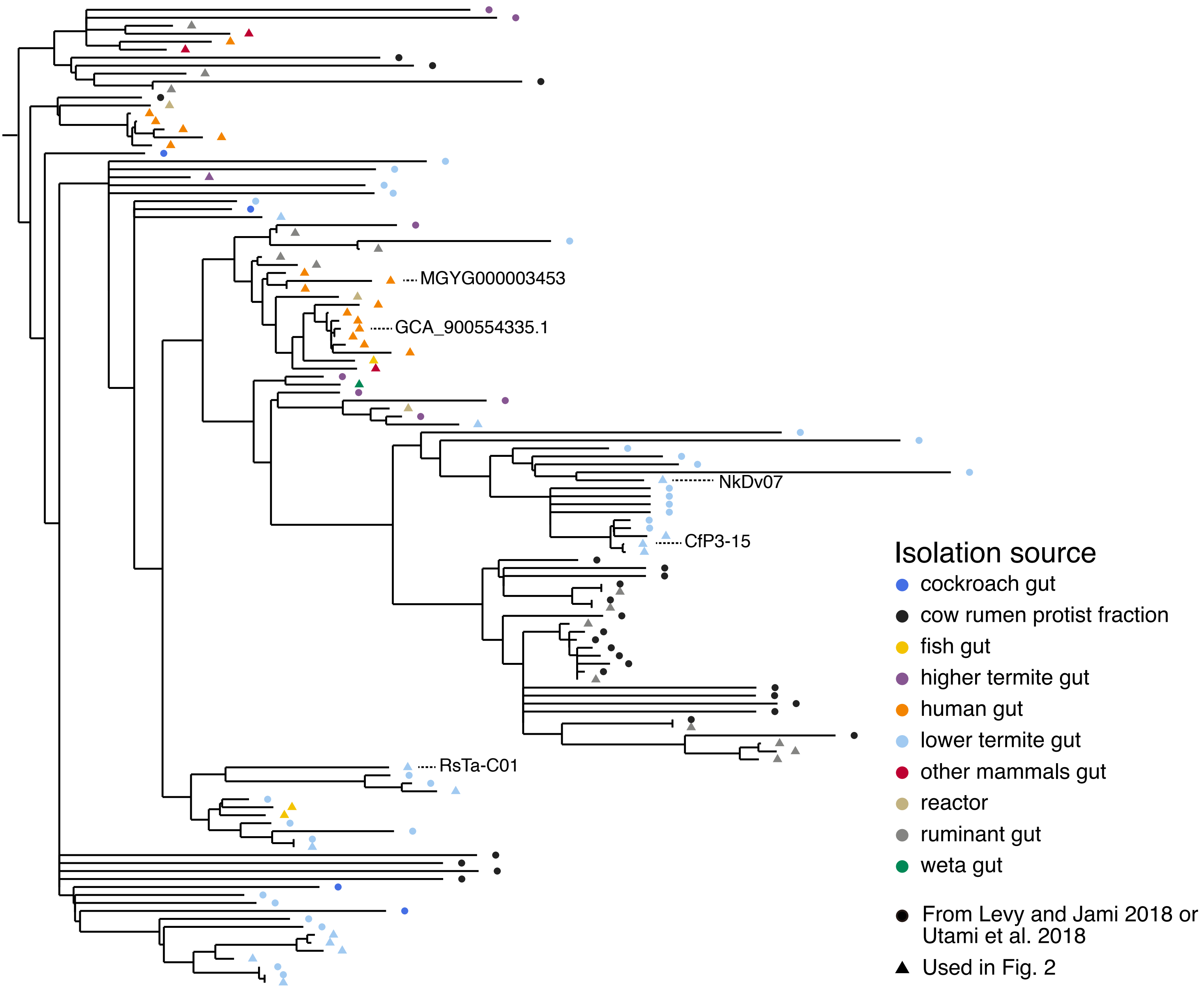

Figure S16. Phylogenetic positions of 16S rRNA gene amplicon sequences derived from the protistan fraction of a cattle rumen [31] and the entire guts of termites and cockroaches [32].
